# Supplementary material for: Benefit-risk analysis of maintaining essential Reproductive, Maternal, Newborn, and Child Health (RMNCH) services against risk of COVID-19 infection
Source: PLOS Glob Public Health. 2022 Feb 9;2(2):e0000176. doi: 10.1371/journal.pgph.0000176 (PMC10021309; doi:10.1371/journal.pgph.0000176)
Supplement: S1 Table — (DOCX) [file pgph.0000176.s001.docx]

# Supplementary Table 1:

### Mitigation Measures and Estimated Impacts on Coverage of Essential Health Interventions as well as COVID Transmission Risk

Examples of mitigation measures deployed by countries with estimated effectiveness data

|  | **Impact on Infection Risk** | | | | | | | | | **Impact on Coverage** | | |  | |
| --- | --- | --- | --- | --- | --- | --- | --- | --- | --- | --- | --- | --- | --- | --- |
| **Mitigation Measure** | **Reduction in Number of Visits per Person per Intervention** | | | | **Reduction in Disease Transmission Risk in the remaining visits** | | | | | **Increase in Coverage compared to current Coverage Level** | | | **Applicable to:** | |
| Hygiene and Distancing Measures | | | | | | | | | | | | | | |
| Social distancing at health facilities | AFG | 0% | | This has no impact on the average number of visits per person per intervention, but it means that fewer people will be able to be accommodated at health facilities due to the need to keep people socially distanced; consider additional mitigation measures that might make it possible to see fewer people at the HF (e.g., increasing community outreach, etc.). | | AFG | | 5% | Keeping people socially distanced by at least one meter will reduce the spread of COVID-19 | AFG | 2% | Coverage may increase a small amount as people may feel that health facilities that are not crowded are therefore safer. | | All services |
|  | Iraq | 0% | |  |  | Iraq | | 10% |  | Iraq | 2% |  |  |  |
|  | Morocco | 0% | |  |  | Morocco | | 10% |  | Morocco | 1-2% |  |  |  |
|  | Suggested | 0% | |  |  | Suggested | | 10% |  | Suggested | 2% |  |  |  |
| Temperature screening of patients and health faculty staff prior to entry into the health facility | Iraq | 0% | | This has no impact on the average number of visits per person per intervention | | Iraq | 5% | | May make visits safer by screening out patients with fever who may have COVID-19 – and directing them to appropriate care, which will reduce the chance that other patients will be exposed. However, the impact is limited as people are contagious before showing symptoms or may be asymptomatic. | Iraq | 2% | Might increase coverage for essential health services as the public may have more trust regarding the safety of healthcare services during the epidemic. | | All services |
|  | Pakistan | 0% | |  |  | Pakistan | 2% | |  | Pakistan | 2% |  |  |  |
|  | Morocco | 0% | |  |  | Morocco | 0% | |  | Morocco | ? |  |  |  |
|  | Somalia | 0% | |  |  | Somalia | 0% | |  | Somalia | 0% |  |  |  |
|  | Suggested | 0% | |  |  | Suggested | 2% | |  | Suggested | 2% |  |  |  |
| Provision of surgical masks to be worn by health workers in health facilities (without education) | Iraq | 0% | | This has no impact on the average number of visits per person per intervention | | Iraq | 10% | | The use of surgical masks by health workers will reduce the spread of COVID-19 | Iraq | 2% | A strict protocol for surgical mask use amongst staff at health facilities might increase confidence in the population that health facilities are safe to visit. | | All services |
|  | AFG | 0% | |  |  | AFG | | 5% |  | AFG | 2% |  |  |  |
|  | Pak | 0% | |  |  | Pak | | 10% |  | Pak | 2% |  |  |  |
|  | Morocco | 0% | |  |  | Morocco | | 15% |  | Morocco | 1-2% |  |  |  |
|  | Somalia | 0% | |  |  | Somalia | | 15% |  | Somalia | 1-2% |  |  |  |
|  | Suggested | 0% | |  |  | Suggested | | 15% |  | Suggested | 2% |  |  |  |
| Provision of surgical masks to be worn by health workers in health facilities with education on correct use masks (clean masks, donning, doffing etc.) | Suggested | 0% | | This has no impact on the average number of visits per person per intervention | | Suggested | | 30% | The use of surgical masks by health workers will reduce disease transmission. Education to health workers on the importance of using a clean mask and how to put on/take off the mask etc. will improve effectiveness of the mask mitigation measure | Suggested | 2% | A strict protocol for surgical mask use amongst staff at health facilities might increase confidence in the population that health facilities are safe to visit. | | All services |
| Implement a requirement that patients bring their own masks (cotton, store bought) to the health facility and wear them. | Suggested | 0% | | This has no impact on the average number of visits per person per intervention | | Suggested | | 10% | The use of any face covering may have a impact on infection transmission | Suggested | 2% | People may feel less likely to spread or catch COVID-19 if they are wearing a face covering. This may give them more confidence that it is safe to visit a health facility. | | All services |
| Distribution of surgical masks to patients who are visiting HFs along with instruction on how to wear them correctly | Suggested | 0% | | No impact on average number of visits per person per intervention | | Suggested | | 30% | Use of surgical masks by patients will reduce the spread of COVID-19 transmission between patients in HFs and from patient to HWs | Suggested | 2% | Use of masks by patients and health workers may inspire confidence that the health facility is taking measures to reduce the spread of COVID-19 and consequently increase coverage | | All services |
| Access to handwashing facilities / use of hand sanitizer at all health facilities for staff and clients | AFG | 0% | |  | | AFG | | 5% | Hand washing will reduce viral transmission by reducing hand to mouth/nose /eye transmission and will increase awareness of the need for a clean environment to reduce the spread of COVID-19 | AFG | 0% | A strict hygiene protocol related to handwashing might ensure confidence in population that health facilities are safe to visit | | All services |
|  | Pak | 0% | |  |  | Pak | | 10% |  | Pak | 2% |  |  |  |
|  | Morocco | 0% | |  |  | Morocco | | 20% |  | Morocco | 1% |  |  |  |
|  | Somalia | 0% | |  |  | Somalia | | 20% |  | Somalia | 1% |  |  |  |
|  | Suggested | 0% | |  |  | Suggested | | 5% |  | Suggested | 2% |  |  |  |
| Sanitize frequently touched surfaces | Morocco | 0% | | This has no impact on the average number of visits per person per intervention | | Morocco | | 20% | There is limited evidence related to the spread of COVID-19 on surfaces; however, surface transmission may happen in a small number of cases. | Morocco | 1% | Increased cleanliness of health facilities may increase confidence within the population that health facilities are clean and safe to visit. | | All services |
|  | Suggested | 0% | |  |  | Suggested | | 1-2% |  | Suggested | 1-2% |  |  |  |
| Ensure all ambulances have PPE supplies (masks, hand sanitizer etc.) | Iraq | 0% | | This has no impact on the average number of visits per person per intervention | | Iraq | | 5% | For patients who are transferred by ambulance, use of PPE in the ambulance may reduce transmission of COVID 19. This mitigation strategy will only apply to patients who are transferred by ambulance | Iraq | 5% | If community members know that ambulances are equipped with the necessary PPE to prevent the spread of COVD-19, it might increase their confidence in the health system and its capacity to deliver safe health care during the pandemic | | EmOC Interventions |
|  | Suggested | 0% | |  |  | Suggested | | 0.5% |  | Suggested | 1-2% |  |  |  |
| Ventilation of treatment rooms (fan, open windows) | AFG | 0% | | This has no impact on the average number of visits per person per intervention | | AFG | | 3% | COVID-19 is spread through droplet and aerosol transmission. Aerosol transmission is particularly worrisome in indoor, crowded and inadequately ventilated spaces. Improving ventilation (fan, windows open) can decrease transmission of COVID-19. | AFG | 0% | There may be a slight increase in coverage if the public is made aware that increased ventilation can decrease the spread of COVID-19 and if the population knows that health facilities are implementing this measure, which will improve perceptions of the safety of health facilities | | All services |
|  | Morocco | 0% | |  |  | Morocco | | 20% |  | Morocco | 1%-2% |  |  |  |
|  | Suggested | 0% | |  |  | Suggested | | 5-10% |  | Suggested | 0-2% |  |  |  |
| Establish an isolation area within health facilities for suspected and positive cases of COVID-19 | AFG | 0% | | This has no impact on the average number of visits per person per intervention | | AFG | | 2% | Isolating people who have or are suspected to have COVID-19 may reduce the spread of COVID-19 to other people who are using the health facility. However, people who are suspected of having COVID-19 and are directed to the isolation unit but are later found to not have COVID-19 may be unnecessarily exposed | AFG | 0% | Isolation areas will not increase coverage | |  |
|  | Suggested | 0% | |  |  | Suggested | | 2-5% |  | Suggested | 0% |  |  |  |
| Reduced in Number of Health Facility Visits | | | | | | | | | | | | | | |
| Provide a 3-month supply of FP commodities to WRA (i.e., Pill) | Morocco | 50% | | Reduces number of visits to 1 per quarter from monthly visits (4 visits in a year compared to 12) | | Morocco | | 0% | Risk for remaining visits will remain the same | Morocco | ? | Providing women, a 3-month supply of FP commodities may maintain coverage although adherence may be better if they do not have to come to get it as often. | | FP - Pills |
|  | Suggested | 67% | |  |  | Suggested | | 0% |  | Suggested | 2% |  |  |  |
| Encourage more women to use long-lasting FP methods (i.e., injection, IUD)  Scheduling client visits | Iraq | 0% | | Women who use long-lasting FP methods will require fewer visits to a health facility to refill supplies | | Iraq | | 10% | Risk for remaining visits will remain the same | Iraq | 0% | More women may be encouraged to use long-lasting FP methods, which may increase FP coverage | |  |
|  | Suggested | 50% | |  |  | Suggested | | 0% |  | Suggested | 2% |  |  |  |
| Shifting health care to community providers/tele-medicine | | | | | | | | | | | | | | |
| Expansion of outreach or mobile health teams (CHWs, LHWs, mobile teams) | AFG | | 5-10% | Outreach services will reduce the number of people who must attend a HF, especially for mild illnesses. The average number of visits per intervention could decrease as a % of care provided outside the HF is increased. The % decrease in visits to HFs may be impacted by the scope of services offered by outreach or mobile health teams | | AFG | | 2-5% | Risk for remaining visits will remain the same | AFG | 5% | Increased provision of services at the community level may increase coverage as people who may not have attended a HF may access services in the community. Outreach/mobile health services may reach some people who may not have gone to a HF at all due to distance time or other prohibitive factors beyond factors associated with COVID-19. But could also just maintain coverage (instead of going to HF, women/children get care at community level). | | FP, Breastfeeding, Vaccination, Child health  ANC  *Services impacted by this mitigation strategy will be dependent on the scope of outreach/mobile services offered by different countries. |
|  | Iraq | | 10% |  |  | Iraq | | 0% |  | Iraq | 0% |  |  |  |
|  | Pakistan | | 0% |  |  | Pakistan | | 0% |  | Pakistan | 10% |  |  |  |
|  | Suggested | | 5-10% |  |  | Suggested | | 0% |  | Suggested | 2-5% |  |  |  |
| Implementation of e-health (electronic remote health services/telemedicine) | AFG | 5% | | Consultations provided by mobile telephone or other e-health platforms might make it possible for some women to not have to come to the clinic for every required consultation. | | AFG | 0% | | Risk for remaining visits will remain the same | AFG | 0% | M-health or e-health may make services accessible to more people, and may increase coverage, but could also just maintain coverage (instead of going to HF, women get care though remote sessions). | | All services |
|  | Iraq | 10% | |  |  | Iraq | 0% | |  | Iraq | 0% |  |  |  |
|  | Pakistan | 10% | |  |  | Pakistan | 0% | |  | Pakistan | 10% |  |  |  |
|  | Suggested | 5-10% | |  |  | Suggested | 0% | |  | Suggested | 2% |  |  |  |
| Capacity (training and availability of health care providers) | | | | | | | | | | | | | | |
| Training of service providers on methods for reducing transmission of COVID-19 | AFG | | 0% | This has no impact on the average number of visits per person per intervention | | AFG | | 4% | Health care workers might be more knowledgeable about how to limit transmission of COVID-19  Availability of more qualified staff will adherence to policy/procedures, which could reduce transmission of COVID-19. | AFG | 2% | Better trained staff and staff with better knowledge about how to reduce transmission of COVID may increase community confidence in health services. | | All services |
|  | Iraq | | 0% |  |  | Iraq | | 0 -2% |  | Iraq | 5% |  |  |  |
|  | Pakistan | | 0% |  |  | Pakistan | | 0% |  | Pakistan | 2% |  |  |  |
|  | Suggested | | 0% |  |  | Suggested | | 1-2% |  | Suggested | 2% |  |  |  |
| Ensure availability of health providers at health facilities (i.e., policies in place to not task shift essential MNCH HF staff to Covid-19 duties, including contract tracing) | Iraq | | 0% | This has no impact on the average number of visits per person per intervention | | Iraq | | 0% | Risk for remaining visits will remain the same | Iraq | 5% | Availability of skilled MCH health providers at primary health facilities will bolster confidence in availability of quality health services at PHCCs.  Impact might depend on how much of the staff shortage is alleviated. | | All services |
|  | Pakistan | | 0% |  |  | Pakistan | | 2% |  | Pakistan | 5% |  |  |  |
|  | Morocco | | 0% |  |  | Morocco | | 0% |  | Morocco | 5% |  |  |  |
|  | Somalia | | 0% |  |  | Somalia | | 0% |  | Somalia | 5% |  |  |  |
|  | Suggested | | 0% |  |  | Suggested | | 0% |  | Suggested | 1-5% |  |  |  |
| Strengthening of Supply Chain | | | | | | | | | | | | | | |
| Ensuring sustainable supply of PPE**^[[1]](#footnote-1)^**  *Do NOT USE THIS MITIGATION STRATEGY IF ALREADY USING MASK MITIGATION STRATEGY/IES | AFG | | 0% | This has no impact on the average number of visits per person per intervention | | AFG | | 5% | PPE can reduce the spread of COVID-19, but the impact depends on the type of PPE used (i.e., masks) and if HWs are trained to use it correctly. | AFG | 0% | If people are confident that health facilities will have a constant supply of PPE, they may feel more comfortable visiting a health facility | | All services |
|  | Iraq | 0% | |  |  | Iraq | | 10% |  | Iraq | 2% |  |  |  |
|  | Suggested | 0% | |  |  | Suggested | | 5-85% |  | Suggested | 2% |  |  |  |
| Maintain or increase the supply of essential health commodities at all health facilities | AFG | | 0% | This has no impact on the average number of visits per person per intervention | | AFG | | 0% | Risk for remaining visits will remain the same | AFG | 4-5% | Consistent and reliable supply of health commodities might increase coverage. If availability of commodities is already consistent, then little or no increase in coverage should be expected. If availability of commodities improves and becomes reliable, coverage may increase. | | ANC  Family Planning  Child health |
|  | Iraq | | 0% |  |  | Iraq | | 0% |  | Iraq | 10% |  |  |  |
|  | Suggested | | 0% |  |  | Suggested | | 0% |  | Suggested | 0-5% |  |  |  |
| Change in Procedures for Service Provision | | | | | | | | | | | | | | |
| Limit the number of accompanying persons for delivery | AFG | 0% | | No difference as the model already assumes just the woman and a HW in the delivery room | | AFG | 3% | | No difference as the model already assumes just the women and a HW in the delivery room. | AFG | 0% | Could lead to reduction in coverage: If women know that they will not be permitted to have a family member/ support person accompany her for delivery, she may decide to not deliver at a health facility | | Delivery, Newborn Care |
|  | Suggested | 0% | |  |  | Suggested | 0% | |  | Range | 0% |  |  |  |
|  | Suggested | 0% | |  |  | Suggested | 0-0.5% | |  | Suggested | 0% |  |  |  |
| Guidelines and messaging | | | | | | | | | | | | | | |
| Media campaigns to reduce COVID-19 through information that increases knowledge of how to reduce spread. | AFG | | 0% | This has no impact on the average number of visits per person per intervention | | AFG | | 5% | Health care workers and communities will be more knowledgeable about how to limit transmission of COVID-19 | AFG | 5% | May increase community knowledge related to COVID-19 and increase confidence that health facilities can be visited safely. | | All services |
|  | Iraq | | 0% |  |  | Iraq | | 0% |  | Iraq | 5% |  |  |  |
|  | Pakistan | | 0% |  |  | Pakistan | | 0% |  | Pakistan | 0% |  |  |  |
|  | Suggested | | 0% |  |  | Suggested | | 0% |  | Suggested | 1-2% |  |  |  |
| Media campaigns to tell people that it is safe to visit the health facility | Suggested | | 0% | This has no impact on the average number of visits per person per intervention | | Suggested | | 0% | Suggested | Range | 1-2% | Messaging that tells people that HFs are safe to visit, may encourage people to feel safe about coming to the HF for their regular health care needs | | All services |
| Messaging to stress the importance of seeking care for specific interventions, such as health facility delivery – even during COVID-19 | Suggested | | 0% | This has no impact on the average number of visits per person per intervention | | Suggested | | 0% | No impact. | Suggested | 1-2% | Messaging may increase public education about the benefits of seeking care from a health care facility, including during COVID-19 | | All services |
| Job aids, algorithms, protocols and flow charts displayed in HFs in the context of COVID-19 | AFG | | 0% | This has no impact on the average number of visits per person per intervention | | AFG | | 3-5% | Health care workers will be more knowledgeable and have references related to how to limit transmission of COVID-19 | AFG | 0% | Strict hygiene protocols might inspire confidence in the population that health facilities are safe to visit. | | All services |
|  | Pakistan | | 0% |  |  | Pakistan | | 5% |  | Pakistan | 2% |  |  |  |
|  | Suggested | | 0% |  |  | Suggested | | 1-5% |  | Suggested | 1-2% |  |  |  |
| General Interventions | | | | | | | | | | | | | | |
| Increase COVID-19 testing capacity | AFG | 0% | | This has no impact on the average number of visits per person per intervention | | AFG | | 0% | No impact on remaining HF visits | AFG | 2% | May increase confidence in the health care system | | All services |
|  | Suggested |  |  |  |  | Suggested | | 0% |  | Suggested | 1-2% |  |  |  |
| Provide financial support (i.e., cash transfer scheme/relief package) | AFG | 0% | | This has no impact on the average number of visits per person per intervention | | AFG | | 0% | No impact on remaining HF Visits | AFG | 5% | Financial support may encourage/enable some people to access maternal services | | All services |
|  | Pakistan | 0% | |  |  | Pakistan | | 0% |  | Pakistan | 2% |  |  |  |
|  | Suggested | 0% | |  |  | Suggested | | 0% |  | Suggested | 1-2% |  |  |  |
| Strengthen referral system | AFG* | 0% | | This has no impact on the average number of visits per person per intervention | | AFG | | 5% | No impact on remaining HF visits | AFG | 2% | A strengthened referral system may increase public confidence in the health system | | Delivery, Newborn care |
|  | Suggested |  |  |  |  | Suggested | | 0% |  | Suggested | 1-2% |  |  |  |
| Strengthen local supervision | Iraq | 0% | | This has no impact on the average number of visits per person per intervention | | Iraq | | 5% | No impact on remaining HF visits | Iraq | 5% | Supportive supervision strengthens the health system and enables health workers to offer quality services and improve performance, which may improve community confidence in services offered by the health system | | All services |
|  | Suggested | 0% | |  |  | Suggested | | 0% |  | Suggested | 1-2% |  |  |  |

*For AFG, this included PPE In ambulances – which was moved to Hygiene section above

1. If “Use of Masks in Public places/Distribution of masks to people coming to the HF” and/or hand sanitizer is included as a hygiene mitigation measure, do not also include this mitigation measure (Ensure sustainable supply of PPE) to avoid doubling of mitigation measures. [↑](#footnote-ref-1)
