# Supplementary material for: Benefit-risk analysis of maintaining essential Reproductive, Maternal, Newborn, and Child Health (RMNCH) services against risk of COVID-19 infection
Source: PLOS Glob Public Health. 2022 Feb 9;2(2):e0000176. doi: 10.1371/journal.pgph.0000176 (PMC10021309; doi:10.1371/journal.pgph.0000176)
Supplement: S1 Text — (DOCX) [file pgph.0000176.s003.docx]

Risk-Benefit Analysis
of Maintaining Coverage of Essential Health Interventions during the COVID Pandemic

USER GUIDELINES

Risk-Benefit Analysis Model Instructions

| **Steps:**   1. **Determine interventions/intervention packages to include** 2. **Establish original coverage** 3. **Figure out impact of COVID on coverage of different interventions/intervention packages** 4. **Figure out mitigation strategies** 5. **Estimate impact of mitigation strategies on coverage** and (potentially) effectiveness of interventions, also added risk of COVID transmission 6. **Enter coverage and effectiveness data into LiST** to calculate number of lives saved through mitigation measures 7. **Run COVID risk analysis** in Excel to estimate number of lives lost in maintaining coverage of essential services 8. **Calculate benefit-risk ratios (lives saved by maintaining services divided by lives lost due to COVID)** |
| --- |

Note: The Excel model is used in conjunction with the Famplan and LiST module that are part of the Spectrum model.

The version used here is version 6.06 available for download here:
**https://avenirhealth.org/software-spectrum.php**


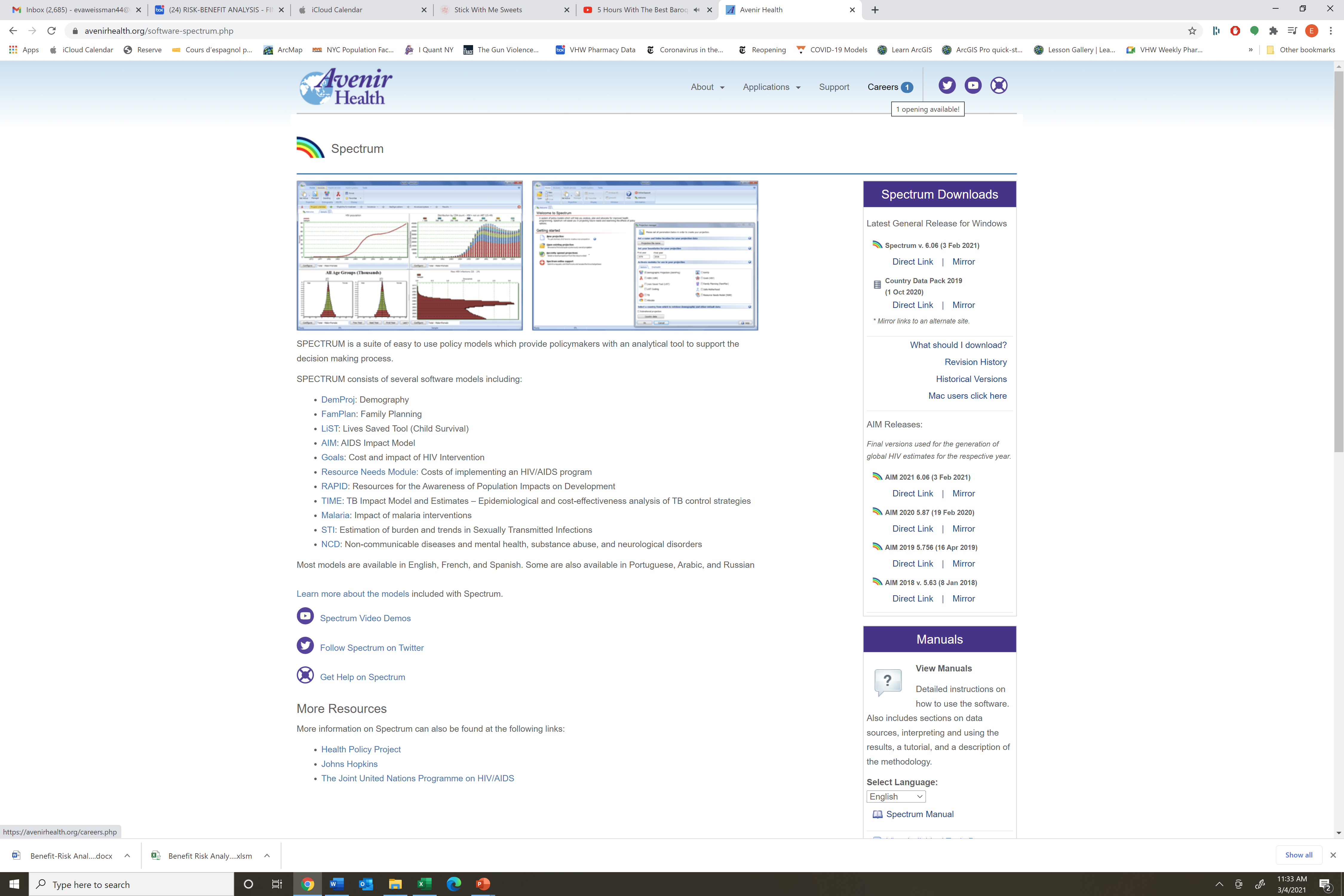


For more information on Spectrum and LiST, see here:
<https://www.avenirhealth.org/software-spectrum.php>

1. **Intro Sheet**

Select country from drop-down list, enter username and date.

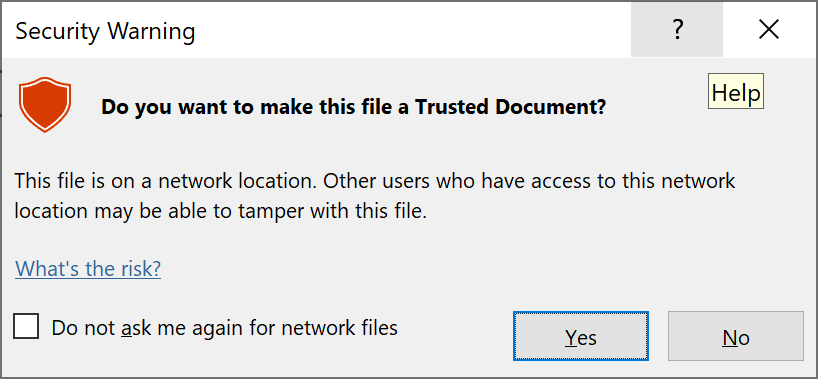
This file contains macros to facilitate certain tasks. When the file is opened, Excel might display a security warning saying that macros have been disabled. To make sure the model works as intended, click Enable Content and make the document a Trusted Document.

For more info, see <https://powerspreadsheets.com/how-to-enable-macros-excel/>

**Note: Throughout the model, light green cells mark cells with user input, all other cells contain descriptions or formulas that should not be changed.**

1. **Select RMNCAH Services to be included in the Analysis**

On Sheet **1. Select Services**, select packages to be included in the risk-benefit analysis by placing an x next to the package.

The packages available are:

In the second step, press button A to specify which sub-interventions to include (again by placing an x next to the sub-intervention.

Finally, press button B. This will update the entire model so it will only show the interventions and sub-interventions selected. In the example below, the model would only show the first six family planning methods, but not male sterilization and traditional methods.

***Note: Annex 1 at the end of this document contains definitions of the different interventions included in LiST and this model***

1. **Setting Up Spectrum/LiST Model and Downloading Country Baseline Data**

The country baseline data (both demographic and epidemiological as well as baseline coverage data) will come from the Spectrum model. The user should open the Spectrum tool and either open an existing country model or generate one, if none is available.


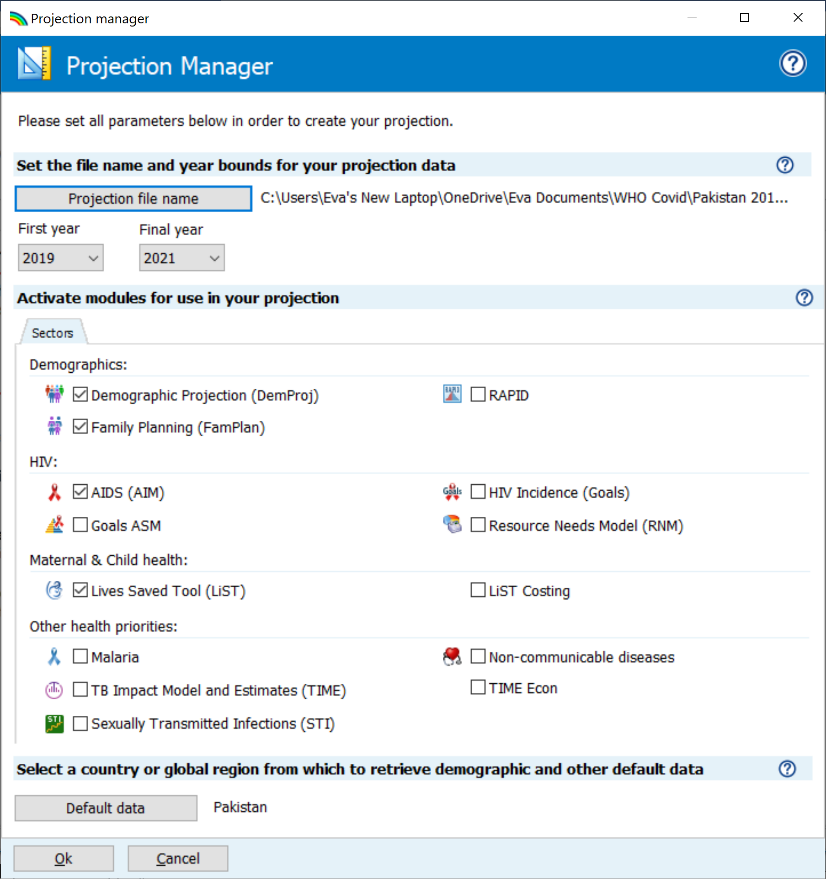


If no recent application exists, click on the Spectrum icon, and select “New Projection”, which will bring up the Projection Manager. For an existing projection, select the file, then click on the Project Manager icon.

Set years to 2019 to 2021 for a two-year projection.

Then select the following modules: DemProj, FamPlan, and the Lives Saved Tool (LiST).

At the bottom, click “Default data” and select the country of choice to load the required demographic and epidemiological data.

In a final step, go to the top to the “Projection file name” button and save the file with the country’s name and data. Click OK.

In the next step, configure the LiST module. For both existing and new country models, in the main dialog box, click on the word “Modules”, then select the LiST icon from the list of icons at the top.


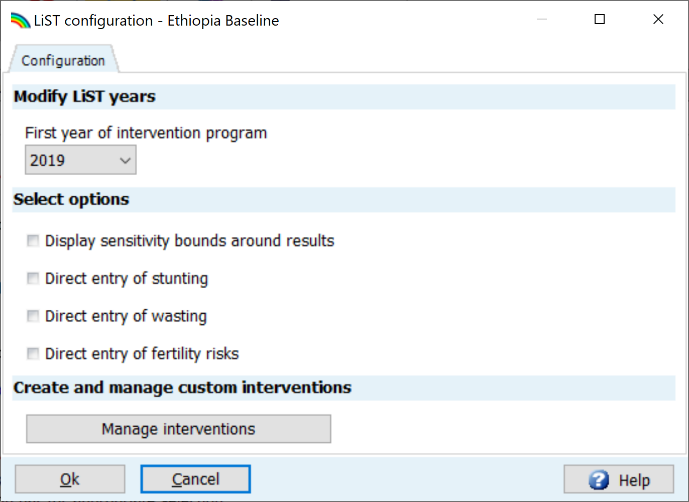

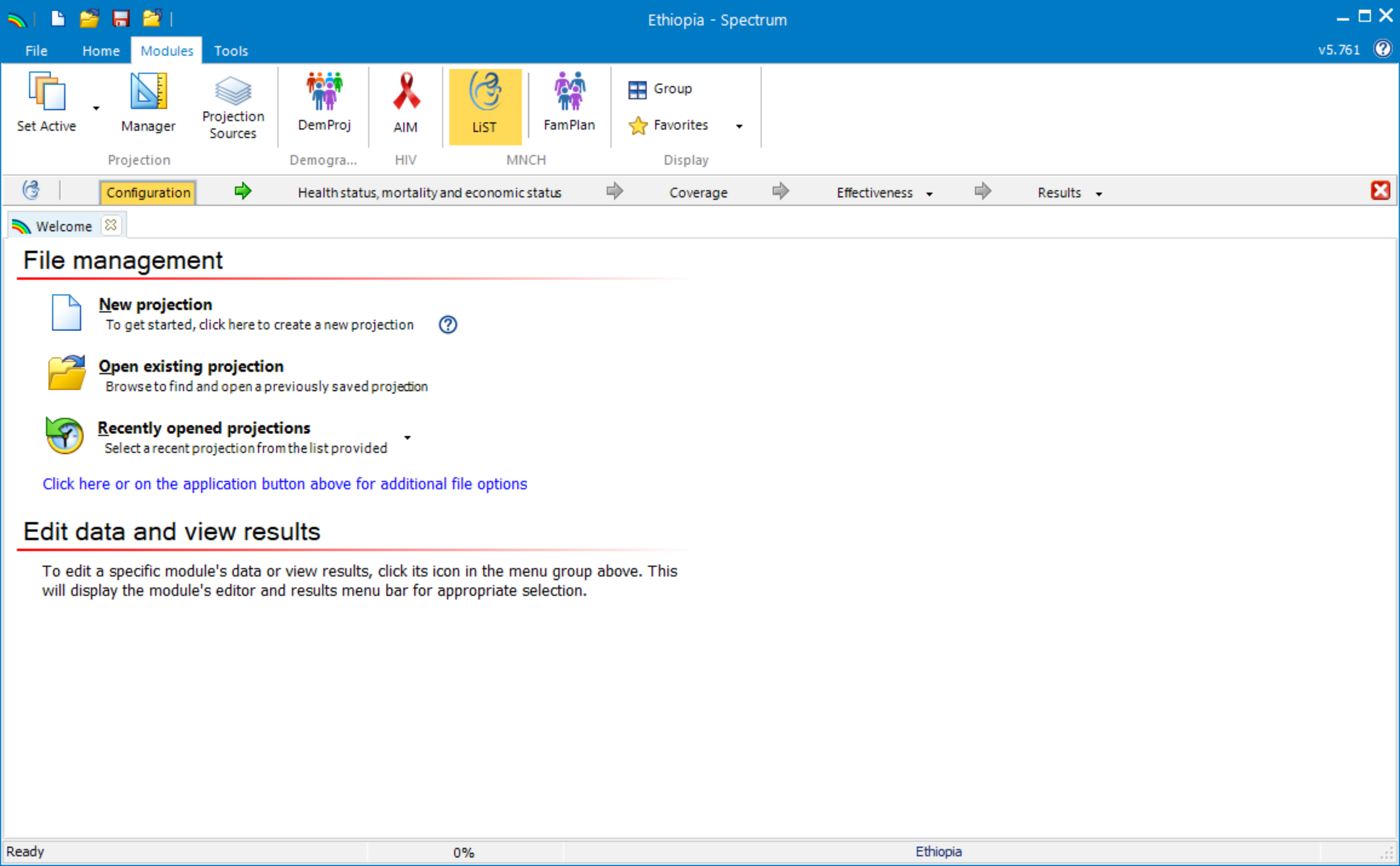


Go to Configuration. Select 2019 as “First year of intervention program.”

Click OK, then close the country model.


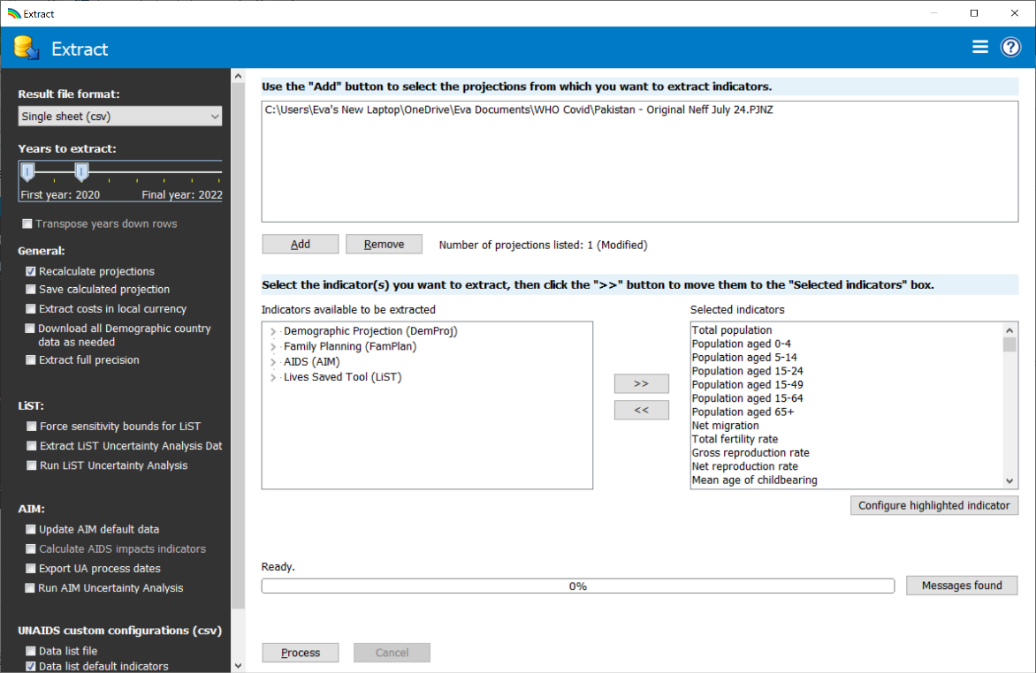

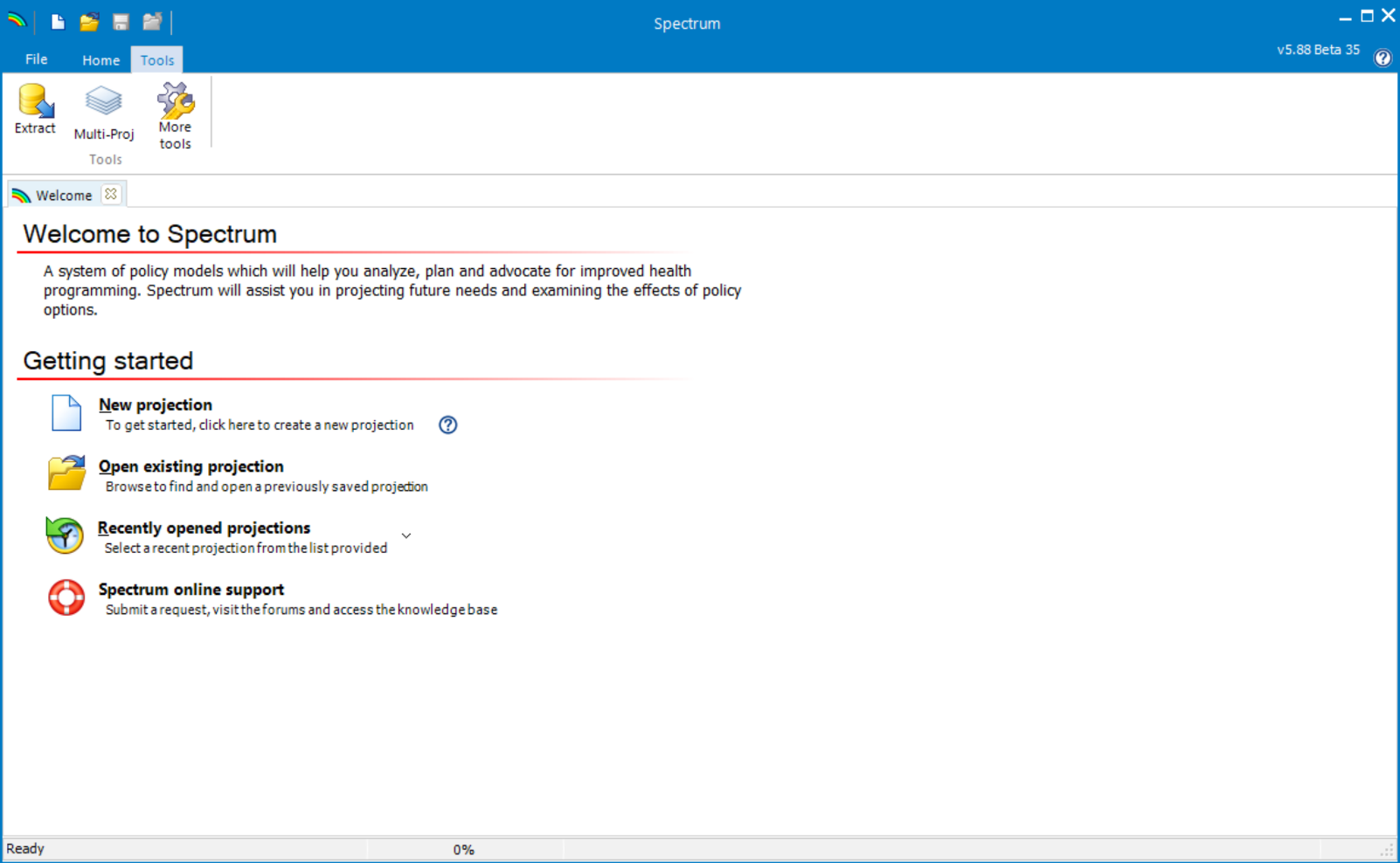
To extract the data from the model, go to the Extract tool and open the model again from there. Go to the Tool tab, then select the Extract icon.

Perform the following steps:

- Set "Years to Extract" to 2019 to 2021.
- Press "Add" button and select country file.
- Select DemProj, Famplan and LiST and press **>>** button to move all indicators to the Selected Indicators table.
- For “Result File format” Choose: Single sheet (csv).
- Hit Process.

In a final step, select Columns A-J from the file created by Spectrum and paste into cell A1 on the sheet **Spectrum Baseline Data** of the Excel model.


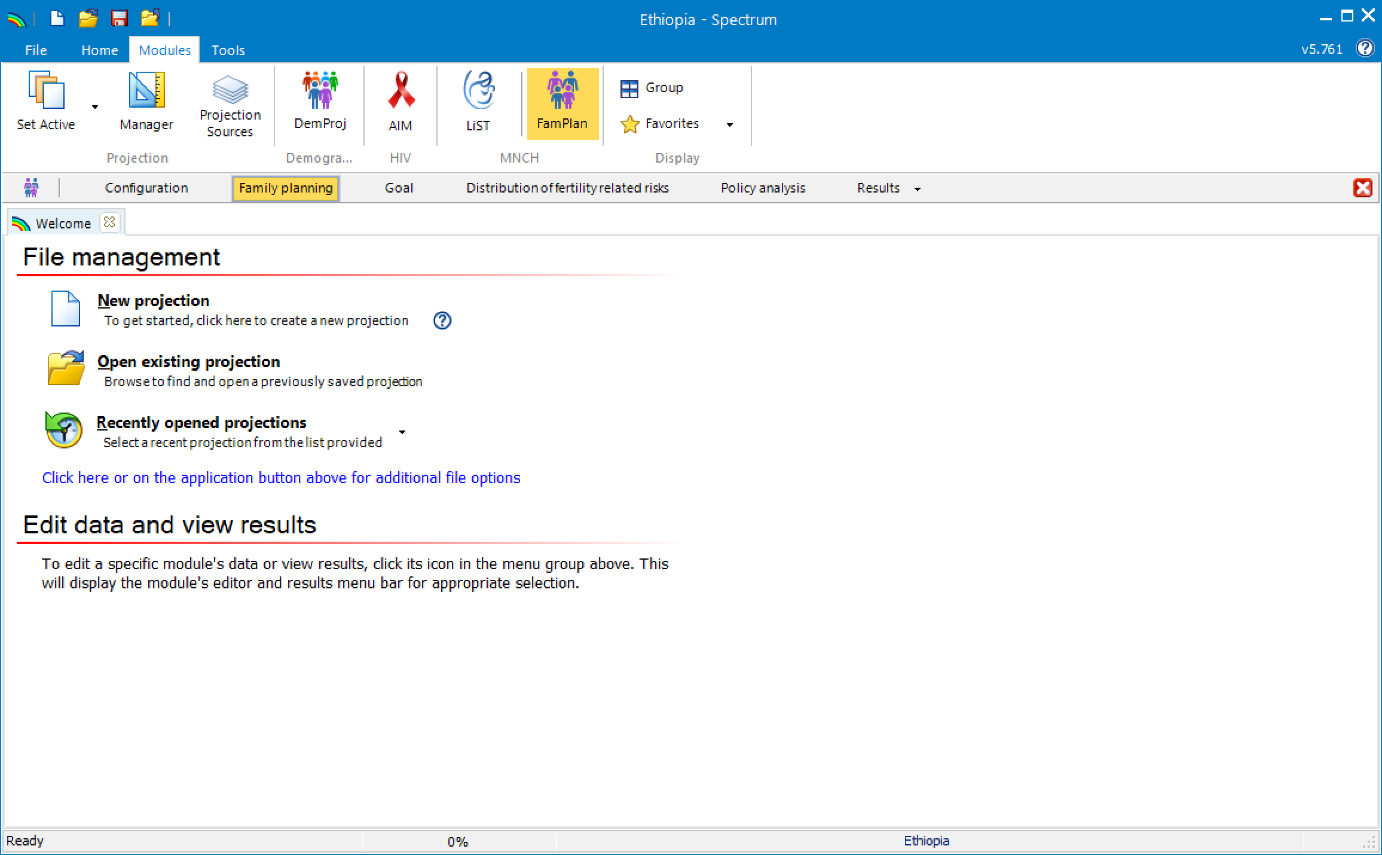
Technical Note: For some reason, the FP data is not available in Spectrum's Extract. Until this issue is fixed, the data needs to be copied directly from FamPlan model tables. Go to the FamPlan module, Goal tab and click inside the table. Then right click and select "Copy All" from the drop-down menu. Paste into cell O5 of the Spectrum Baseline Data sheet. Repeat that process with the data in the Method Mix table. Paste into cell O10.

1. **Goal Tab - CPR**


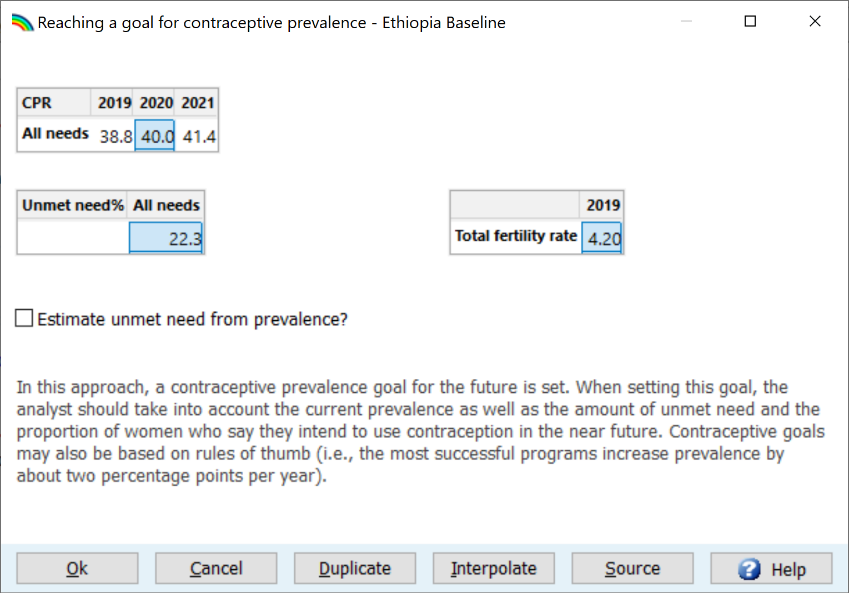


1. **Family Planning Tab – Method Mix**


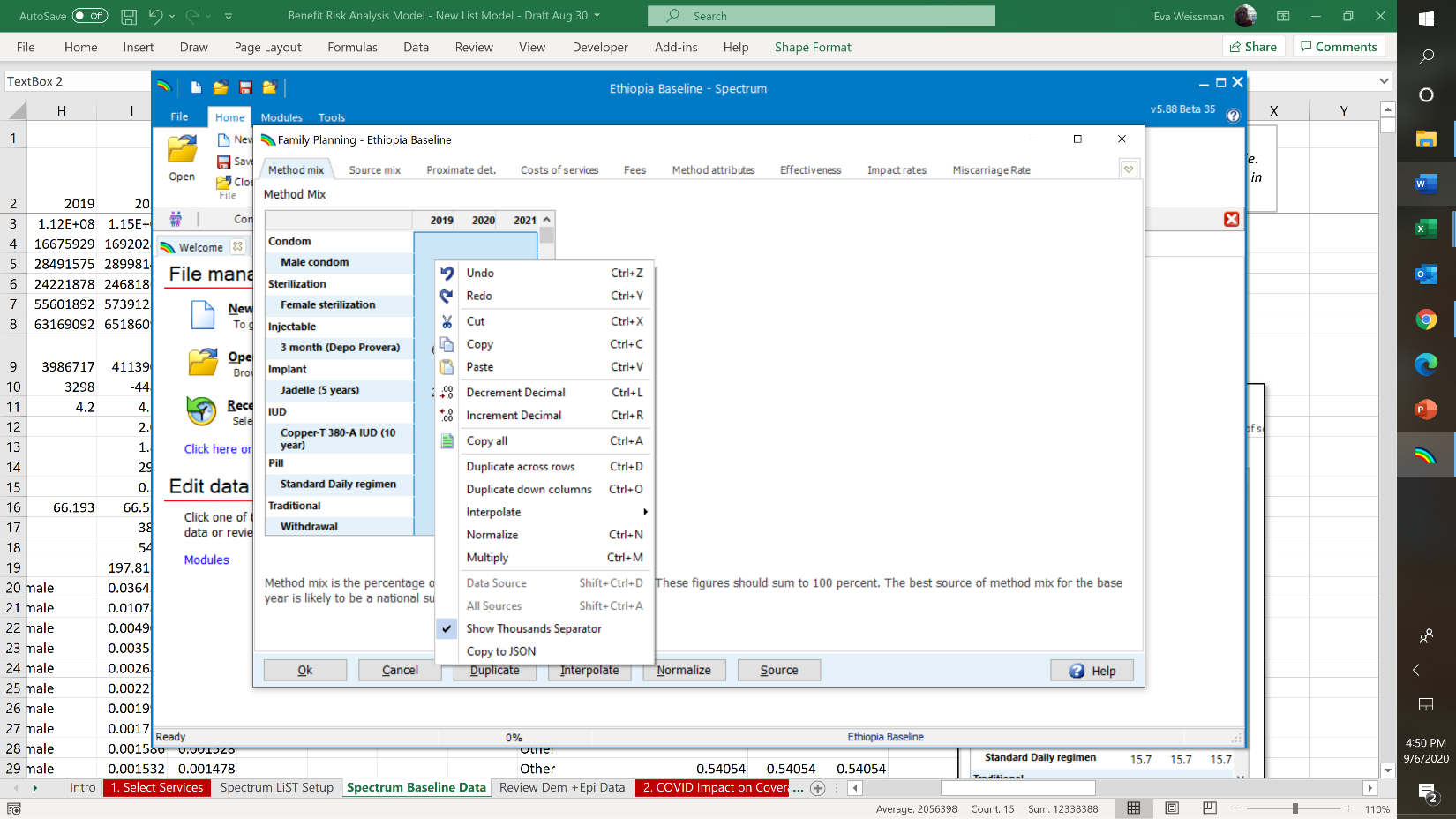

1. **Review Demographic and Epidemiological Data**

The next sheet, **Review Dem and Epi Data** shows the demographic and epidemiological inputs required. Most of them come from the Spectrum model data extract, from both the DemProj and the LiST module. Some disease prevalence and incidence data are not provided by Spectrum, the Excel model in those cases provides data from WHO and other sources.

The user should do a quick review of these assumptions.

Note: If the user wants to use different population or disease data that come from the Spectrum model, the changes need also to be made to the Spectrum model. (Column K shows the respective tables in those modules in which that changed data would have to be entered. More detailed instructions will be provided).

Data sources can be found in the Spectrum tool by clicking on the Projection Sources icon.


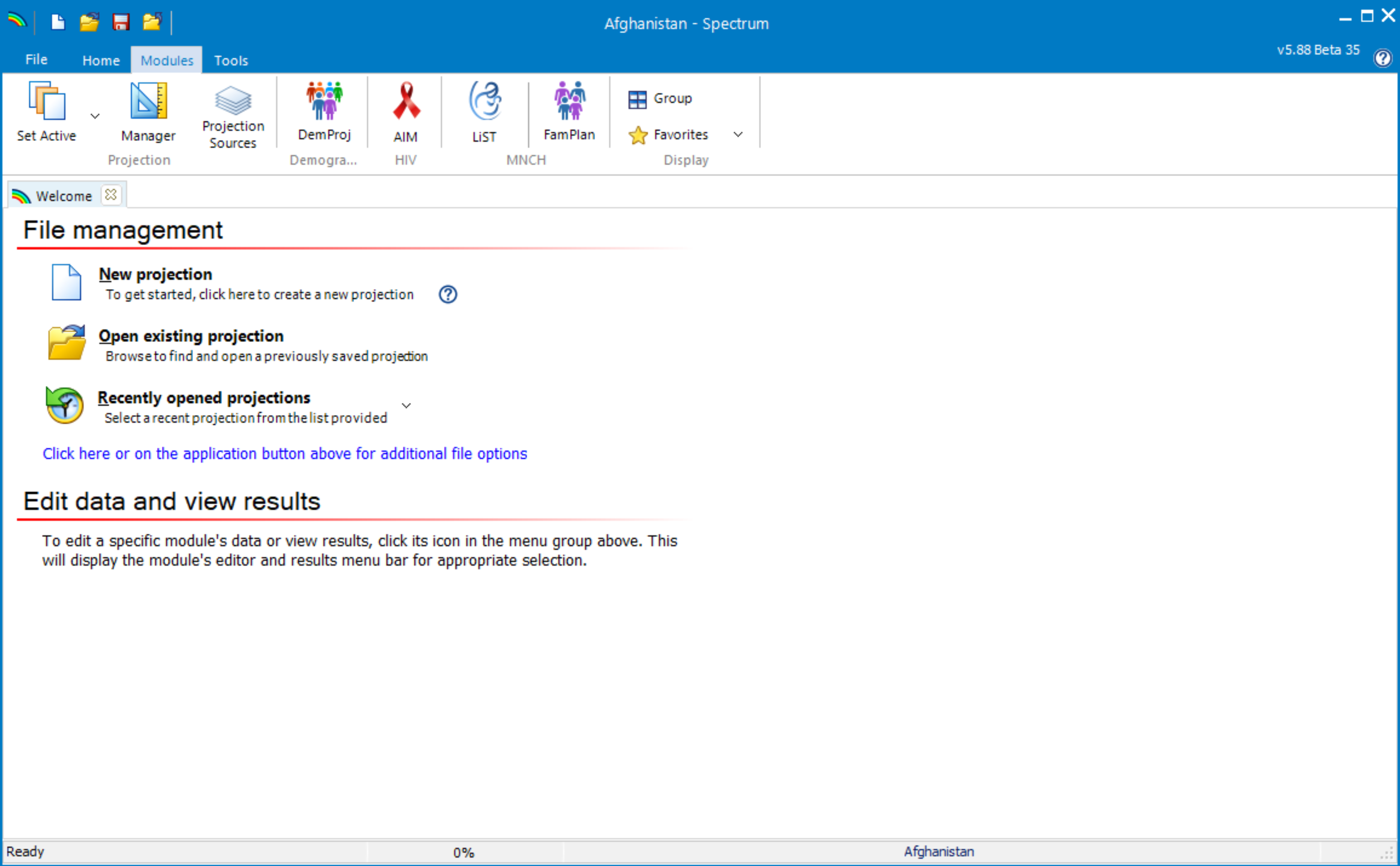


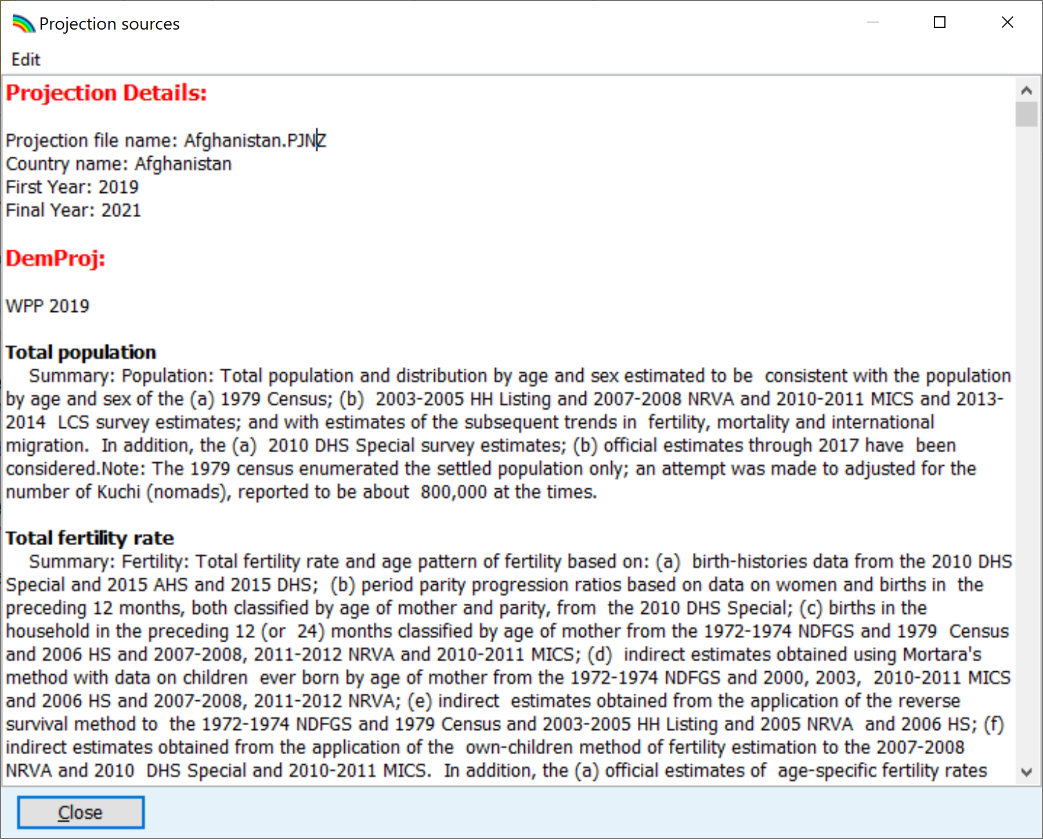


1. **Estimate COVID’s Impact on Coverage**

On sheet **2. COVID Impact on Coverage** the user should enter percentage decrease in coverage due to the COVID pandemic. This can either be done by package (the specified decrease in coverage will be applied to all sub-interventions) or individually for each sub-intervention. Select the preferred method from the drop-down box, then press the orange button to show the selected table.

**Option1:**

**Option 2:**

Enter the percentage in the light green cells next to the package or sub-intervention name.

1. **Choose Mitigation Strategies and Estimate Impact on Coverage**

On Sheet **4. Mitigation Impact on Coverage** the user should enter different Mitigation Strategies as well as

1. how these measures would affect the number of contacts with the health facility
2. the impact these strategies are expected to have on coverage (% relative improvement beyond general recovery in coverage expected for 2021), and
3. how these measures would affect infection/transmission risk during any contact with a health worker during remaining visits

In columns F-BX, the country should enter which specific interventions are affected by the measures (by marking those interventions with an x)

**Example 1**

| **Modifications to ensure continued/safe delivery of services** | **Estimated % decrease in number of health facility visits** | **% Decrease in Infection/ Transmission Risk in Remaining Visits** | **Estimated Relative Increase in Coverage** | **Family planning** | | | | | | | | | | **Antenatal Care** | | | | |
| --- | --- | --- | --- | --- | --- | --- | --- | --- | --- | --- | --- | --- | --- | --- | --- | --- | --- | --- |
|  |  |  |  | **FP - Pills** | **FP - Condoms** | **FP – injectables** | **FP - Implants** | **FP - IUD** | **FP – Female Sterilization** | **FP – Male Sterilization** | **FP – Traditional Methods** | **Antenatal care (at least 1 visit** | **Antenatal care (at least 4 visits** | **TT – Tetanus toxoid vaccination** | **IPTp – Intermittent preventative treatment of malaria during pregnancy** | **Syphilis detection and treatment** | **Calcium supplantation** | **Iron and folate supplementation** |
| **General** |  |  |  |  |  |  |  |  |  |  |  |  |  |  |  |  |  |  |
| Double the supply of PPE given to each facility | **0%** | **20%** | **2%** | **x** | **x** | **x** | **x** | **x** | **x** | **x** | **x** | **x** | **x** | **x** | **x** | **x** | **x** | **x** |

Explanation:

This measure does not have any impact on the number of visits to the health facility required for any of the interventions. It is estimated, though, to lead to a relative increase in 2021 coverage by 2% over the 2021 coverage expected without any mitigation measures (as it might increase confidence of the population that all is being done to decrease their risk of infection when seeking care. The main effect of this strategy would be the decrease in the transmission risk between patients and health care workers during visits to the facility.

Regarding the affected interventions, this mitigation strategy is expected to affect all interventions.

**Example 2**

| **Modifications to ensure continued/safe delivery of services** | **Estimated % decrease in number of health facility visits** | **% Decrease in Infection/ Transmission Risk in Remaining Visits** | **Estimated Relative Increase in Coverage** | **FP - Pills** | **FP - Condoms** | **FP - Injectables** | **FP - Implants** | **FP - IUD** | **FP - Female Sterilization** | **FP - Male Sterilization** | **FP - Traditional Methods** |
| --- | --- | --- | --- | --- | --- | --- | --- | --- | --- | --- | --- |
| **Family Planning** |  |  |  |  |  |  |  |  |  |  |  |
| Give women 3 cycles of contraceptive pills per visit instead of just 1. | **67%** | **0%** | **10%** | **x** |  |  |  |  |  |  |  |

Explanation:

This measure is expected to reduce the number of visits a woman has to make to the health facility to get her pill supplies by 2/3, i.e., 67% (only 4 visits per year instead of 12 visits per year). This intervention does nothing to decrease transmission risk during the remining contacts with the health facility.

It is estimated to lead to a relative increase of 2% over the 2021 coverage expected without any mitigation measures (if, for instance, the 2021 coverage is expected to be 40%, the coverage with the mitigation strategies is expected to go to 40% + 10%*40% = 44%) as it might encourage care-seeking among women who weren’t willing to come to the facility when their resupply visit was monthly.

Regarding the affected interventions, this mitigation strategy applies only for contraceptive pills.

Steps:

For each Mitigation Strategy, enter the impact it will have on coverage and how it will affect the number of visits required and the virus transmission risk for those visits (Columns A-D). Also put an x or all interventions that will be affected by the mitigation measure. By default, the mitigation measures in each subsection are set to cover all the interventions in that package, but this can be changed (as in the family planning examples show, where the mitigation measure only affects contraceptive pill use not any other family planning methods.

On the far right end of the sheet, all the impacts of the different mitigation measures will automatically be added up to give a total impact by intervention (no user impact required).

Please refer to Annex 1 for guides to mitigation measures and estimated impacts.

*WHO. March 2020. COVID-19: Operational guidance for maintaining essential health services during an outbreak. WHO/2019-nCoV/essential_health_services/2020.2*

1. **Coverage Summary sheet**

The next sheet, **5. Coverage Review,** gives a summary of the four coverage rates:

1. Original coverage rates pre-COVID (year 2019)
2. Coverage rates with COVID (year 2020)
3. Coverage WITHOUT (additional) mitigation measures (year 2021)
4. Coverage rates WITH (additional) mitigation measures (year 2021)

No user input is required.

Review especially columns H and I. To avoid overly optimistic projections regarding the resurgence of coverage rates in 2021, the model has two built-in caps – a) no coverage in 2021 can exceed 100%, and b) it can also not exceed 102% of the 2019 baseline coverage. Column V shows the values before application of the cap and highlights values to which that cap was applied. To change the assumptions underlying the values on this sheet, go back to sheet 3. Coverage WITHOUT mitigation or sheet 4. Mitigation Impact on Coverage.

*Technical Note:*

*The number of women and children shown as receiving interventions on this sheet are for illustrative purposes only. Since the numbers of pregnancies and births in this table are based on family planning use assumptions made in the original, pre-COVID Spectrum country model they might slightly differ from the actual numbers. The final result tables (Sheets 6 - 10) will reflect the user-defined FP use assumptions*

1. **Estimation of Lives Saved through Mitigation Measures in LiST**

The new coverage data (WITHOUT and WITH mitigation measures) need to be entered into the Spectrum model, specifically into the FamPlan and the LiST modules, to estimate how many lives will be lost due to the disruption in coverage because of the pandemic and how many lives could be saved through mitigation measures.

This will require the creation of two new country model versions:

a) one with the coverage expected in 2021 WITHOUT any mitigation measures

1. one with the coverage expected in 2021 WITH mitigation measures

The following sheets set up the data for easy entry into the Spectrum/FamPlan/LiST modules.

Getting estimates for the number of lives lost and saved involves the following steps:

1. Open original country model, save as "Country Model 2019-2021 WITHOUT mitigation"

2. Enter inputs in FamPlan and LiST modules

3. Paste results from LiST module into Excel sheet "LiST Output WITHOUT M" (instructions provided on that sheet).

4. Save and close LiST model.

5. Again open original model and save this time as "Country Model 2019-2021 WITH Mitigation."

6. Enter inputs into FamPlan and LiST and paste results back into Excel model.

7. Save and close LiST model.

Continue to Sheet **6. Lives Lost Due to Disruption** in this Excel model.

**Entry into FamPlan**

The table on sheet **Input Into FamPlan WITHOUT Mitigation** sets up the data for easy entry into the FamPlan module.

Open original country model and save as a different scenario: "Country Model 2019-2021 WITHOUT mitigation"

Then copy and paste the last three columns of the Total CPR table into the FamPlan, Goal tab (in the example below, select 38.7, 31.0 and 34.9 and then select the three number cells in the CPTR table and hit Paste.

Then copy the two last columns in the Method Mix section into the Method Mix table.

Specifically, the original 2020 Coverage column in FamPlan/LiST will be replaced with the “2020 Coverage WITH COVID” and the 2021 Coverage column will be replaced with the “Coverage in 2021 WITHOUT Mitigation”.


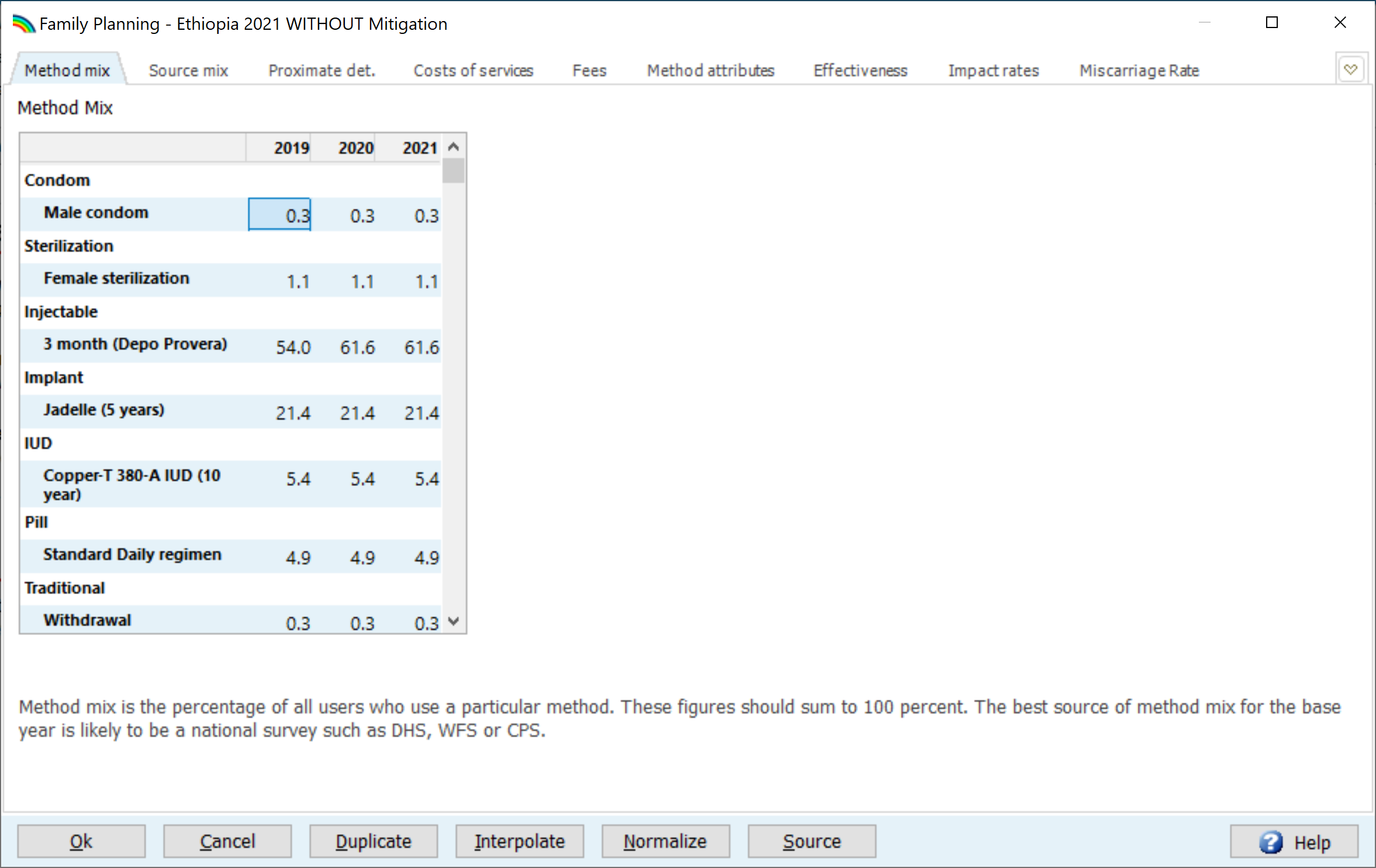

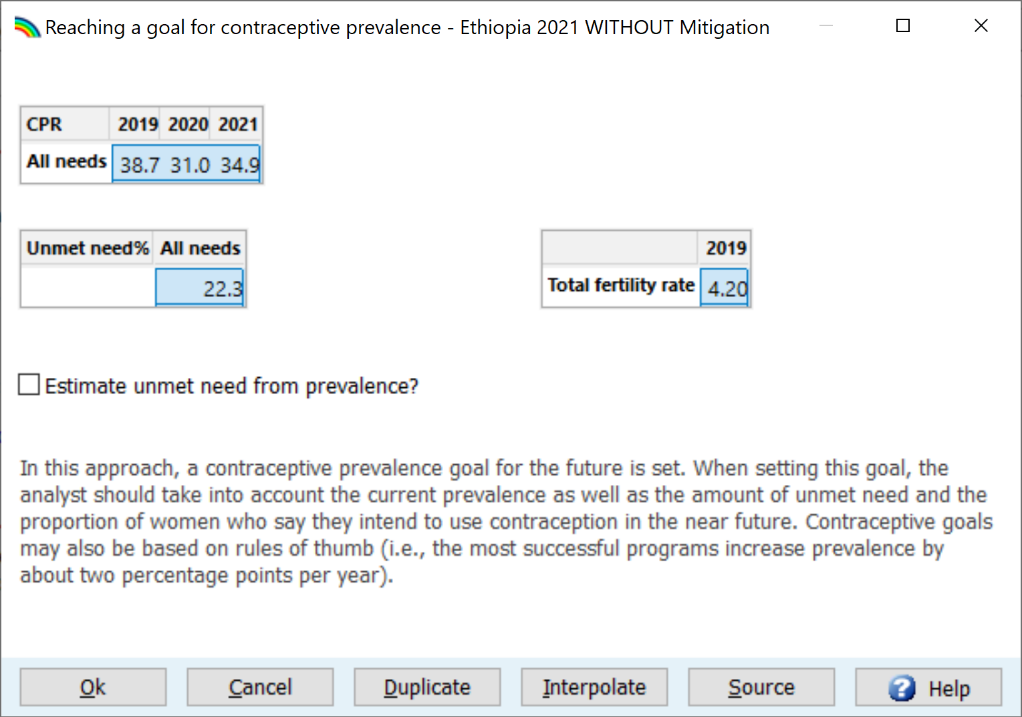


**Important: Make sure to press the OK button after the data is entered (otherwise the data will just revert to the original data) and Save the scenario.**

**Entry into LiST Module**

The table on sheet **Input Into LiST WITHOUT Mitigation** sets up the data for easy entry into the LiST module.

Copy and Paste the last three columns into the different LiST Coverage tables (there are seven of them as shown in the screenshot below).


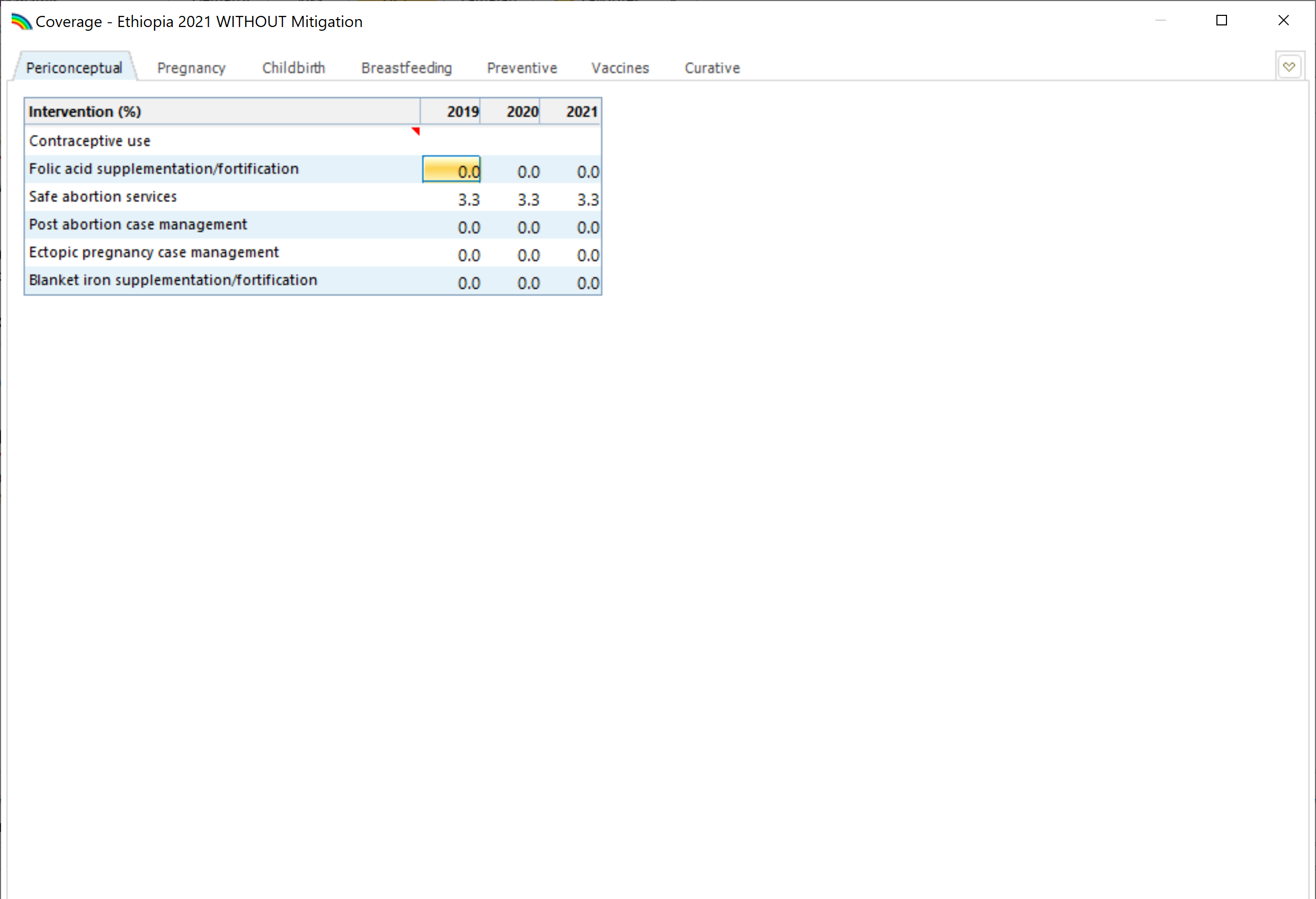


For Antenatal care interventions there are two different settings in LiST. To enter data for the first two lines (Antenatal Care (at least 1 visits) and Antenatal Care (at least 4 visits), select the “Utilization and Quality” radio button.

For the other interventions (those provided during antenatal care, select the Coverage button before entering the data.

| **With Utilization and Quality button selected**  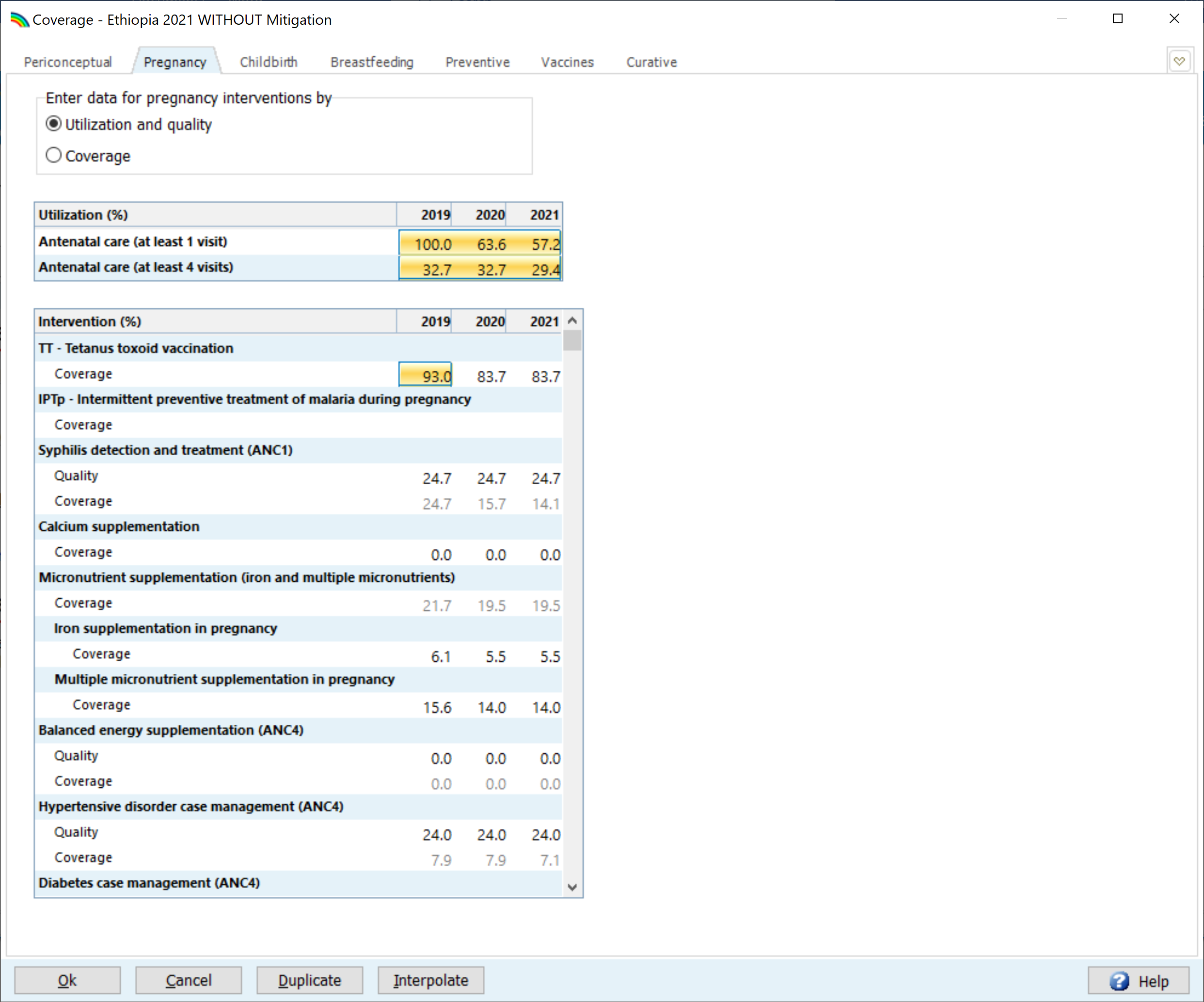 | **With Coverage button selected (enter data for all but the antenatal care coverage rates in this table)**  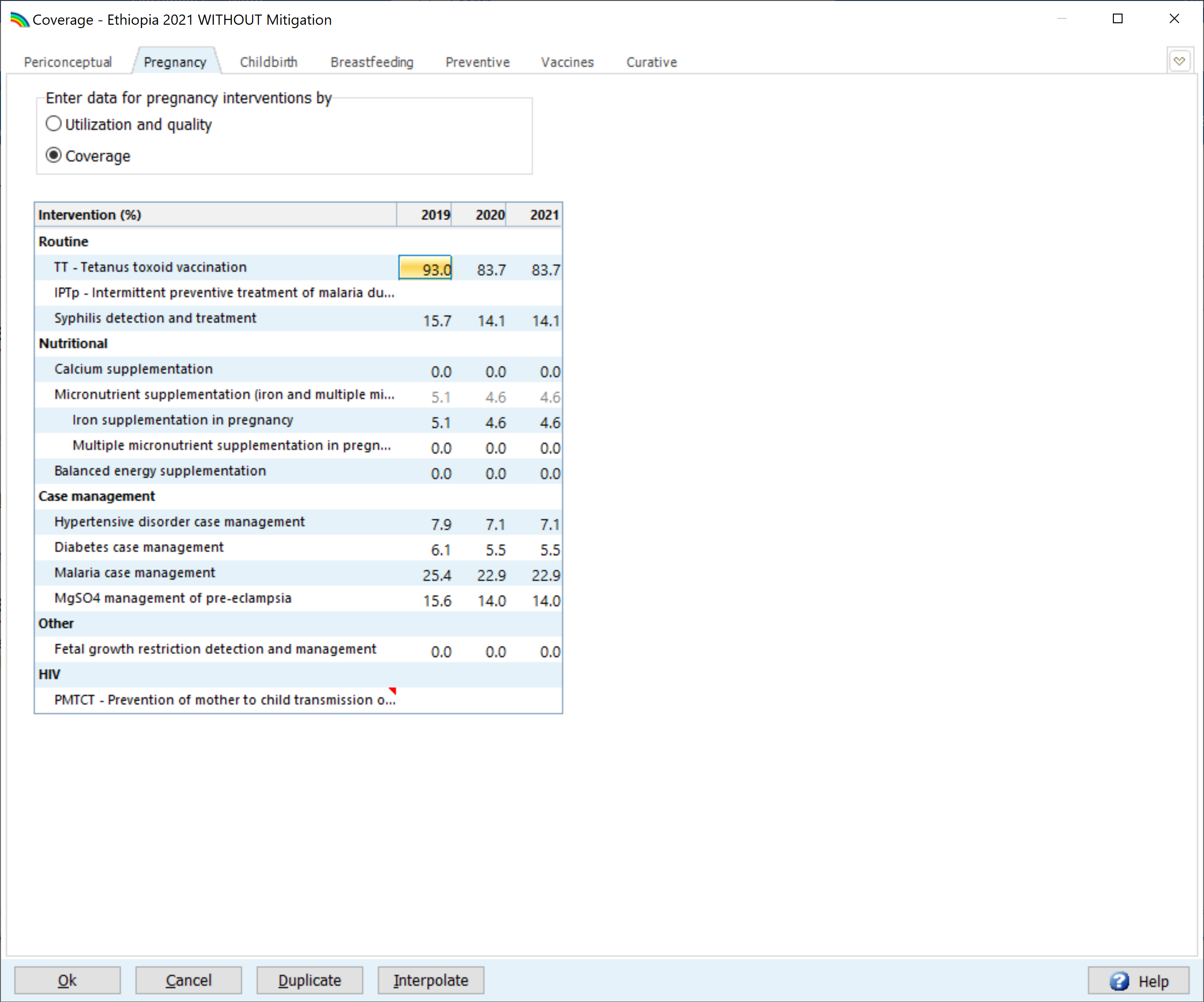 |
| --- | --- |
| 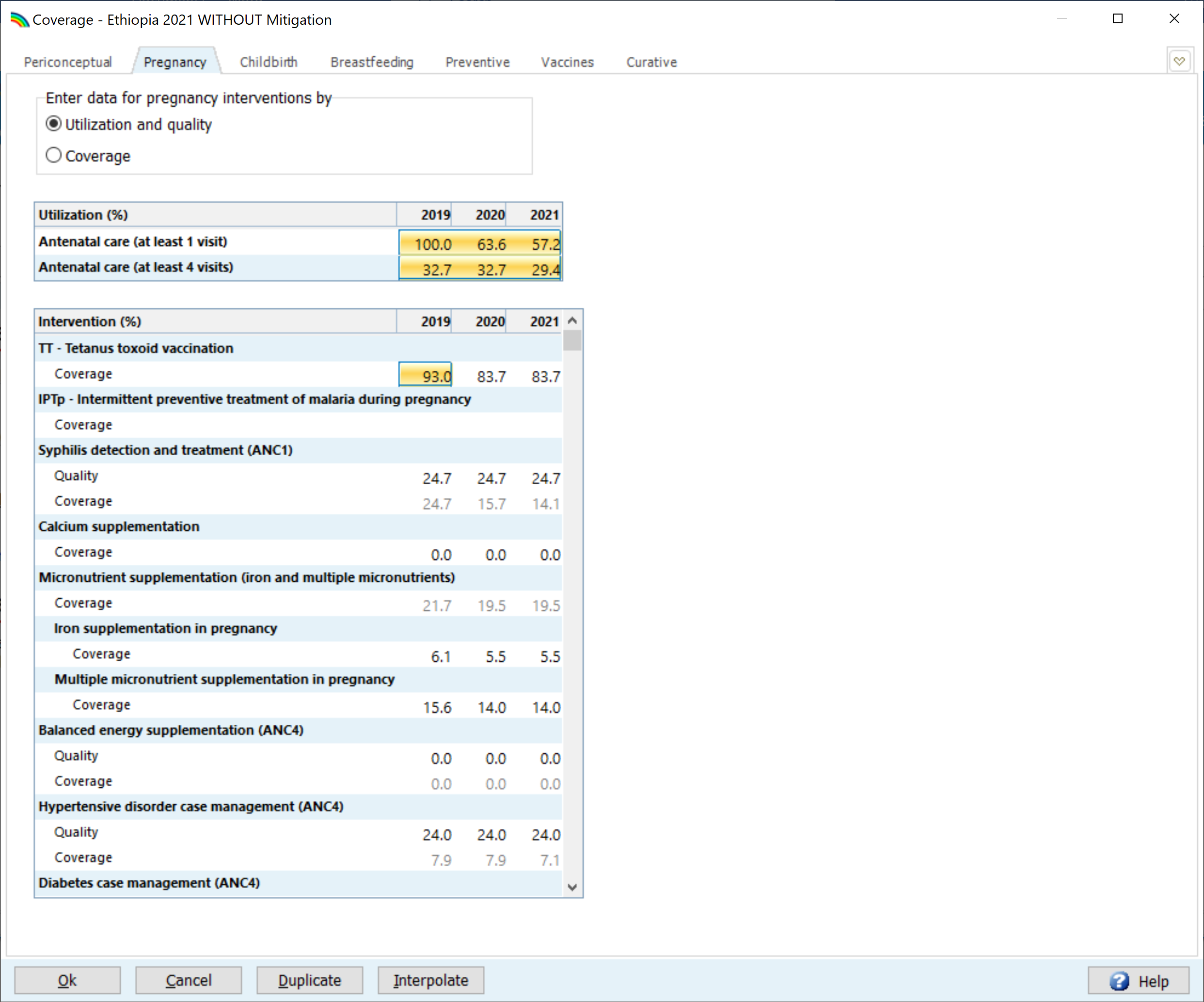 | 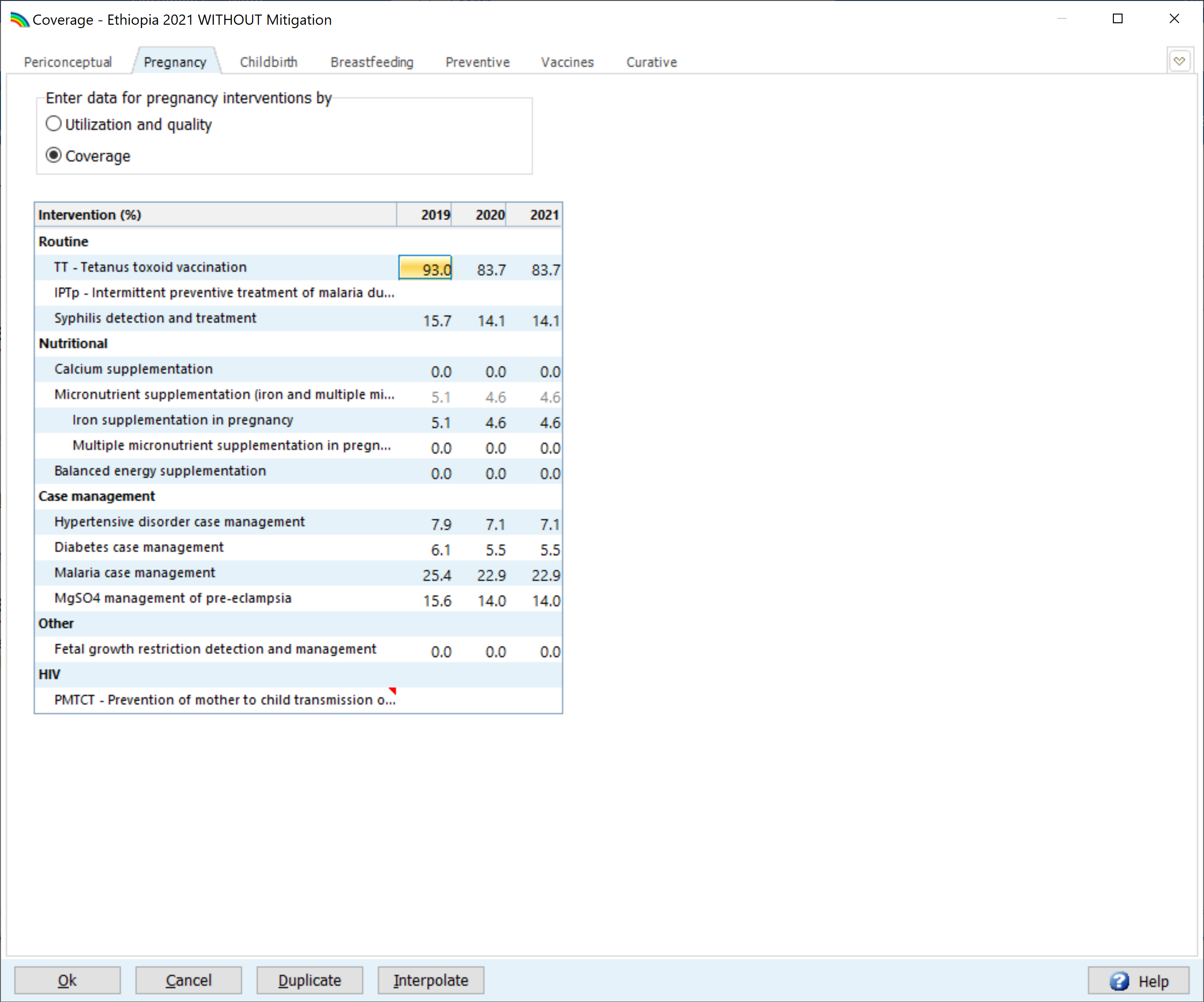 |

**Important: Again, make sure to press the OK button below each table after entering the data (otherwise the data will just revert to the original data when moving to the next entry table) and Save the scenario.**

The Childbirth/delivery interventions tab follows the same structure (Health Facility Delivery is entered in a separate table). All the other tabs only show coverage and do not have a separate “Utilization and Quality” table. See below.


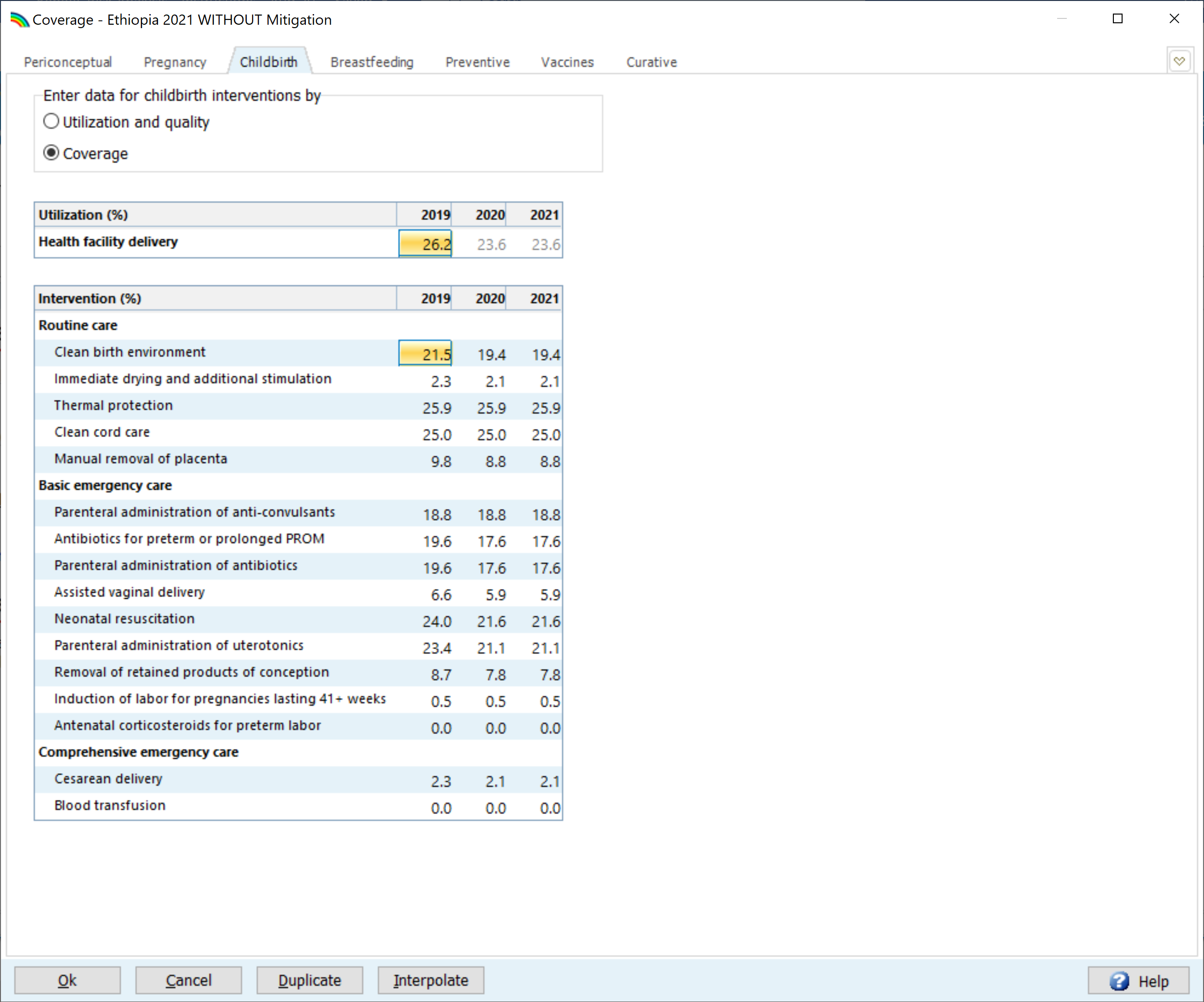

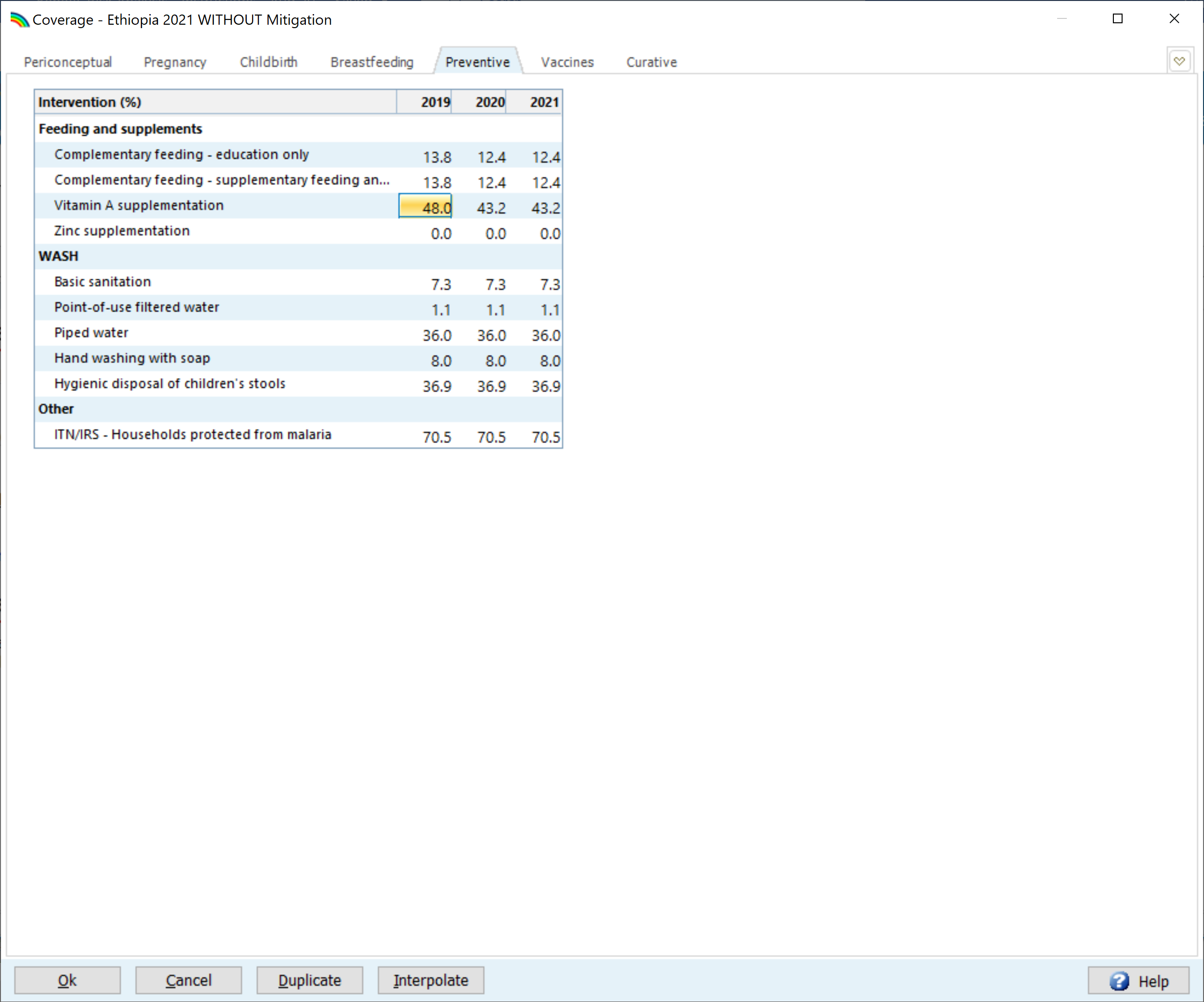


Breastfeeding needs to be entered separately for the four age categories (<1 month, 1-5 months, 6-11 months, and 12-23 months. Select “Prevalence” to get the right input table to paste the data into.

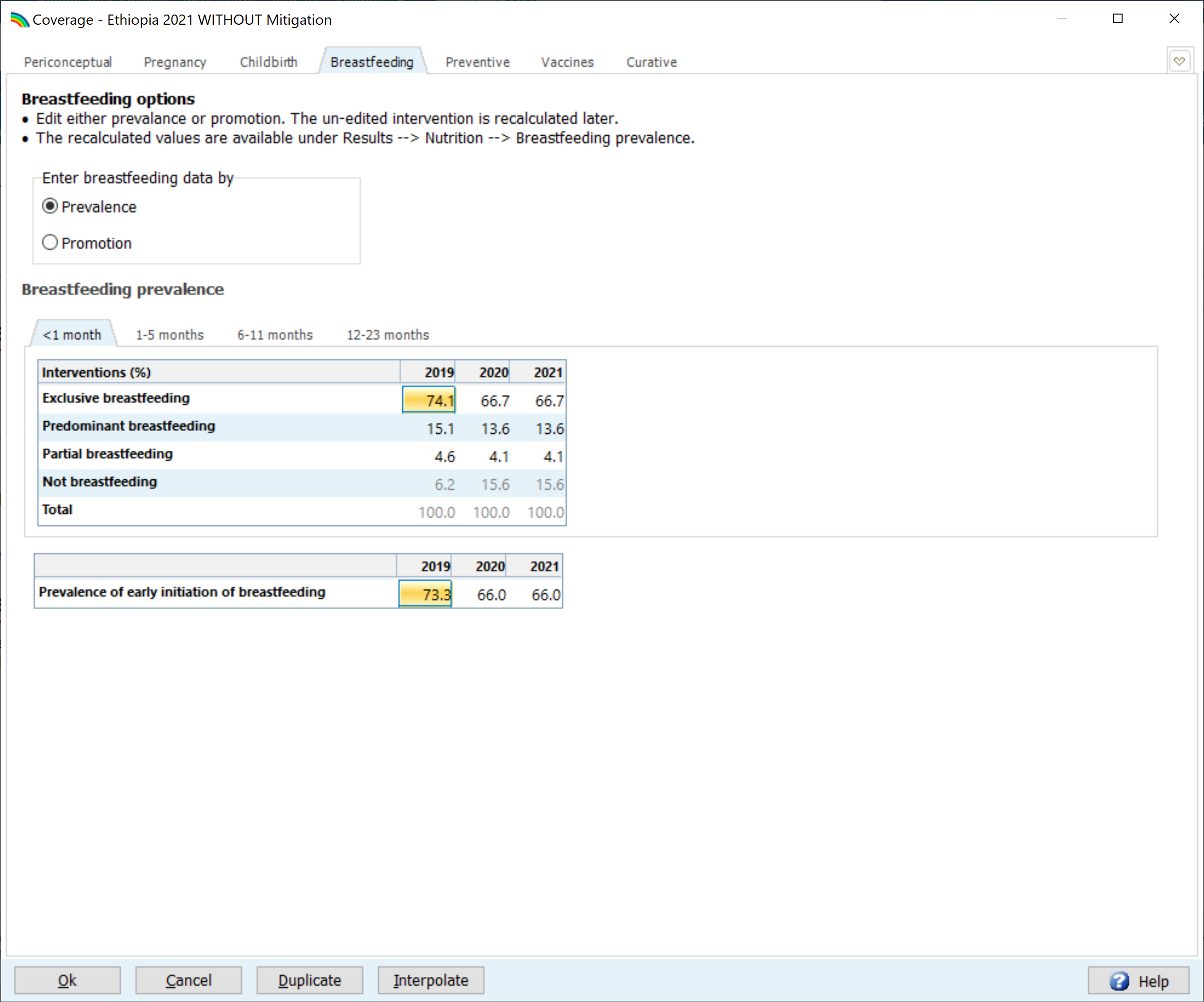


**Pasting Results from LiSt into Excel model**

The sheet **LiST Outputs** collates the result of the LiST calculations of lives saved. In the LiST module, go to Results, then to “Additional neonatal lives saved by intervention. “


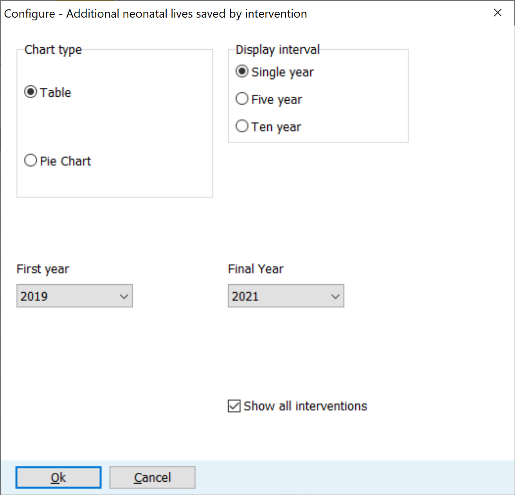

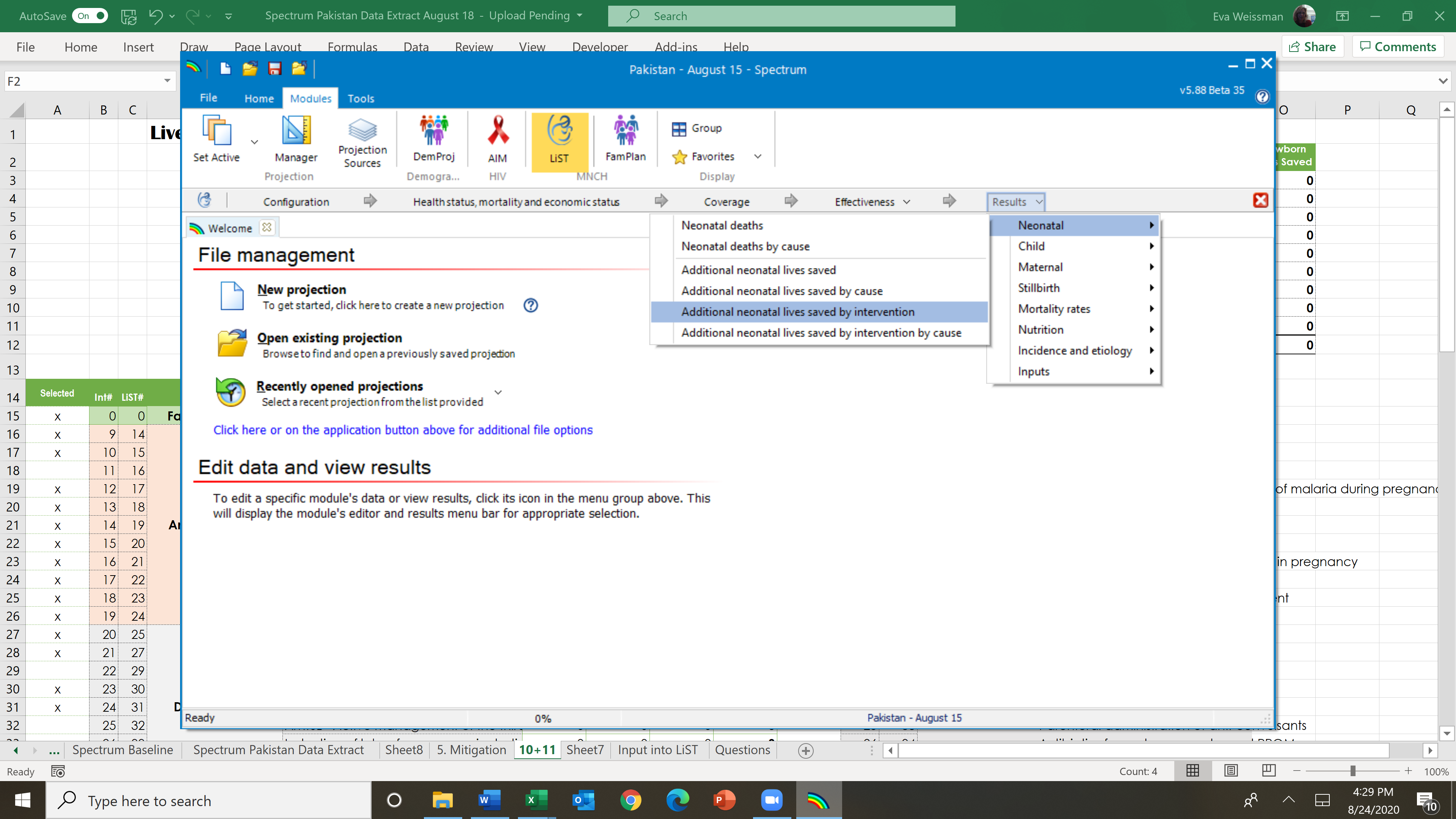


Select Table, single year, and set Final Year to 2021. Click “Show all interventions.” Click OK. The tool will calculate the data.

Right-click somewhere in the table, select “Copy All” and paste data into cell A5 on sheet **LiST Outputs WITHOUT Mitigation**. Do the same for “Additional child lives saved by intervention” (paste into cell E5) and “Additional maternal lives saved by intervention” (paste into cell I5).

Additionally needed: **Updated number of pregnancies and births**

The change in family planning use in the 2021 coverage scenario will lead to a change in the projected number of pregnancies and births, and with that, the number of women requiring the different antenatal and delivery interventions. Go to the FamPlan module, Results tab/Vital Events/Pregnancies, and in the dialog box select the options shown below (make sure to deselect disaggregation, which is not needed)


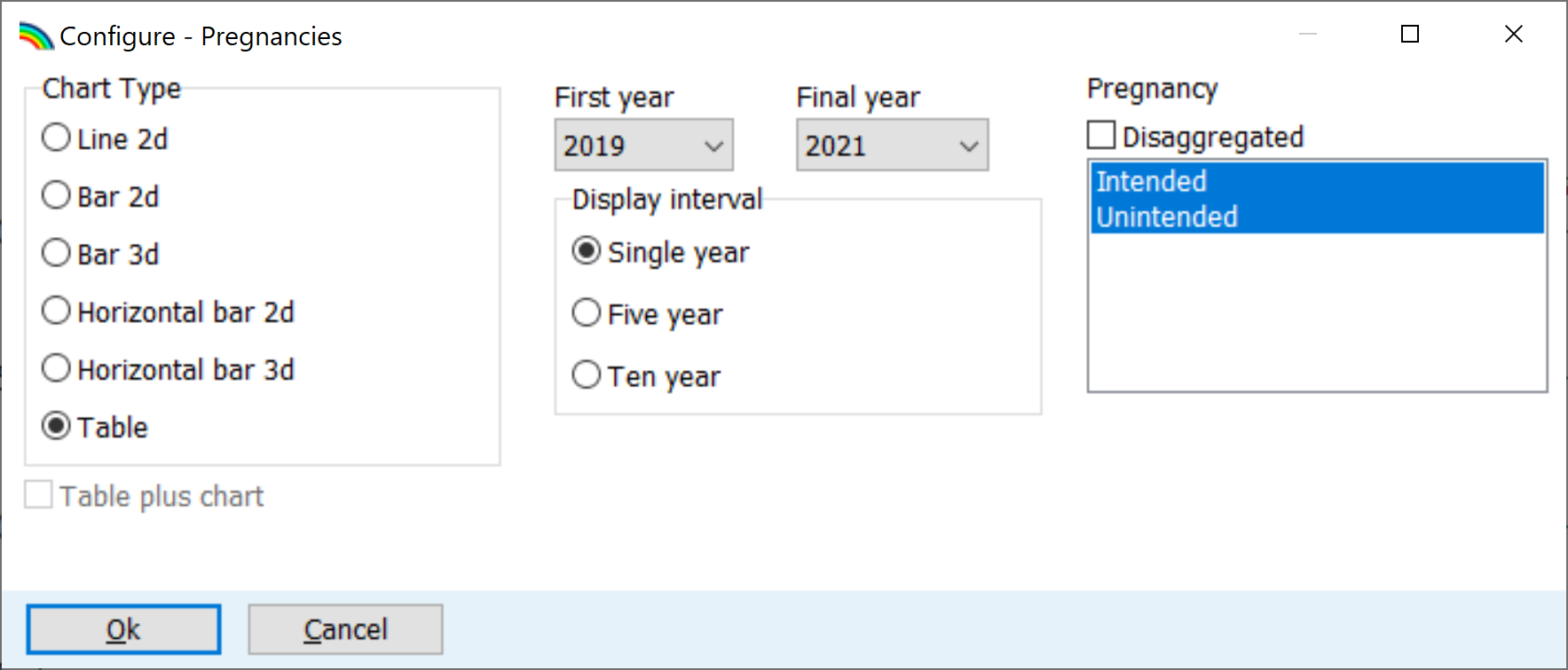


When the data table appears, go into the table, select “Copy All” and paste into cell Q26 (right under the green bar).


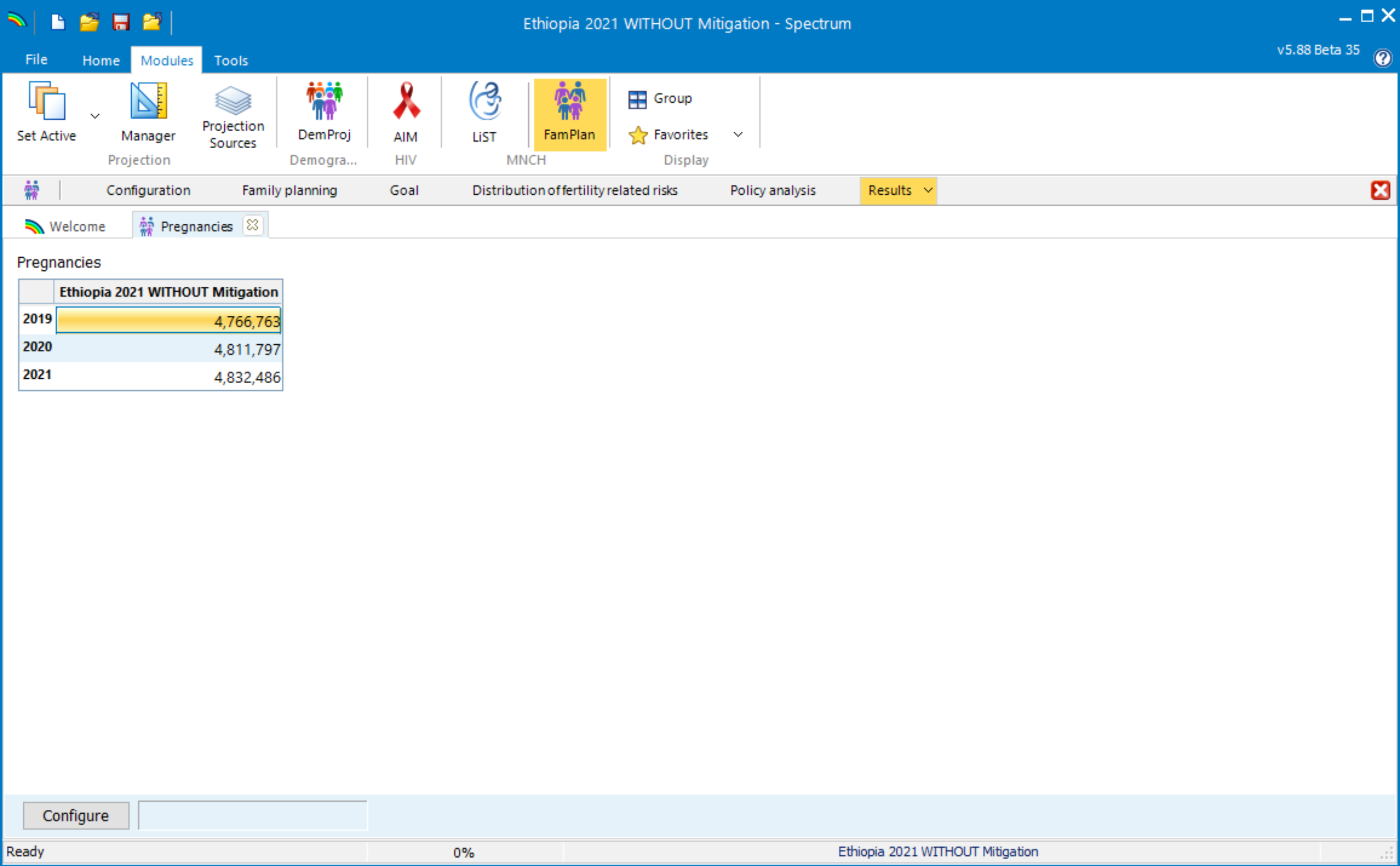


**Then do the same for births (Famplan module, Results tab/Vital Events/Births), and paste into cell Q33**

**CARRY OUT THE EXACT SAME STEPS FOR THE DATA WITH MITIGATION** (Create a LiST model version called Country Model 2019-2021 WITH mitigation)

Then move to sheet **6. Lives Lost Due to COVID, and 7. Lives Saved WITH Mitigation**, which will show the results. Sheet 7 automatically deducts the lives saved in the “WITHOUT Mitigation” scenario so the lives saved are due exclusively to the mitigation measures analyzed in the model, they don’t reflect any other factors.

1. **Number of Visits and Health Worker Contacts per Intervention**

This input is necessary to calculate the number of contacts with the health facility and health care providers (for the risk of COVID infection calculations).

The user should review the estimate of number of visits/hospital days required per intervention in the course of a year or a pregnancy, assumed the interventions are provided independently of each other (the model later makes an adjustment for interventions that are usually provided together, such as tetanus toxoid which is usually administered during one of the 4-8 antenatal care visits). Certain interventions that cannot really be provided independently are not allocated a visit number (e.g. drying of the newborn and clean cord care, which are integral part of routine delivery/newborn care)

1. **Estimation of COVID Infections and Lives Lost due to Maintaining Coverage of Essential Interventions**

The estimation of additional COVID infections that are the result of additional contacts with the health system due to the increase in coverage is done in the Excel model and does not involve LiST. Calculations are an adaptation and expansion of an approach used in a risk-benefit analysis of childhood vaccinations carried out by researchers at the London School of Hygiene and Tropical Medicine and published in June 2020^[[1]](#footnote-2)^. All the required variables and the calculations are shown in the sheet **Detailed COVID Calculations – 1**. For most analyses, only some variables need to be changed. Sheet **9. COVID Transmission Risk** shows the most important variables that should be adapted to make the projections country-specific. They include current percentage of population that has or has had COVI, expected level of COVID prevalence at which herd immunity kicks in, number of months until that herd immunity level is reached. Also required is the estimated R-naught or reproduction number for the virus, i.e. how many cases are directly generated by one infected individual.

Also required is an estimate of how much riskier a day’s stay at the hospital is than an ambulatory visit. Also, an estimate of how likely it is that an infected household member will transmit the disease to all other household members (the assumption in the original study was 100%, but newer research might show a lower level of transmission).

This information is used to calculate the excess number of infections. To calculate deaths associated with COVID, it is necessary to know average household size and composition (in the country as well the infection fatality rate by age group (children, adults and older adults – age 60+)

1. **Comparison of Lives Saved and Lost – Risk Benefit Ratios**

[…]


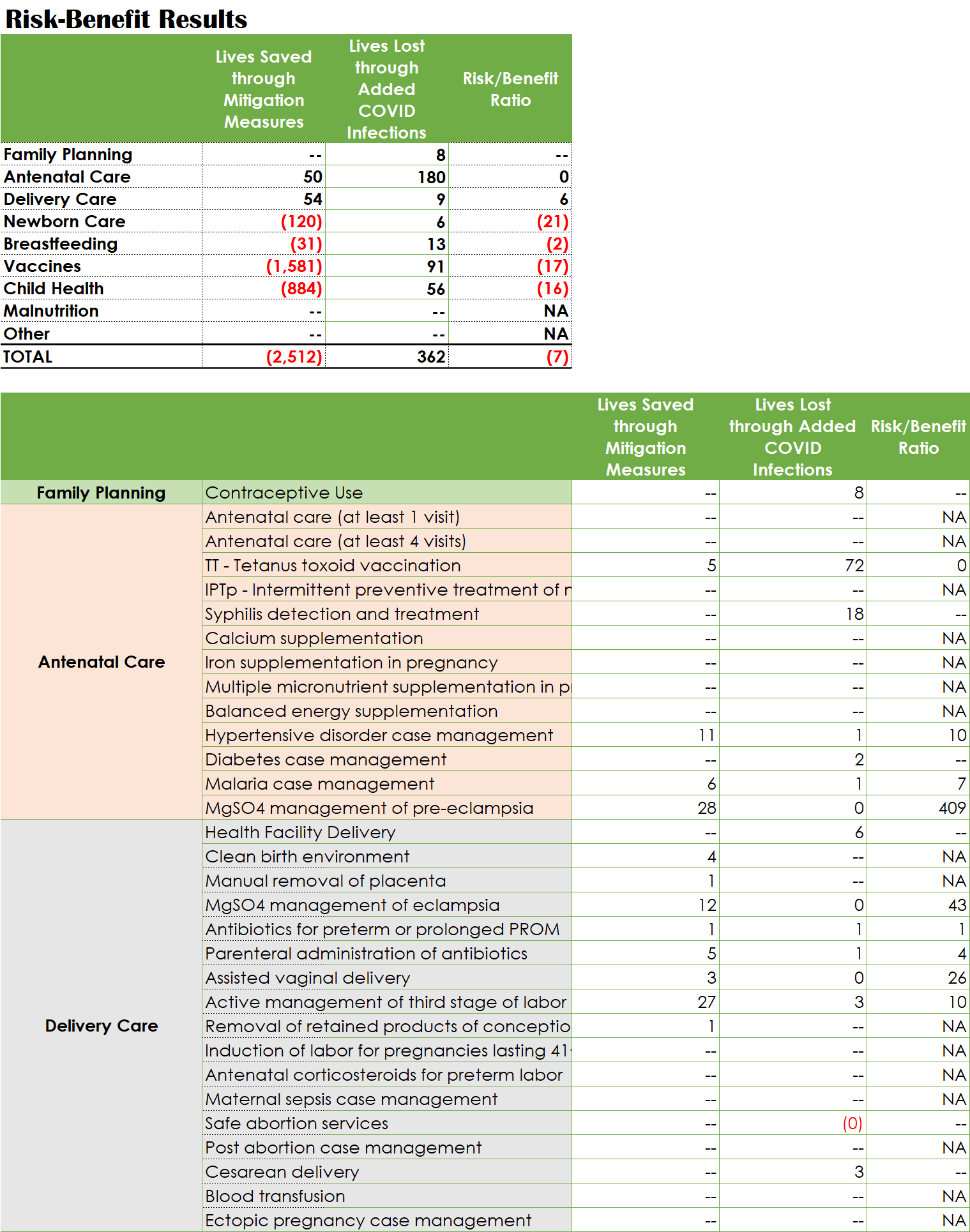
 […]

**Annex1: LiST Interventions**

*Source: https://www.jhsph.edu/research/centers-and-institutes/institute-for-international-programs/_documents/manuals/list_manual.pdf*

| Interventions | | Coverage | | Baseline Data Source | | Notes-Description | |
| --- | --- | --- | --- | --- | --- | --- | --- |
| **Periconceptual period** | | | | | | | |
| Contraception* | | see FamPlan | | FamPlan Module in Spectrum | |  | |
| Folic acid supplementation or fortification | | % of married women receiving folic acid supplementation tablet or fortification at conception | | Assumed to currently be 0 in all countries | | 5.0 mg folic acid per day for three months for women attempting to become pregnant | |
| Termination of pregnancy – D&C, anaesthesia | | % of terminations that are performed with D&C and anesthesia | | Set at 0 for baseline | |  | |
| Termination of pregnancy – vacuum aspiration | | % of terminations that are performed with vacuum aspiration | | Set at 0 for baseline | |  | |
| Termination of pregnancy - medical | | % of terminations that are performed medically | | Set at 0 for baseline | |  | |
| **Antenatal period** | | | | | | | |
| Antenatal care | | % of pregnant women with at least 4 antenatal care visits | | DHS or MPS WHR-05 if not available | | This intervention has no impact itself. The components below are the interventions which impact mortality. | |
| Case management during pregnancy | | % of pregnant women who get at least 4 ANC visits. | |  | | These visits should include the following basic activities: screening and management of anemia, screening and management of hypertension; screening and management of pre-partum hemorrhage; screening and management of abnormal lie or twins. | |
| Syphilis detection and treatment | | % of pregnant women screened for syphilis with the rapid plasma reagent test and treated with 2.4 miu benzathine penicillin, if needed. | |  | |  | |
| Calcium Supplementation | | % of pregnant women taking 1g of calcium per day | | No data currently available | |  | |
| IPT malaria | | % of pregnant women living in malaria endemic areas and receiving intermittent preventive treatment for malaria (2 doses of sulfadoxine- pryemthamine) | | MICS/DHS via Malaria and Children Report [www.unicef.org/health/file](http://www.unicef.org/health/file) s/Malaria0831.pdf | | Only applies to countries with a program recommending IPTp and 1st or 2nd child | |
| Tetanus toxoid | | % of children protected at birth from tetanus (PAB) | | WHO/UNICEF | | % of women who received 2 doses of tetanus toxoid during this pregnancy or ever: Received at least 2 doses, the last within 3 years; Received at least 3 doses, the last within 5 years; Received at least 4 doses, the last within 10 years; Received at least 5 doses during lifetime. Also known as TT2+. | |
| Balanced energy supplementation | | % of undernourished pregnant women receiving high protein and calorie dietary supplements | | set at 0 for baseline | | The proxy chosen for undernourished pregnant women is the percent of the population living on less than a dollar a day. | |
| Multiple micronutrient supplementation | | % of pregnant women receiving micronutrient supplementation | | set at 0 for baseline | | The population at risk is all pregnant women. Multiple micronutrient supplementation is defined as receiving at least three micronutrients, typically including iron, folic acid, and another nutrient, often Vitamin A. Adequate receipt is for the duration of the pregnancy. | |
| Case management of malaria | | % of pregnant women with malaria who are treated for malaria at a health center | | Set at 0 for baseline | | This is at a health center | |
| Case management of malaria | | % of pregnant women with malaria who are treated for malaria at a hospital | | Set at 0 for baseline | | This is in a hospital. | |
| HIV testing and treatment (maternal)** | | see AIM | | AIM Module in Spectrum | |  | |
| **Child Birth** | |  | |  | |  | |
| Facility based birth (InstDel) | | % of infants delivered in a facility | | DHS/MICS and MPS WHR-05, if otherwise unavailable | | This intervention has no impact itself. The components below are the interventions which impact mortality. This is used to estimate coverage below. | |
| Skilled birth attendance | | % of infants delivered by a skilled birth attendant | | DHS/MICS and MPS WHR-05, if otherwise unavailable | | This intervention has no impact itself. The components below are the interventions which impact mortality. This is used to estimate coverage below. | |
| **At onset of labor or risk of onset** | | | | | | | |
| Antenatal corticosteroids | |  | |  | | Intramuscular injection of betamethasone sodium phosphate to women with suspected premature labor (6 mg, every 12 hours for 2 days) – target 2+ doses 12 hours before birth | |
| Antibiotics for pPRoM | |  | |  | | Administration of oral erythromycin to women with premature rupture of membranes (PRoM) (250mg, 4 times daily for 7 days) who are not in labor to prevent PRoM | |
| **Labor, birth, and immediate postnatal period** | | | | | | | |
| Essential care for all women and immediate essential newborn care | | % of women with essential care during delivery and immediate newborn care | |  | | This includes: monitoring labor progress with a partograph, detection of complications and infection control via a clean delivery. Episiotomy is available, if needed. For the neonate, this includes: immediate drying and wrapping, skin-to-skin contact and thermal care as well as immediate breastfeeding initiation | |
| Basic Emergency Obstetric Care | | % of women with access to basic emergency obstetric care, if needed | |  | | This refers to management of delivery at a health center and covers case management of direct obstetric complications. The intervention includes: Case management of abortion, ectopic pregnancy, hypertensive diseases of pregnancy, ante-partum hemorrhage, prolonged/obstructed labor, post-partum hemorrhage and severe infection. Methods include: shock management, MgSO4, pain relief, ABC, parenteral antibiotics, parenteral oxytocics, IV fluids, instrumental delivery and manual removal of the placenta and retained products,. | |
| Comprehensive Emergency Obstetric Care | | % of women with access to comprehensive emergency obstetric care, if needed | |  | | This refers to management of delivery at a hospital and covers case management of direct obstetric complications. This is in addition to all interventions included in Basic Emergency Obstetric Care. This intervention includes: Case management of abortion, ectopic pregnancy, hypertensive diseases of pregnancy, ante-partum hemorrhage, prolonged/obstructed labor, post-partum hemorrhage and severe infection Additional methods include: ultrasound, culdocentesis, induction, laparotomy, salpingectomy, blood transfusion, caesarian section, hysterectomy, symphisiotomy, balloon tamponade, uterine ligature, MRVOP, surgical infection control and episiotomy. | |
| Active management of third stage of labor | | % of women with access to active management of the third stage of labor | | Set at 0 for baseline | | This includes controlled cord traction, oxytocics as well as massage. | |
| Neonatal resuscitation (facility) | |  | |  | | % of newborns with access to detection of breathing problems and resuscitation (with a bag and mask), if needed | |
| Home Delivery | | 1-% of infants born in any facility | | fixed by % born in facility | | This intervention has no impact itself. The components below are the interventions which impact mortality. | |
| Clean practices in non-facility birth and immediate, essential newborn care | | % of women delivering at home with a clean delivery kit, including promotion of clean cord practices | |  | | Need a definition. | |
| Newborn resuscitation | | % of women delivering at home with access to newborn resuscitation | | Set at 0 for baseline | | % of newborns with access to detection of breathing problems and resuscitation (with a mucus extractor), if needed | |
| **Immediate Postnatal Care** | | | | | | | |
| Preventive postnatal care (healthy practices & illness detection) | | % of infants delivering at home with a postnatal health contact/visit within 2 days of birth | | set at 0 for baseline | | This intervention includes being counseled on breast feeding, clean cord care, skin hygiene and temperature control, as well as detection of illnesses and extra care for low birth weight infants. | |
| Kangaroo mother care | | % of low birth weight infants with access to kangaroo mother care | | set at 0 for baseline | | Kangaroo mother care is defined as: skin-to-skin contact between a mother and her newborn, frequent and exclusive breast feeding, and early discharge from the hospital. Note that this intervention only impacts deaths attributable to prematurity and must be given in a facility. | |
| Active early detection of maternal and neonatal complications | | % of mothers with a post-natal visit within 2 days | | set at 0 for baseline | | This intervention includes detection of post-partum hemorrhage, hypertensive disease of pregnancy and sepsis. This also includes detection of neonatal sepsis. | |
| Postpartum hemorrhage reduction | |  | | set at 0 for baseline | | This intervention includes nipple stimulation, rubbing of the uterus and discussion of breastfeeding. | |
| PMTCT** | | see AIM | | AIM Module in Spectrum | |  | |
| **Breastfeeding** | |  | |  | |  | |
| Breastfeeding behavior | | See breast feeding under national level data inputs | | DHS or MICS | | Note that this refers to the actual breastfeeding behavior, which can change based upon the age of the child as well as the observed/desired behavior. | |
| Breastfeeding promotion | | % of mothers of children 0-11 months of age exposed to a breastfeeding promotion message | | Baseline is percent of 1-5 month old children exclusively breastfed | | Breastfeeding promotion can be either one-on-one or group meetings. It is assumed that children 1-5 months of age who are exclusively breast fed do not need breastfeeding promotion. | |
| **Preventive** | |  | |  | |  | |
| Complementary feeding--education only | | % of mothers intensively counseled on the importance of continued breast feeding after 6 months and appropriate complementary feeding practices | | Set at 6-9 month old children receiving breastmilk and complementary feeding as baseline; See FAQ for notes on indicator selection. | | This intervention only benefits children 6-24 months of age who are living on more than a dollar a day; This can be delivered in the home, community or clinic, by health professionals or health volunteers. It includes the assumption that breast feeding should be continued for children 6-24 months of age,(but does not affect breast feeding rates). The intervention includes education on the proper foods to prepare as well as appropriate hygiene for food preparation. | |
| Complementary feeding-- supplementation and education | | % of mothers of malnourished infants who are intensively counseled on the importance of continued breast feeding after 6 months and appropriate complementary feeding practices as well as given appropriate supplements | | set at 0 for baseline. | | This intervention only benefits children 6-24 months of age who are living on less than a dollar a day; This can be delivered in the home, community or clinic, by health professionals or health volunteers. It includes the assumption that breast feeding should be continued for children 6-24 months of age,(but does not affect breast feeding rates). The intervention includes supplementation of child, ranging from 100-1500 kcal per day, typically including micronutrients. As well as education on the proper foods to prepare and appropriate hygiene for food preparation. | |
| Use of water connection in the home | | % of households with water piped into the home or yard | | Childinfo.org (2006 estimate) or DHS/MICS | | The baseline data was from ChildInfo.org. When a DHS/MICS was available closer to 2003, this was used instead | |
| Use of improved water source within 30 minutes | | % of homes with improved water | | Childinfo.org (2006 estimate) or DHS/MICS | | The baseline data was from ChildInfo.org. When a DHS/MICS was available closer to 2003, this was used instead. See [www.unicef.org/wes/mdgreport/definition.php](http://www.unicef.org/wes/mdgreport/definition.php) for indicator definitions. | |
| Improved excreta disposal (latrine/toilet) | | % of homes with access to an improved latrine or flush toilet | | Childinfo.org (2006 estimate) or DHS/MICS | | The baseline data was from ChildInfo.org. When a DHS/MICS was available closer to 2003, this was used instead. See [www.unicef.org/wes/mdgreport/definition.php](http://www.unicef.org/wes/mdgreport/definition.php) for indicator definitions. | |
| Hand washing with soap | | % of mothers washing their hands with soap appropriately | | Curtis VA, Health Education Research, March 2009; all others set to 0 | | Appropriate hand washing is defined as washing hands with soap, ash or other materials and using adequate water, after handling feces and before preparing food. Reported hand washing is not an adequate indicator. Neither is availability of hand washing materials. Observational data is required. | |
| Hygienic disposal of children's stools | | % of children whose fecal matter is adequately contained | | DHS survey | | 1. Children’s tools are considered to be contained if the child always uses a toilet/latrine, the feces are thrown in a toilet/latrine, the feces are buried in the yard | |
| Insecticide treated materials or indoor residual spraying | | % of households with at least 1 insecticide treated net or covered by indoor residual spraying | | MICS/DHS via Malaria and Children Report [www.unicef.org/health/file](http://www.unicef.org/health/file) s/Malaria0831.pdf | |  | |
| Vitamin A supplementation | | % of children 6-59 months receiving full coverage with Vitamin A | | Childinfo.org | | Full coverage of Vitamin A supplementation is considered to be 2 doses of Vitamin A in the past year. See above definition of national Vitamin A deficiency. It is assumed that all children in a country with Vitamin A deficiency are in need of Vitamin A for prevention. | |
| Zinc for prevention | | % of children 6-59 months supplemented daily with zinc | | set at 0 for baseline | | Daily supplementation with 10mg zinc. It is assumed that all children in a country with zinc deficiency are in need of zinc for prevention. See above definition of national zinc deficiency. | |
| **Vaccinations** | |  | |  | |  | |
| Rotavirus vaccine | | Proportion of infants having received 3 doses of rotavirus vaccine prior to the survey | | set at 0 for baseline | | Not yet implemented in countries routinely | |
| Measles vaccine | | Proportion of infants having received 2 dose of measles containing vaccine (MCV) prior to the survey | | UNICEF | |  | |
| Hib vaccine | | Proportion of infants having received 3 doses of Haemophilis influenza type B vaccine prior to the survey | | UNICEF | |  | |
| Pneumococcal vaccine | | Proportion of infants having received 3 doses of pneumococcal vaccine prior to the survey | | set at 0 for baseline | | Will be implemented in some countries routinely in 2009. | |
| DPT3 vaccination | | Proportion of infants having received 3 doses of diphtheria, tetanus and pertussis vaccine prior to the survey | | UNICEF | |  | |
| Polio vaccine | | Proportion of infants having received 3 doses of polio vaccine prior to the survey | | UNICEF | | Polio vaccine has no impact on mortality of children less than 5 years of age. | |
| BCG vaccine | | Proportion of infants having received 1 dose of BCG vaccine prior to the survey | | UNICEF | | BCG vaccine has no impact on cause specific mortality of children less than 5 years of age. | |
| **Curative** | |  | |  | |  | |
| Oral antibiotic case management of severe infection | | Proportion of neonates with suspected | | Set at 0 or ½ of case management of | |  | |
| in neonates | | pneumonia, sepsis or ARI in the 2 weeks preceding the survey treated with antibiotics | | pneumonia, for baseline | |  | |
| Injectable antibiotic case management of severe infection in neonates | | Proportion of neonates | | Set at 0 for baseline | |  | |
| Case management of severe infection in neonates with full supportive care | | Proportion of neonates with serious infections with oxygen, IV antibiotics, IV fluids, blood transfusion, phototherapy, etc. available | | set as a function of facility births | | Facility based care only | |
| Case management of pneumonia (oral antibiotics) | | Proportion of children 1-59 months with suspected pneumonia or ARI treated with antibiotics | | Childinfo.org; DHS | | This is not available for many recent DHS surveys. | |
| ORS | | % of children with diarrhea given ORS from sachets | | DHS | | This includes sachets or pre-mixed solutions of ORS. | |
| Antibiotics for dysentery | | % of children with dysentery treated with antibiotics | | set at case management for pneumonia, if available, otherwise 50% of ORS | | Typical treatment is 3 days of 250mg of ciprofloxacin. This data is typically not available. Use DHS data if available. | |
| Zinc for treatment | | % of children 0-59 months with diarrhea receiving zinc supplementation | | set at 0 for baseline | | 20mg of zinc supplementation daily for 14 days | |
| Therapeutic feeding | | % of wasted children receiving therapeutic feeding | | Set at 0 for baseline | | Therapeutic feeding is inpatient treatment including antibiotics, supplementation with food and maternal education. | |
| Vitamin A for measles treatment | | % of measles cases treated with Vitamin A | | Set at the percent of children receiving 2 doses of Vitamin A, UNICEF, as baseline | | Typical treatment is 2 days of Vitamin A supplementation, ranging from 50,000 IU to 200,000 IU, based upon the age of the child. This information is not typically available. | |
| Antimalarials | | Proportion of children 0-59 months with a fever receiving any appropriate anti-malarial | | Childinfo.org | | This data is from the DHS surveys. | |
| Cotrimoxazole** | | see AIM | | AIM Module in Spectrum | |  | |
| Child ART** | | see AIM | | AIM Module in Spectrum | |  | |

1. *Abbas K. et al. 2020. Benefit-risk analysis of health benefits of routine childhood immunisation against the excess risk of SARS-CoV-2 infections during the COVID-19 pandemic in Africa. The Lancet Global Health doi: 10.1016/S2214-109X(20)30308-9* [↑](#footnote-ref-2)
