# Supplementary material for: Benefit-risk analysis of maintaining essential Reproductive, Maternal, Newborn, and Child Health (RMNCH) services against risk of COVID-19 infection
Source: PLOS Glob Public Health. 2022 Feb 9;2(2):e0000176. doi: 10.1371/journal.pgph.0000176 (PMC10021309; doi:10.1371/journal.pgph.0000176)
Supplement: S2 Text — (DOCX) [file pgph.0000176.s004.docx]

Key Formulas of Risk-Benefit Model

**Sheet Intro**

Cell B3: Dropdown-List.
Underlying country list is stored on sheet HH Database! Cells $S$2:$S$80. To make any changes to the countries on that list, change or add names there.

Cell B9: Dropdown List defined in dropdown list dialog box itself

Setup Model button runs macro to set the model up for the chosen scenario.

**Sheet 1. Select Services**

This sheet contains no formulas, but there are a few macro buttons.


 Expands table to show the interventions included in the different packages

 Runs macro that will hide all non-selected interventions throughout the rest of the model

 Resets this sheet to just show packages again, and resets model (previously hidden interventions are shown again)

**Sheet Spectrum Baseline Data**

Columns A:R data copied-in from data dump/extraction from Spectrum country model.

Columns AA:AG pull out relevant information from the data dump and arrange it for the Excel model.

Some of the names used in the Spectrum output are different from the ones used in the Excel model so the model shows the name used in Spectrum in Column AF next to the name used in the Excel model (Column AE).

Column AG contains the VLOOKUP formula that pulls in the needed information from Columns A:R

Cell AG16 down: =VLOOKUP($AF16,$A$1:$B$20000,**2,**FALSE)

Looks up LiST intervention name in Column A, then picks the value in the cell next to it, which presents 2019 coverage.

For vaccinations, the formula is slightly different, because coverage for vaccinations in the Spectrum data dump starts in the year 2015, so the year needed for the Excel model is in the 6^th^ column, not the second as for all the other interventions.

Cells AG61 through AG71; Lookup table is expanded (from A:B to A:F), and lookup row changed, from 2 to 6

= VLOOKUP($AF61,$A$1:$**F**$20000,**6**,FALSE)

Changes made from Version 5.89 to 6.02:

**Intervention Name Changes**

| ***Version 5.89*** | ***Version 6.02*** |
| --- | --- |
| Folic acid supplementation/fortification | Folic acid fortification |
| Blanket iron supplementation/fortification | Iron fortification |
| IPTp - Intermittent preventive treatment of malaria during pregnancy | Prevention of malaria in pregnancy |
| Parenteral administration of anti-convulsants | MgSO4 for eclampsia |
| Measles – One dose | *Measles – One dose |

**Sheet Demographic and Epi Data**

Cells H87, H89, H90: Conversion of 2-weekly prevalence into annual number of cases of diarrhea and ARI:

This is done by first converting the 2-week prevalence data to 2-week incidence data, which is then multiplied by 26 to get to annual incidence data and the multiplied by the number of children under 5.

=$H$10*14/19*26*$F87


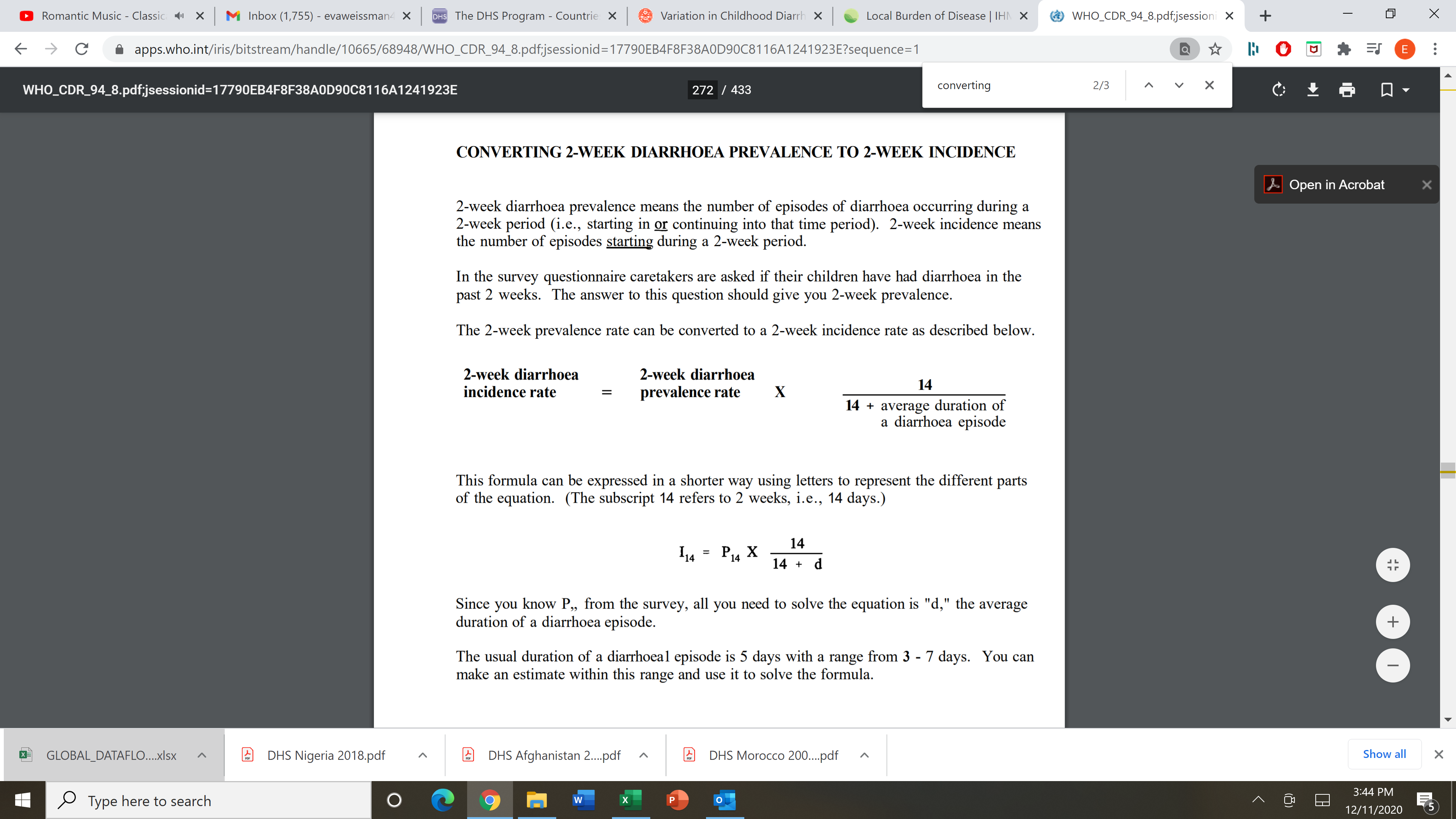


Source: WHO. 1994. Household Survey Manual: Diarrhea and Acute Respiratory Infections. https://apps.who.int/iris/bitstream/handle/10665/68948/WHO_CDR_94_8.pdf;jsessionid=17790EB4F8F38A0D90C8116A1241923E?sequence=1

Other possible ways data might be available: incidence rate, e.g. 7,300 episodes per 1,000 population per year (=0.73 episode per person per year). 2-week incidence would be calculated by dividing number of episodes by 26 (ignores seasonal variations)

Cells H91: Conversion of 2-weekly prevalence into annual number of potential malaria cases:

=$H$10*$F$97*14/19*26*$F$98

Requires two inputs: Number of episodes of fever and % of kids with fever that require malaria medication

**Sheet 2. COVID Impact on Coverage**

This sheet mainly contains user input cells. The user can choose between entering reduction by package (Family planning, ANC, delivery care) or by individual health intervention (FP – pill, FP – condoms, etc.). The choice is made by selecting either “By Package” or “For each intervention separately” from the drop-down box and then pressing the blue “Show Table” button. This will show the applicable table.

**Disruption rates by Intervention**

If the user chooses to enter disruption rates by intervention, not package, the disruption rates in the bottom table on this sheet are originally linked to the package-specific disruptions entered in the table at the top. For example, all eight family planning interventions are originally linked to the disruption percentage entered for the FP package in cell E7, all pregnancy-related interventions are linked to the ANC coverage disruption in cell E8, etc.

The one exception is FP -Traditional methods in cell I26. The assumption is that service disruption does not affect traditional method use, it might actual increase it. The current default setting is a 0% impact.

If one assumes that traditional FP method use actually increase during the pandemic, the formula can be changed to

=E$7 *(-50%)

In the given example, traditional method use would increase by 50% (the % chosen is up to the user)

**HIdden columns A:C:**

These columns appear in several of the following sheets. They are used by the macro to define what rows/intervention to show based on what the user selected on Sheet 1. Select Services.

**Column A: Selected interventions**

Cell A19=IF(VLOOKUP(B19,'1. Select Services'!$A$6:$E$77,5,FALSE)="x","x","")

Checks whether user selected the intervention (referenced by intervention number in column B) on the 1. Select Services sheet to be included in the analysis. If yes, it shows an x in that cell, if not, it leaves the cell blank).

The cells are referenced by the Setup macro (on sheet 1. Select Services) that runs through all the tables in the model and checks for x’s in Column A. If there is none, that row is hidden.

**Column B: Intervention Number in this Model**

**Column C: Intervention Number in LiST (different order than in this model)**

**Sheet 3. Mitigation Impact on Coverage**

**Columns A-BW contain user input.**

Column A contains the different mitigation strategies and Columns B-D contain estimates of the impact of these mitigation strategies on
a) the risk of COVID transmission (Columns B+C) and

b) health service coverage (Column D).

In Columns E-BW an “x” marks which health interventions are affected by the different mitigation strategies.

This first section is followed by three sections that add up the impacts across the mitigation strategies (no user input).

**Cumulative impact on number of visits (Columns BY:EQ)**

Cell BY8: =IF(E8="x",$B8,"")

If mitigation strategy applies to this intervention, puts % from column B as entered by user

Cell BY7: =SUM(BY8:BY34)

Adds up all % reductions in number of visits per intervention

**Impact on Infection Risk (Columns ES:HK)**

Cell ES8: =IF(E8="x",$C8,"")

If mitigation strategy applies to this intervention, puts % from column C as entered by user

Cell ES7: =SUM(ES8:ES34)

Adds up all % reductions in infection risk per intervention

**Cumulative impact on coverage (Columns HM:KE)**

Cell HM8: =IF(E8="x",$D8,"")

If mitigation strategy applies to this intervention, puts % from column D as entered by user

Cell HM7: =SUM(HM8:EM34)

Add up all % improvements in coverage

**% Improvement in Coverage Attributable to Individual Interventions (Columns KG:MY)**

Cell KG8: =IFERROR(HM8/HM$7,"")

Calculates what percent of total coverage increase in intervention is due to specific mitigation measures

**Sheet 4. Coverage Review**

Like many of the following sheets, this sheet has 3 hidden columns (A-C), which are used to tie all the calculations together. None of the VLOOOKUP formulas used throughout the model use the actual health interventions names to look up values, but use the intervention numbers assigned to those interventions. (For a complete list of what numbers represent what intervention, see the “Interventions” sheet)


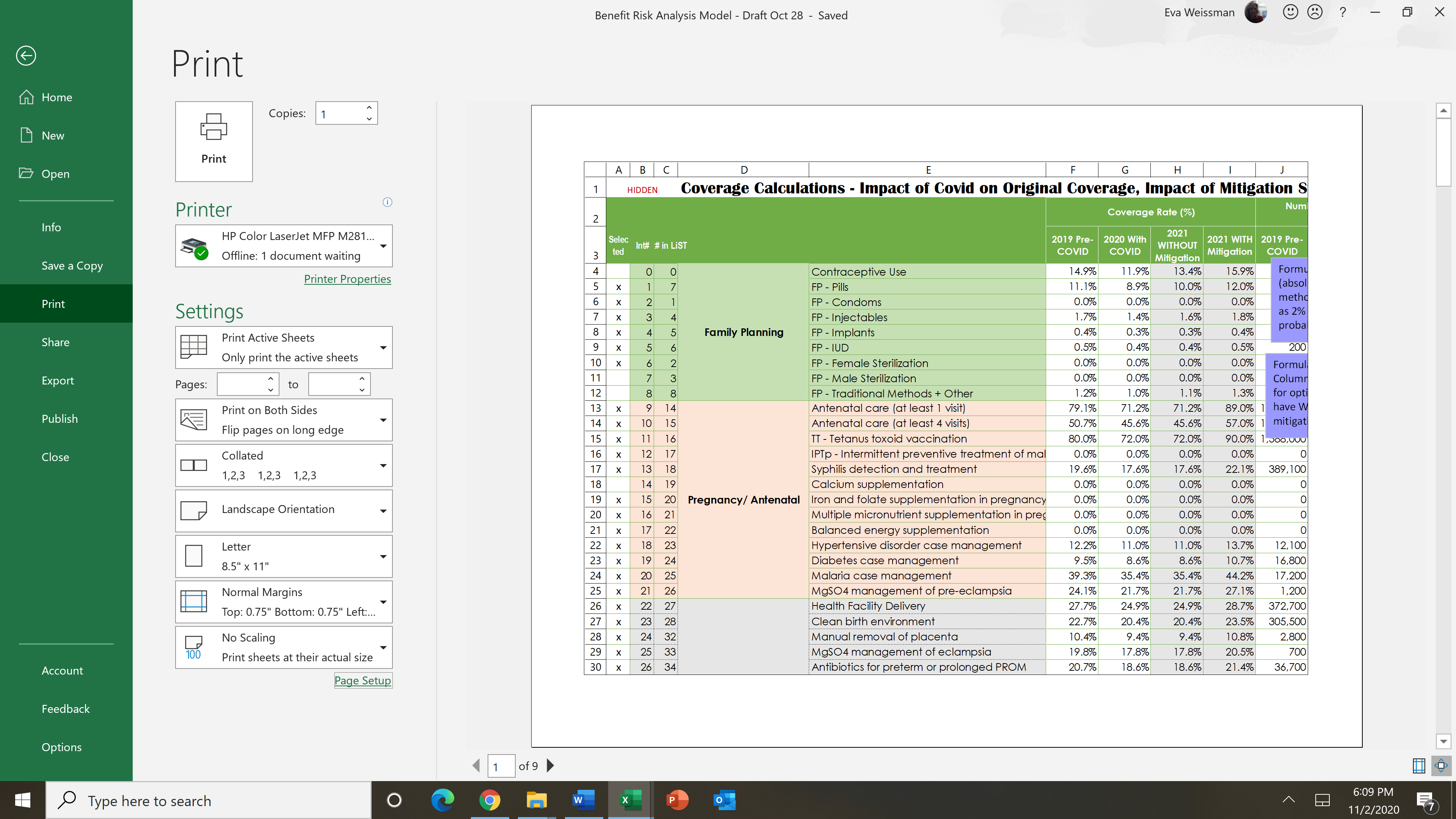


**Column A: Selected interventions**

Cell A5: =IF(VLOOKUP(B5,'1. Select Services'!$A$6:$E$77,5,FALSE)="x","x","")

Checks whether intervention was selected on Sheet 1 (with an “x” in the 5^th^ column) and if yes, puts an “x” in this cell.

This is used by the macro connected to the orange button on Sheet 1, which hides all non-selected interventions throughout the model (i.e. all rows that with interventions that don’t show an “x” in Column A.

**Column B: Intervention Number in this Model**

**Column C: Intervention Number in LiST (different order than in this model)**

**Column F: Coverage Pre-COVID 2019**

Cell F5: =VLOOKUP(B5,'Review Dem +Epi Data'!$B$14:$F$21,5,FALSE)

Looks up baseline coverage for intervention (via intervention number specified in Column B) in table on Sheet 'Review Dem +Epi Data'

**Column G: Coverage Disrupted in 2020**

Cell G5: =IF(Package=1,$F5*(1-'2. COVID Impact on Coverage'!$E$7),$F5*(1-VLOOKUP(B5,'2. COVID Impact on Coverage'!$B$19:$I$92,8,FALSE)))

If user selected to enter coverage disruption “by package” on Sheet 2 in cell F3 (if Package = 1), multiplies pre-COVID coverage with coverage reduction specified for respective package (in the table in rows 6:15), if not (if user selected “by intervention” on that sheet), multiplies pre-COVID coverage with coverage reduction specified by intervention (in table in rows 17-92).

Exception: Traditional FP will always refer to the individual intervention disruption on the interruption sheet regardless of whether the user selected disruption by package or individual intervention

Cells G12: =$F12*(1-VLOOKUP(B12,'2. COVID Impact on Coverage'!$B$19:$I$92,8,FALSE))

**Column H: Coverage 2021 WITHOUT Mitigation**

Cell H5: = G5

Same as Coverage in 2020 (with pandemic disruption)

**Column H: Coverage 2021 WITH Mitigation**

I5=MIN($H5*(1+HLOOKUP($B5,'3. Mitigation Impact on Coverag'!$HM$5:$KE$7,3,FALSE)),IF(F5*102%>100%,100%,F5*102%))

This formula does two things: 1. It increases 2020 pandemic-affected coverage by the positive impact of applicable mitigation measures and 2. It ensures that the resulting coverage rate does not exceed 100% or 102% of the initial coverage pre-COVID.

HLOOKUP formula: Finds intervention number (from Column B) in row 5 on the Mitigation Impact on Coverage sheet, then goes down three cells to row 7 to retrieve the aggregated coverage increase.

**Sheet LiST Outputs WITHOUT M.**

**Columns A:N**

Data drop from LiST model

**Table Column AA:AM**

LiST data dump arranged for Excel model (data subsequently used for Sheets 5. Lives Lost Due to Disruption and 6. Lives Saved Due to Mitigation)

**Lives lost in 2020 due to Disruption (Columns AE:AG):**

Ex. Folic acid supplementation

Newborn Lives Lost (AE7): =VLOOKUP($AD7,$A$7:$D$82,3,FALSE)

Child Lives Lost (AF7): =VLOOKUP($AD7,$F$7:$I$82,3,FALSE)

Maternal Lives Lost (AG7): =VLOOKUP($AD7,$K$7:$N$82,3,FALSE)

Formulas look up value in third column of LiST data dump (lives lost in year 2020)

**Lives lost in 2021 WITHOUT Mitigation (Columns AH:AJ)**

Ex. Folic acid supplementation

Newborn Lives Saved (AE7): =VLOOKUP($AD7,$A$7:$D$82,4,FALSE)

Child Lives Saved (AF7):=VLOOKUP($AD7,$F$7:$I$82,4,FALSE)

Maternal Lives Saved(AG7): =VLOOKUP($AD7,$K$7:$N$82,4,FALSE)

Formulas look up value in fourth column of LiST data dump (lives lost in 2021). LiST calculates lives lost/saved in relationship to the baseline year (in this case 2019, not to 2020) so these numbers will later be compared to the 2021 WITH mitigation numbers to calculate the numbers of lives saved through mitigation.

**Sheet LiST Outputs WITH M.**

**Columns A:N**

Data drop from LiST model, same as on WITHOUT Mitigation sheet.

**Table Column AA:AM**

LiST data dump arranged for Excel model (data subsequently used for Sheets 5. Lives Lost Due to Disruption and 6. Lives Saved Due to Mitigation)

**Lives lost in 2020 due to Disruption (Columns AE:AG):**

Ex. Folic acid supplementation

Newborn Lives Lost (AE7): =VLOOKUP($AD7,$A$7:$D$82,3,FALSE)

Child Lives Lost (AF7): =VLOOKUP($AD7,$F$7:$I$82,3,FALSE)

Maternal Lives Lost (AG7): =VLOOKUP($AD7,$K$7:$N$82,3,FALSE)

Formulas look up value in third column of LiST data dump (lives lost in year 2020)

**Lives saved WITH Mitigation (Columns AH:AJ)**

Ex. Folic acid supplementation

Newborn Lives Saved (AH7): = VLOOKUP($AD4,$A$7:$D$82,4,FALSE)-VLOOKUP($AD4,'LiST Outputs - WITHOUT M.'!$A$7:$D$82,4,FALSE)

Child Lives Saved (AI7): = VLOOKUP($AD4,$F$7:$I$82,4,FALSE)-VLOOKUP($AD4,'LiST Outputs - WITHOUT M.'!$F$7:$I$82,4,FALSE)

Maternal Lives Lost (AJ7): = VLOOKUP($AD4,$K$7:$N$82,4,FALSE)-VLOOKUP($AD4,'LiST Outputs - WITHOUT M.'!$K$7:$N$82,4,FALSE)

Lives saved in 2021 with mitigation measures (from columns AH:AJ) compared to lives saved/lost without mitigation in 2021

**Sheet 5. Lives Lost Due to Disruption**

Pulls together the data from the LiST Output WITH M sheet with VLOOKUP formulas using the LiST reference number of the respective interventions (hidden in Column C) as the lookup value.

**Lives lost:**

**Looks up lives lost from LiST Output sheet**

Newborn Lives: =VLOOKUP($C16,'LiST Outputs WITH M.'!$AA$4:$AJ$82,5,FALSE)

Child Lives: =VLOOKUP($C16,'LiST Outputs WITH M.'!$AA$4:$AJ$82,6,FALSE)

Maternal Lives: =VLOOKUP($C16,'LiST Outputs WITH M.'!$AA$4:$AJ$82,7,FALSE)

Several health interventions save lives through different mechanisms, e.g. family planning works through contraceptive use, maternal age and birth order. Complementary feeding combines results of appropriate complementary feeding and reduction in stunting as well as reduction in wasting.

**FAMILY PLANNING**

**Addition of lives lost through maternal age and birth order**

LiST shows lives saved through family planning in 3 ways: through increase in FP use, changes in maternal age and birth order. The 3 impacts need to be added together to give the full impact of an increase in FP coverage.

=VLOOKUP($C15,'LiST Outputs WITH M.'!$AA$4:$AJ$82,5,FALSE)+'LiST Outputs WITH M.'!AE5+'LiST Outputs WITH M.'!AE6

Complementary Feeding:

Cell F58=SUM('LiST Outputs WITH M.'!AE44:AE47)

For some interventions, LiST does not report lives saved individually, but for a combination of related interventions. Examples for this are the newborn interventions “Kangaroo Mother Care” and Full supportive Care of Prematurity”. Results are reported together for the two under “Case management of premature babies.” The same applies to the three subinterventions of “Case management of neonatal sepsis/pneumonia.”

**Sheet 6. Lives Saved WITH Mitigation**

**Lives saved by Intervention:**

**ALL INTERVENTIONS EXCEPT FAMILY PLANNING**

**Looks up lives saved from LiST Output sheet (depending on scenario selected, if regular recovery not included, in Columns 8-10, otherwise in Columns 11-13)**

Newborn Lives

=IF(Counterfactual="No mitigation",VLOOKUP($C16,'LiST Outputs WITH M.'!$AA$4:$AM$82,8,FALSE),VLOOKUP($C16,'LiST Outputs WITH M.'!$AA$4:$AM$82,11,FALSE))

Child Lives

IF(Counterfactual="No mitigation",,VLOOKUP($C16,'LiST Outputs WITH M.'!$AA$4:$AM$82,9,FALSE),VLOOKUP($C16,'LiST Outputs WITH M.'!$AA$4:$AM$82,12,FALSE))

Maternal Lives

=IF(Counterfactual="No mitigation",,VLOOKUP($C16,'LiST Outputs WITH M.'!$AA$4:$AM$82,10,FALSE),VLOOKUP($C16,'LiST Outputs WITH M.'!$AA$4:$AM$82,13,FALSE))

**FAMILY PLANNING**

**Addition of lives saved through maternal age and birth order**

Newborn Lives

=IF(Additional_Scenario="No",VLOOKUP($C15,'LiST Outputs WITH M.'!$AA$4:$AM$82,8,FALSE)+'LiST Outputs WITH M.'!AH5+'LiST Outputs WITH M.'!AH6,VLOOKUP($C15,'LiST Outputs WITH M.'!$AA$4:$AM$82,11,FALSE)+'LiST Outputs WITH M.'!AK5+'LiST Outputs WITH M.'!AK6)

Child Lives

=IF(Additional_Scenario="No",VLOOKUP($C15,'LiST Outputs WITH M.'!$AA$4:$AM$82,9,FALSE)+'LiST Outputs WITH M.'!AI5+'LiST Outputs WITH M.'!AI6,VLOOKUP($C15,'LiST Outputs WITH M.'!$AA$4:$AM$82,12,FALSE)+'LiST Outputs WITH M.'!AL5+'LiST Outputs WITH M.'!AL6)

Maternal Lives

=IF(Additional_Scenario="No",VLOOKUP($C15,'LiST Outputs WITH M.'!$AA$4:$AM$82,10,FALSE)+'LiST Outputs WITH M.'!AJ5+'LiST Outputs WITH M.'!AJ6,VLOOKUP($C15,'LiST Outputs WITH M.'!$AA$4:$AM$82,13,FALSE)+'LiST Outputs WITH M.'!AM5+'LiST Outputs WITH M.'!AM6)

**Lives saved by Package:**

Lives saved by interventions shown in table below added up into packages

**Sheet Detailed COVID Calculations - 1**

This sheet contains all the variables used in the calculations of excess COVID infections that might happen to patients traveling to, waiting and receiving services at a health facility.

The bottom tables shows how infections are estimated for 3 types of visits:

1. Child ambulatory health facility visit (child + caretaker)
2. Adult ambulatory health facility visit
3. Adult hospital stay

The methodology has been adapted from Abbas, K. et al. (See box on next two pages)

The formula on the Detailed Calculations – 1 sheet are just shown to elucidate the formulas used, the actual calculations by intervention used for the Risk-Benefit Analysis are on the following sheet – **Detailed COVID Calculations – 2 in Columns Z - AC**

**Probability of getting infected on the way to the clinic or waiting at the clinic:**

100% - (100% - risk of community members being infectious x transmission risk in community)^Number of children going to the clinic for the intervention x Number of visits per intervention x Number of contacts during trip to facility x 2 (child + caretaker)

**Probability of getting infected during contact with health care provider at the clinic:**

100% - (100% - risk of health care provider being infectious x probability of transmission)^Number of children going to the clinic for the intervention x Number of visits per intervention x 2 (child + caretaker)

**Risk Calculations Used by London School of Hygiene & Tropical Medicine**

**Abbas, K. et al. June 2020. Benefit-risk analysis of health benefits of routine childhood immunisation against the excess risk of SARS-CoV-2 infections during the Covid-19 pandemic in Africa. Xxx**

**A2. Covid-19 risk model**

The risk of infection with SARS-CoV-2 depends on the stage of the epidemic, with relatively higher risk during the early incline phase of the epidemic and larger proportion of susceptible population and relatively lower risk during the late decline phase of the epidemic and smaller proportion of susceptible population. We refer excess risk to additional infections among households that are attributable to the vaccination visits, that these additional infections among household members would not have occurred during the course of the epidemic if not for the vaccination visits.

As a base case, we assume that through contact reducing interventions, community SARS-CoV-2 transmission will be spread over a period (*T*) of 5 to 6 months and the exposure risk is constant during that time due to contact-reducing interventions successfully mitigating sharp peaks in disease (Table A1) [3]. We assume that these measures will be gradually lifted and that, in the absence of vaccination visits, between *Θ = 40%* and *Θ = 70%* of the population will have been infected with SARS-CoV-2. This corresponds for example to the herd immunity threshold for a basic reproduction number (*Ro*) of between 1.6 and 3.6 assuming that everyone who is infected develops full immunity. Partial immunity following infection combined with a reduction in effective reproduction number following physical distancing measures would also achieve a final epidemic size of around this level. It follows from above that between 30% and 60% of the population would not have become infected with SARS-CoV-2 independent of whether or not the infants in their households had attended routine childhood vaccination. Furthermore, if after 6 months 60% of the population was infected then, assuming a duration of infectiousness (*Ψ*) of one week [4] and a reasonably flat epidemic curve, then on any given day about *po ~ 2%* of the population would be infected and potentially transmitting. In comparison to community members, we assume that vaccinators are at higher risk of being infected (between 1 and 4 times, *pv* = *ι1 po*) because of their higher frequency of exposure to other people, but at lower risk of onward transmission (between 0.25 and 1 times, *tv* = *ι2 to*) because most of their contacts with vaccinees are brief, and they have enhanced risk awareness and use corresponding protective measures including basic respiratory hygiene and personal protective equipment as available. Also, we assume that an infant child and the parent or adult carer each have between 1 and 10 (*n = U(1, 10)*) potentially infectious contacts during their travel to the vaccine clinic and in the waiting room.

For each of the potentially infectious contacts by the child and parent with community members, there is a probability of transmission (*to = Ro / NΨ*), which for example corresponds to (*to ~ 6%*) probability of a transmission event occurring for (*Ro = 2.5*) secondary infections for someone with 6 contacts per day during their infectious period of 7 days (i.e., a community member) or 21 potentially infectious contacts per day but who self isolates on symptom onset that occurred 2 days into their infectious period (i.e., a vaccinator).

Both the vaccinated child and the parent or caregiver, will be at additional risk of exposure during travel to the vaccine clinic, while waiting at the vaccine clinic and during vaccination. In addition, we assume that if either of them gets infected they will infect all other household members, owing to the high secondary attack rates observed for family gatherings [6]. We ignore any additional secondary infections outside the household, which are likely to be minimal due to physical distancing measures.

Based of the Reed-Frost epidemic model [7], the probability (*P*) for a SARS-CoV-2 infection for the whole household of a child who gets vaccinated is calculated as one minus the probability of either the infant or the mother not being infected by either the vaccinator or anyone else on any of the vaccination visits: , with *v* the number of vaccine clinic visits. Hence, the probability for such infection to be in excess of SARS-CoV-2 infections that would have occurred otherwise is *PE = P (1 - Θ)*.

We assume that during the 6 months of SARS-CoV-2 transmission, all children who get one dose of DTP will also get the other two doses. However, children receiving their measles containing vaccines will only get one dose during that time window because the two doses are given more than six months apart. The number of children who would normally get DTP during the considered time frame is approximated by half of the under one-year old population. Similarly, the number of children who will get either the first or the second measles-containing vaccine dose is half of the under 1-year old children or half of the children aged 12-23 months respectively.

**Sheet Detailed COVID Calculations - 2**

**Reduced Number of Contacts + Intervention-Specific Reduction in Infection Risk (Columns Z:AC)**

1. **Risk of SARS-Cov2 Transmission**

Cell Z15 = 1-((1-Risk_of_Provider_Being_Infected*Transmission_Risk_Contact_Health_Care_Provider*(1-$I15))^($H15)*(1-Risk_of_Community_Member_being_Infected*Transmission_Risk_in_Community)^($H15*Contacts_During_Travel_to_Facility))

1 - risk of getting infected by health care provider x (1-reduction in transmission risk through mitigation measures)^ (number of visits after mitigation measure) x (1 – risk of getting infected on the way to the clinic by a community member)^ number of visits after mitigation measure x contacts during travel to facility)

1. **Excess Risk**

Cell AA15 = Z15*(1-(Percent_Prevalence_at_End_of_Projection_Period-Percent_Prevalence_at_Beginning_of_Projection_Period))

Risk of SARS-Cov2 Transmission x (1 – percent of population still susceptible to getting infected)

1. **Excess Infections**

Cell AB15 = $F15*$AA15*HH_Size

Additional Number of Patients Receiving Care due to Mitigation Measure x Excess Risk x HH Size

Need to add modification for infection rate in HH

**Sheet Detailed COVID Calculations – 2 – Cells F5-F12: Estimation of Number of visits by package**

Family Planning:

Straightforward summation of users of the different methods (no overlap)

Antenatal Care:

Total number of visits for women who get at least 4 ANC visits

Delivery Care:

Number of facility-based deliveries

Newborn Care:

Number of facility-based deliveries

Breastfeeding:

Number of women who are instructed in early breastfeeding, complementary feeding education only and complementary feeding+ education+x (no overlap between the last two interventions and initiation of BF at different time

Question: Initiation of BF happens at facility delivery, no extra visit – take out?

Vaccines:

All vaccination visits divided by 3 (assumption that child receives on average 3 vaccinations per visits during its first year of life – based on national vaccine schedules)


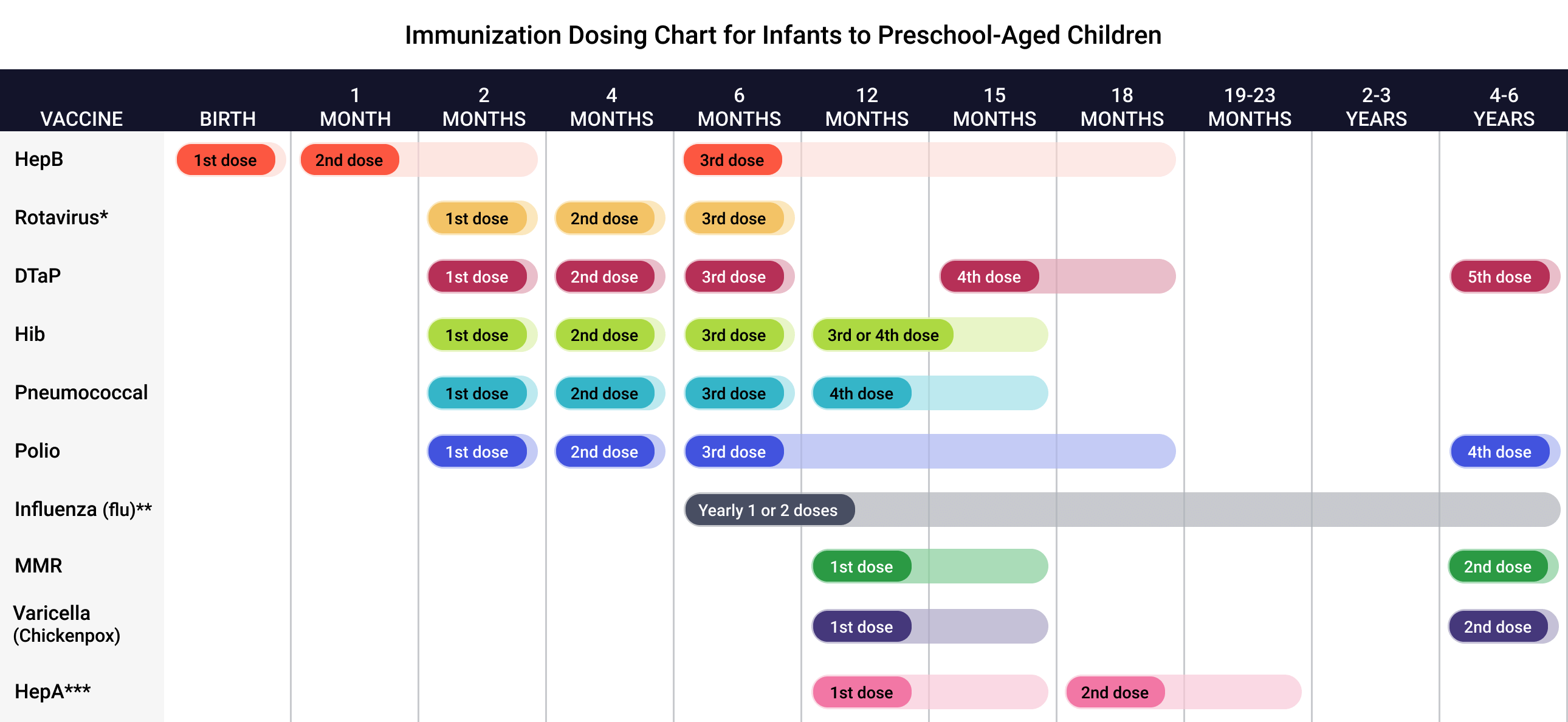


Child Health:

Adds up all child health visits as they are for different interventions (for the 3 diarrhea-related interventions only ORS treatment)

Malnutrition:

Adds up treatment for mild and severe malnutrition (no overlap)
