# Supplementary material for: Benefit-risk analysis of maintaining essential Reproductive, Maternal, Newborn, and Child Health (RMNCH) services against risk of COVID-19 infection
Source: PLOS Glob Public Health. 2022 Feb 9;2(2):e0000176. doi: 10.1371/journal.pgph.0000176 (PMC10021309; doi:10.1371/journal.pgph.0000176)
Supplement: Attachment — Submitted filename: Reviewer Response - Benefit Risk Analysis - Oct 31.docx [file pgph.0000176.s005.docx]

PLOS Global Public Health

Editor-in-Chief

October 29, 2021

Dear Editor-in Chief:

Enclosed is our response to reviewers for the manuscript entitled: Benefit-Risk Analysis of Maintaining Essential Reproductive, Maternal, Newborn, and Child Health (RMNCH) Services Against Risk of Covid-19 Infection. Our responses are in red.

We are extremely grateful to the reviewers for their comments and suggestions and as a result we have made extensively revisions to the manuscript that we feel has dramatically improved the manuscript.

Reviewer #1: Overview:
This interesting article describes the experience of planning and conducing a risk-benefit analysis for essential RMNCH services during the COVID-19 pandemic in select countries in 2020. As the authors describe, the article aims to both describe the technical aspects of the modeling framework used as well as reflect on the experience of conducting this exercise during the pandemic. Achieving both of these important aims in a single manuscript is certainly an ambitious undertaking. Below, I have tried to focus my comments on areas where some additional methodological detail or description might help to strengthen the manuscript’s ability to achieve these interrelated goals.

Major comments:
1. To improve the clarity of the manuscript, reorganization of the introduction and methods would be helpful. As currently written, the manuscript mixes together background information (i.e. a general description of mechanisms for essential health service coverage disruption in lines 95-106, or a description of potential mitigation strategies and their impact in lines 116-122) with more detailed methodological description that is specific to the analysis presented in this manuscript. I think that the manuscript would be clearer if some of this information were to be moved to the introduction section, so that the introduction contains the background information that sets the stage for the risk-benefit analysis introduced here, and the methods section clearly describes what was done for this particular analysis.

We totally agree and have rearranged the manuscript extensively, we have pulled the recommended sections into the introduction as suggested.

2. Within the methods, a clearer description of the modeling/analytic framework at an earlier stage in the methods section would be helpful. Currently, this information is somewhat scattered throughout the methods. For instance, there is a brief mention of LiST on lines 132-135, a list of key model inputs and other pieces of the model on lines 154-161, and then a very brief description of an Excel model and LiST interacting in lines 216-221.

I might suggest that providing a high-level overview of the modeling framework early in the methods section would be helpful, perhaps accompanied by a modeling flowchart and/or analytic diagram that shows the key inputs into the model as well as more detail about the modeling steps (i.e. what is done by the Excel tool and what is done by LiST). This would help to orient the reader in preparation for more detailed discussions about the data and assumptions used to inform the model later on.

We agree and have rearranged the methods. The methods now start with an overview, the analytic framework, the modelling approach and description of the models used, the data inputs, calculations and then followed by country application. To better understand the methods we have also have added an analytic diagram (figure 2 Model Flow Chart) that shows the key inputs and processes as was suggested.

Last, I think that the description of the Excel model and its interactions with the LiST model (lines 216-221) could benefit from some expanded detail. In particular, it would be useful to be specific about the calculations used here for the coverage levels under different intervention scenarios, and what the lives lost and saved from LiST represent (i.e. what time period was evaluated – just 2020, or 2020-2021, or beyond). From the relatively brief description given in the methods section, it is difficult to understand exactly how all of the various data inputs and assumptions were synthesized to produce the final results presented here.

We have expanded extensively on this to clarify including the time period, the analytic diagram in (Figures 2 Model Flowchart) we believe makes is easier to understand. We have also provided in the annexes (supplementary files), instruction guide, mitigation assumptions by country, details of model calculations. Finally we would like to draw your attention that since this article was submitted we have released a document that details the model structures, strengths and weakness, of models used to assess the indirect impact of COVID-19 on SRMNCAH and Nutrition Services which includes a detailed review of the two models we used for this analysis. This document is now referenced and can be found here https://www.who.int/publications/i/item/9789240027695

3. It would be helpful to more clearly describe how several of the input data estimates were derived. My sense from reading the article is that the country teams made the ultimate decisions about which input data to use, though guided by expert opinion / recommendations, specific quantitative analyses performed for this exercise (i.e. of service disruption magnitude), or other sources (i.e. Spectrum). In some sections of the methods, however, this process is not entirely clear.
a. For the baseline data section (lines 163-164), this is clearly laid out – each country reviewed data from Spectrum but had the option to use updated or better-quality data at their discretion.
b. For the disruption data section, there is a description of a quantitative analysis that was used to estimate disruptions (lines 171-183). It seems, however, that there was also some ability for countries to make individual decisions about the best way to assess service disruptions in their country (i.e. lines 183-184). It would be helpful to better understand how country expertise and this quantitative analysis were combined to reach final estimates of coverage reduction. (I have several technical questions about the quantitative analysis used to inform disruption estimates in a separate comment below).

More detail is added to the manuscript to clarify these points but please note we first now describe the modelling and analytic approach and then in more detail what was done with the country application

Specifically noted in the overview of methods lines 162 to 163 we state

Coverage disruption caused by the COVID pandemic was estimated through an analysis of countries’ monthly health service statistics//Health Management Information System (HMIS) data.

For the country application lines 216 to 235 describe in detail what countries inputted.

“For the assessment of disruption, countries were asked to gather monthly health service utilization data for 2019 and 2020 for RMNCH services. This was based on WHO guidance for using routine health information for analyzing health service disruption.22 As data collection was completed in early 2021 and updated as late as March 2021, all countries were able to gather data from Jan 2019 through December 2020. Recognizing that some HMIS systems may have data quality issues, it was assumed that the data quality issues would be consistent across the years, and it was thus considered possible to calculate relative reductions in service delivery. Most countries saw coverages declines due to the pandemic starting in April of 2020, with disruption continuing over the next three to six months, after which most countries observed some recovery (probably due to a mix of a slowing down of the spread of SARS-CoV-2 as a result of general mitigation measures such as lockdowns and mask mandates as well as specific mitigation measures taken to restore health service coverage). To estimate the disruption caused by COVID, we only included in the analysis those months in which coverage levels were below pre-pandemic levels (the affected months in 2020 were compared to the same months in the year 2019 to account for seasonal variations in disease patterns and health service use). Key indicators (e.g. % of women receiving four or more antenatal care visits, or % of women delivering with a skilled birth attendant) were used to estimate service disruption and then applied to the respective package (i.e., family planning, antenatal care, delivery care, etc) (Table 1). If a country had HMIS data for more than one indicator in a package (i.e., the country had data for institutional delivery and C-section delivery), disruption was calculated based on the average disruption seen across those indicators.”

c. For the mitigation strategies (lines 192-213), there is a statement that “potential impacts of these mitigation measures were evaluated with a view to [three potential impacts on health outcomes]”. In Annex 2, I see suggested and country-specific impact estimates for the various mitigation measures. It would be helpful for the authors to explain how this process worked – i.e. who came up with the “suggested” estimates and how, and how were these suggested estimates related to the final country-specific impact estimates? Based on the discussion section, my guess is that there was some sort of expert consensus process used for the suggested estimates, and then country teams could choose to adopt or modify those based on their own assumptions. Still, it would be helpful to more explicitly describe this process in the methods section.

More details have been provided. In the general overview of methods we specify first line 164 to 178 how mitigation measures were considered and this was in the initial manuscript submitted

“Mitigation measures to maintain essential health services were initially taken from the WHO interim guidelines on maintaining essential health services during the pandemic.12

The strategies were sorted into several categories (S1):

1.	Hygiene and Distancing Measures

2.	Measures affecting number of visits required to provide care (bundling of visits, provision of several months of supplements or contraceptives during one visit)

3.	Measures shifting health care to community providers, tele-health or outreach teams

4.	Measures strengthening health worker capacity (increasing availability of providers and training in transmission prevention)

5.	Measures to strengthen the supply chain of commodities and medical supplies

6.	Measures to promote guidelines and messaging specific to the context of SARS-CoV-2

This list was supplemented by strategies developed by countries that participated in the field-testing phase. Each mitigation strategy was then judged on the impact it might have on restoring coverage of the intervention it was targeting (e.g., antenatal care, immunization) based on consultation with country experts and the available literature at that time.”

In the country application portion of the manuscript we now explain in greater detail how mitigation measures were assessed lines 246 to 262

“For identification of mitigation strategies and their impact on health service coverage rates, as previously described, each country team collated information on what mitigation activities were taking place and each mitigation strategy was judged on the impact it might have on restoring coverage based on the limited scientific evidence available on what might work in health settings,17-21 as well as expert opinion. Some mitigation measures were estimated to improve coverage due to the possibility that the mitigation measure would increase patients’ confidence that a visit to a health facility would be safe, such as visible hygiene and social distancing measures at health facilities or education campaigns reassuring the population that it was safe to visit health facilities and stressing the importance of continued care seeking, even during the pandemic. Other mitigation measures were thought to increase health care coverage by increasing availability of health work force (by involving community-level health workers or task shifting) and ensuring a reliable supply chain. Due to the challenges of calculating the impact of mitigation measures, a concerted effort was made to keep the impact estimates for individual mitigation measures conservative and the model included control features to ensure that overall coverage assumptions stayed within a defined range (e.g., coverage after mitigation measures were implemented was not allowed to be more than 2% above pre-pandemic coverage levels). S1 provides an overview over the most common mitigation strategies and estimates of their impact by country. ”

4. Lines 177-180: The approach that the authors take to estimate disruptions due to COVID for essential health services generally seems reasonable, although I have a few specific questions about the approach used here.

a. First, were there any months in 2019 for which there were unusual fluctuations in service provision in the countries in question (i.e. due to shortages or stockouts of equipment or supplies, health worker strikes, etc.?). If so, were those months additionally excluded from the analysis?

No countries mentioned any of these complications, so no special adjustments were made

b. Second, did the authors account for expected changes in any indicators between 2019 and 2020 due to, e.g., changes in population size? If a country experiencing population growth, for instance, simply providing the same number of ANC visits, etc. may represent a decrease in the proportion of women receiving care. If any adjustments were made, it would probably be worth mentioning those here; if not, perhaps the authors could mention if/why this effect was thought to be negligible.

Population growth was taken into account, this is the normal procedures used in SPECTRUM, LIST and we have provided some more references to these models as well as including an new reference of a recently released document by WHO that contains a detail review of these models and their use to measure the indirect impact of COVID-19 pandemic on SRMNCAH. Please note that are using coverage rates, not numbers of women/children covered.

c. Were the authors able to assess whether any of the observed changes in service delivery during the pandemic may have resulted from either delayed or incomplete reporting in 2020 compared to 2019? Especially during the early months of the pandemic, one might expect that the same factors that disrupted health services more generally may have made reliable and timely reporting more difficult. From some analyses of vaccine coverage data collected throughout the pandemic, it seems that monthly data reported by many countries was subsequently revised (usually upwards) in later reports, as might be expected with ongoing data collection (especially during the pandemic). Perhaps, however, the HMIS data also contains ancillary data quality indicators that suggested that delayed and/or incomplete reporting wasn’t an issue in 2020. In either case, it may be worth a brief note in the manuscript to address this potential question.

Yes that is a good point, we have added the following lines 187 to 189

“As data collection were completed in early 2021 and updated as late as March 2021, all countries were able to gather data to December 2020 thus potential issues related to delayed or incomplete reporting were lessoned.”

d. For some services – i.e. vaccination – large disruptions early in the pandemic could be at least partially mitigated by concerted catch-up services later in the pandemic. Was this possibility taken into account? Or did the strategy of only using months for which disruptions were reported limit the model’s ability to account for catch-up vaccination, for instance?

Please note that we only used deaths in the months for which disruptions were reported and only did projections for one year. There were constraints related to LiST that we explain below. But we should note that catch up services likely would have greater impact if our projections were based on more than one year. Also see below our response in regard to vaccinations.

e. Last, the authors write that they considered only months for which there were observed disruptions. I’m not quite sure how that translates into the disruption estimates that are later presented in Figure 4. Does this mean that a country seeing a 50% reduction in services in March 2020 and 30% reduction in services in April 2020 – but no subsequent disruptions after April – would be assigned a 40% reduction? Or would this be averaged out with “no-disruption” months from May-December to produce an estimate from March – December 2020 (i.e. an 8% reduction in total between March – December 2020)? Some clarification around how to interpret these indicators would be helpful. For instance in Figure 4, there is a table that presents a comparison of the level of service disruption for various indicators. What does what “COVID-19 impact on coverage, by country” means in this table? Is this the impact on coverage for the full year of 2020 (i.e. including January and February 2020)? For just the pandemic months (i.e. March – December)? Or for just the months in which there were reported service disruptions?

It is an average of the months with disruption. The LiST only calculations disruptions by year so we had to assume that a disruption of several months translates into one full year. Yes, it is true that recovery back to normal can occur but we also assumed that services returning to normal for a few months would not be sufficient in that time period to make up for the disrupted services earlier. To be clear we added also to the title to Table 2 “for the year 2020” which was figure 4

This limitation of LiST that required us to apply several months of disruption to an entire year is outlined on line 346 and 349

“The annual nature of the LiST model also imposed some limitations on the analysis, as it was not possible to enter disruption rates by month, an average disruption rate had to be used and applied to the entire year.”

5. In the discussion, the authors reflect on the experience of country teams in using this tool to conduct a risk-benefit analysis. For instance, on lines 288-301, the authors describe that the exercise was manageable and produce what seemed like reasonable response, and that ongoing learning throughout the process was helpful to countries that joined the exercise later. It would be helpful, however, to also know whether or not the exercise was used to guide any policy decisions. If so, what sorts of decisions were informed; if not, what barriers may have limited the perceived utility of these results or their uptake?

The work was done with countries and presented various parts of the ministries of health (usually their COVID-19 task force) and other partners in countries. It is hard to quantify the impact as this was a rapidly moving epidemic with many changes. We do know that there was a lot of advocacy done to maintain these services and now some evidence that services are coming back but hard to correlate directly to this work.

But to clarify we added the following lines 325 to 329

“The results were available through country reports and were presented to ministries of health and other in country health partners. However, since this model was being created and tested while urgent decisions about essential health services had to be made it was not possible to use this information in real time, but future applications of a refined tool for not only COVID-19 pandemic but other pandemics and emergencies should make such decision making possible.”

Minor comments:
1. I may have missed this, but what was the time frame that countries were exploring during this analysis? I see that the field testing took place from September – December 2020. Were the country teams analyzing what was currently happening (i.e. in 2020), or were they looking forward (i.e. 2021 and/or additional years afterwards?)

The study projected deaths for the year 2021. What would happen if no mitigation was applied (assuming that coverage rates would be depressed just as they were in 2020 when Covid-19 pandemic hit but no mitigation measures were yet implemented) compared to if mitigation measures were implemented

The tool was created from August to September 2020 and the field testing actually took place November extended to March 2021 but with a lot of preparation taking place in October, so we clarified those dates throughout the paper.

2. Lines 89-93: The authors state that the Excel-based risk-benefit analysis “identifies the most effective mitigation interventions and strategies”. I see in the results that the authors discuss which interventions / health service packages were most influential in saving lives in this modeling exercise, but I don’t actually see an analysis of which *mitigation* strategies were most effective (e.g. if temperature screening is more effective than social distancing or vice versa). Is this a misstatement, or have I misinterpreted the analyses presented here?

We changed that sentence to read (lines 111 to 125)

“The Excel-based Benefit-Risk analysis developed for this modelling exercise compares lives saved through the continued provision of essential health services with lives lost due to SARS-CoV-2 infections acquired accessing these services, and identifies the most effective interventions (i.e., those that save the most lives.) assuming mitigation strategies succeed to maintain coverage of those interventions.”

3. Figure 3: For some of these indicators, it seems possible that the demand for the health service may have declined during the pandemic as well. (I am thinking here specifically of the child health indicators, especially antibiotics for ARI – one might reasonably hypothesize that the same mitigation measures put into place to limit the spread of COVID would also decrease transmission of respiratory infections. The same might also be true for diarrhea via school closures, social distancing, etc., though perhaps to a smaller degree.) It seems that assessing this complex interplay between demand and service position is probably beyond the scope of this study, but I wonder if the authors could comment on whether this is a limitation for the present study and/or if this was taken into account when these disruptions were entered into LiST.

Yes we absolutely agree. It is not possible to take this into account in LiST unless we just removed those disruptions from the analysis. Instead, we have added the above suggestions to the limitation section

Lines 352 to 358

“Not considered in the interpretation of the findings, was the possibility that there might have been a decrease in the need for health services during the pandemic as well. This applies specifically to child health indicators, especially antibiotics for ARI – one might reasonably hypothesize that the same mitigation measures put into place to limit the spread of COVID would also decrease transmission of respiratory infections. The same might also be true for diarrhea via school closures, social distancing, etc., though perhaps to a smaller degree. Assessing this complex interplay between demand and service position was beyond the scope of this study.”

4. Some of the reported disruptions in Figure 4 are quite a bit larger than other data sources suggest. For instance, comparing the recently released WUENIC estimates and data to Figure 4 for vaccination coverage (here using DTP3 from WUENIC from <https://immunizationdata.who.int/pages/coverage/dtp.html?CODE=AFG+MAR+IRQ+PAK+SOM+SDN&ANTIGEN=DTPCV3&YEAR=>), AFG reported 86.7% coverage in 2019 (WUENIC estimate: 72%) and 85.3% in 2020 (WUENIC estimate: 70%). Both the WUENIC estimates and official country-reported data therefore suggest a much smaller disruption than the 18% disruption indicated here. Do the authors have any insight into the reason for these differences? (I appreciate that this is quite new data and that it’s not clear what the true level of disruption was over the course of 2020).

Yes, this is true that is was larger than the subsequent WEUNIC estimates, which is good. As we mentioned before it was based on in country experts and official first data. So yes, it is due to the earlier data, and subsequent catch up by the immunization program. However, it reflects the evidence available at the time this done. We are working on re doing this type of modeling for essential services and those additional coverage estimates will be added. It should also be noted that subsequent surveys and other data are supporting less disruption than originally thought. However, since we did lives saved projections only out to one year, as opposed to many years, we don’t feel we are too far off.

We now added to limitations line 342 to 345

“Since this modelling exercise there is some evidence, for example regarding immunization, that service disruption may have recovered more quickly than anticipated.^47^ However, this should not significantly impact our findings as we assumed only one year of disruption and that herd immunity would provide protection.

5. Lines 285-287: “The annual nature of the LiST model also imposed some limitations on the analysis.” Could the authors provide a bit more detail about what these limitations might be and how they should affect the reader’s interpretation of the results?

The main problem was that we could not enter disruption data by month, but had to calculate an average for the 6-month disruption period that was then applied to the entire year.

We have specified that in limitations line 346 to 349

“This benefit-risk analysis used the LiST model in a new way, as LiST was originally designed to model the impact of gradual coverage scale-ups, not short-term disruptions. The annual nature of the LiST model also imposed some limitations on the analysis, as it was not possible to enter disruption rates by month, an average disruption rate had to be used and applied to the entire year.”

6. Line 212 refers to Annex 3 – should this be Annex 2? In Annex 2, I’m also not sure how to interpret the rows in which only some countries have their own estimated effect sizes. For instance, “Implement a requirement that patients bring their own mask” has only a suggested effect size but no country-specific effect sizes. Does this mean that no countries implemented this measure? If so, I would suggest adding a note to the table to make this clear (i.e. impact estimates are only given for countries that included this strategy as part of their mitigation efforts).

Added, please note some figures were changes to Tables and not supplementary files are listed at the end of the document

Reviewer #2: The submitted manuscript, Risk-Benefit Analysis of Maintaining Essential Reproductive, Maternal, Newborn, and Child Health (RMNCH) Services Against Risk of COVID-19 Infection provides insights into one approach countries have taken in assessing the trade-offs between death due to the novel coronavirus infections and essential health services, with the support of the World Health Organization. For this reason, this reviewer finds this manuscript of value for the global health community; particularly, of interest to those potential users of such a tool.

However, the current state of the manuscript leaves the reader with major questions mostly due to poorly explained figures and the lack of supporting evidence supposedly available in an annex, but was not provided in this review. While the authors list having adapted the work of Roberton et al. and Abbas et al. few to no clear assumptions or sensitivity analyses were provided.

Please note we agree we should have done a better job describing everything and we feel this major reason does just that.

Major comments:
- Figure 1 in this manuscript is not sourced from the original authors (Roberton et al.) and no indication is provided if the current authors were given rights to republish.

Permission granted

- In general, figures are not adequately captioned or explained in the manuscript. As an example, Figure 1, Figure 2, nor Figure 4 are not mentioned in the manuscript and lack captions for the readers to understand/interpret their importance.

We fixed this

- Citations are lacking in the entire introduction, though many statements warrant references.

Multiple references have been added

- Annex 2 is references in the manuscript as containing the "detailed explanation of the variable and formulas used" however, Annex 2 seems to be what is referred to as Annex 3 in the manuscript - "an overview over the most common mitigation strategies and estimates of their impact"
- Annex 2 should be included
- Current "Annex 2" should be relabeled as Annex 3

We fixed that and all six country models, were uploaded to a publicly accessible folder for anyone interested in the variables and formulas used. We have fixed the Annexes (supplementary documents) and also, we note that we now have additional supplementary files for the tools and have listed them as supplementary files for the paper

- Annex 1 in the manuscript is mentioned to have 72 interventions, however the available Annex 1 only contains 69. This should be remedied.

Fixed, please note we have changed Annex 1 to Table 1 instead

- The limitations of the approach taken is not well addressed as the assumptions underlying the analyses are lacking in the main manuscript.

We have done an extensive rewriting of the manuscript that should clear most of this confusion. This adds extensively to the length of the manuscript. There were too many assumptions to include in this article but all country-specific data are made available online in figshare (6 country models, etc.).

- Were any sensitivity analyses conducted by country stakeholders? It would be interesting to understand how changes in an assumption or the underlying data might lead to variations in outcomes.

Sensitivity analysis was mainly conducted to prevent unrealistic results (such as coverage increase vastly beyond coverage levels of previous years, etc.). In particular, sensitivity analysis was used to finetune countries’ assumptions about the effectiveness of mitigation interventions in consultations with the countries. A formal training of countries in sensitivity analysis was beyond the scope of this exercise.

- Manuscript could be strengthened by clearly stating how policy makers and researchers could access this tool and leverage it to adjust for the changing dynamics of the coronaviruses (detla, gamma, etc.)

All the instructions, data entry sheet and formulas used are now available as supplementary documents to the public. The data entry sheet has formulas embedded that researchers can use or change.

Minor comments:
For clarity, this reviewer recommends that the authors use either "risk-benefit" or "benefit-risk" and not interchange these as readers may be confused. This is found in both the manuscript text and figures.

Changed throughout to benefit risk analysis as we are presenting benefit-risk ratios (lives saved compared to lives lost) so everything is consistent
