# Supplementary material for: Benefit-risk analysis of maintaining essential Reproductive, Maternal, Newborn, and Child Health (RMNCH) services against risk of COVID-19 infection
Source: PLOS Glob Public Health. 2022 Feb 9;2(2):e0000176. doi: 10.1371/journal.pgph.0000176 (PMC10021309; doi:10.1371/journal.pgph.0000176)
Supplement: Attachment — Submitted filename: PGPH-D-21-00183_R1_reviewer_response_final.docx [file pgph.0000176.s006.docx]

# Dear Editor,

We would like to thank you for considering the manuscript and for the helpful reviews. Here are the answers to the reviewers and we attach a revised manuscript.

# Submission version: First revision

# Recommendation: Minor Revision

# Comments to Author:

Manuscript reference number: PGPH-D-21-00183

Title: Risk-Benefit Analysis of Maintaining Essential Reproductive, Maternal, Newborn, and Child Health (RMNCH) Services Against Risk of Covid-19 Infection

Authors: Weissman *et al*.

**Overview:** Overall, the reorganization and re-writing undertaken by the authors has greatly strengthened the manuscript. I now find it quite easy to follow and understand, and the strengths and limitations are well laid-out. I have a few minor follow-up comments below, but no additional new major comments.

**Previous major comments:**

*Previous major comment #1 (regarding the organization of introduction and methods):* The substantial changes made by the authors have dramatically improved the organization and clarity of the manuscript. The revised structure and content is extremely clear and easy to follow. I greatly appreciate the authors’ excellent efforts in this regard and have no further comments about this topic.

*Previous major comment #2, regarding the description of the modeling/analytic framework:* As with the previous major comment #1, the reorganization and the re-writing of the manuscript has greatly clarified the modeling/analytic approach, and also helped to separate this from the field testing. The provided modeling flowchart is also much appreciated. Separating out the descriptions of the LiST and Excel models is also extremely helpful, and it’s much easier to understand how the two models work together in this revised manuscript. The provided references to model descriptions are also helpful. I find the manuscript now very easy to follow and much stronger as a result.

My one remaining comment here is that it would be helpful to explicitly clarify what was the time horizon used in the modeling exercise. In a response to one of my later comments, the authors say that these projections were made for 2021, but I don’t actually see that specified in the manuscript. Simply adding in the description of the methods that the LiST and Excel models were used to estimate deaths under various scenarios for 2021 (if that’s correct?) would be helpful. Some of the disruptions to EHS studied here (e.g. vaccination) could potentially have longer-term effects (beyond 2021) if not corrected, so it would be helpful for readers to understand specifically that the country teams were estimating relatively near-term benefits and risks. Otherwise, no further questions / comments about this topic.

**Response**

**THE LIST AND EXCEL MODELS WERE USED TO ESTIMATE DEATHS FOR SIX MONTHS OF 2020 (MARCH TO AUGUST 2020), WHICH WERE THE MONTHS WHERE WE SAW THE GREATEST DISRUPTION OF SERVICES IN THE CONTRIES INCLUDED. ALTHOUGH ESTIMATES COULD BE MADE FOR 2021 IN THE PAPER WE REPORT THE ESTIMATES FOR 2020.**

**We added the following line 162**

**“Due to the annual nature of the LiST model it was not possible to enter disruption rates by month, thus for application in countries (see below) an average disruption rate based on the 6 months (March to August 2020) during which services were disrupted was applied to the entire year. This was done for two reasons. One was to exclude the months prior to the pandemic. Two was to exclude the latter months of the year in which mitigation measures may have been in place, so that we estimate what 2020 might have looked like had no mitigation measures been implemented.”**

**Also in line 230 we added average disruption and the analysis months used**

**To estimate the average disruption caused by COVID, we only included in the analysis those months (March to August 2020) in which coverage levels were below pre-pandemic levels (the affected months in 2020 were compared to the same months in the year 2019 to account for seasonal variations in disease patterns and health service use).**

**On Table 2 title we added the months used and reworded it to read**

**“Estimated average coverage disruptions from March to August 2020 applied to the year of 2020 by service package by country and included in the analysis”**

**Also in line 185 we added to clarify the year of the projections**

**Baseline coverage and reduced coverage levels were entered into the LiST model to calculate lives lost due to pandemic-induced coverage reductions for 2020.**

**Finally we would like to point out the LiST takes into account herd immunity. Also please note on line 295 we state that**

**“Vaccinations also had lower benefit-risk ratios, mainly due to the fact that the model assumed that vaccination campaigns would only be disrupted for one year, during which prior vaccinations and herd immunity would continue to provide protection. However, if immunization campaigns are delayed for longer than one-year, deaths would increase significantly in the future.”**

*Previous major comment #3 (regarding input data sources and estimates*): As the authors note, the reorganization to first describe the modeling / analytic approach, then to focus on the country application is extremely helpful. In particular, the description of how countries assessed coverage declines from HMIS data – as well as how the mitigation strategies and their impacts – is now much clearer, and the primary role of the country teams in this process is now more apparent. Previously, it was unclear to me how exactly the quantitative estimates of impact for mitigation measures were obtained, but now the use of (limited) evidence with expert opinion is more explicitly explained. More generally, the manuscript now clearly explains how the modeling tool was derived, and separates that from how the countries applied the tool, which helps to clarify some of these decisions. I appreciate these changes and have no further comments in this area.

**Response**

**Thank you again your suggestions were most helpful**

*Previous major comment #4: regarding the estimation of disruptions due to COVID for various essential health services.* In general, the reorganization of the manuscript has helped with this area substantially, and the authors now explain more clearly that HMIS data were used were available to calculate these disruptions. I appreciate the authors’ notes that there were not any known major disruptions to services in 2019 which would affect the 2019/2020 comparisons used here (previous major comment 4a), and appreciate the clarification that coverage rates were used rather than counts when calculating disruptions from HMIS data, which helps to mitigate (at least partially) challenges in these calculations related to population changes over time (previous major comment 4b). The addition to lines 187-189 is also helpful re: delayed / incomplete reporting (previous major comment 4c), as the March 2021 data collection date is likely to minimize issues with delayed reporting and perhaps with incomplete reporting as well. I appreciate that the authors have highlighted this as a potential limitation, however.

I still have some questions about my previous major comment 4e. I appreciate that LiST is not able to take in monthly disruption rates, so the authors had to calculate an average disruption rate and apply this to the entire year. My question, however, relates to *how* this average disruption is calculated. If I understand the authors’ reply correctly, however, they only used the months in which a disruption to EHS had occurred to calculate this average, however. This is also outlined on lines 226-229.

**Response**

**COVERAGE DISRUPTIONS WERE CALCULATED FROM COUNTRY HMIS OR DHIS DATA. ALL COUNTRIES PROVIDED DATA FROM THEIR HMIS/DHIS SYSTEMS FOR THE INDICATORS THAT THEY HAD. FOR EXAMPLE, MOST COUNTRIES HAD NUMBERS OF BIRTHS IN MARCH, APRIL…ETC. OF 2019 AND 2020. WE CALCULATED THE DIFFEREENCE. FROM THAT WE CALCULATED A PERCENTAGE REDUCTION IN COVERAGE THAT WE APPLIED TO THE POPULATION IN 2020 SO POPULATION GROWTH WAS INCLUDED. WE ALSO RECOGNIZED THAT SOME COUNTRIES MIGHT HAVE INCOMPLETE HIMS DATA OR THAT THERE MAY BE PROBLEMS WITH DATA ACCURACY. HOWEVER, WE RATIONALIZED THAT DATA ISSUES WOULD PROBABLY BE SIMILAR FOR 2019 AND 2020 AND HENCE THE DISRUPTIONS WERE STILL REFLECTIVE OF RECUCED SERVICE PROVISION.**

I agree with the authors that it’s unlikely that “services returning to normal for a few months” would be “sufficient in that time period to make up for the disrupted services earlier”. But that doesn’t explain why one wouldn’t average all of the months together to calculate the full-year 2020 disruption to essential health services.

For instance, say that ANC services were disrupted in a hypothetical countries as follows:

January 2020: no disruption (0%)
February 2020: no disruption (0%)
March 2020: 15% disruption
April 2020: 30% disruption
May 2020: 25% disruption
June 2020: 10% disruption
July 2020: 5% disruption
August 2020: no disruption (0%)
September 2020: no disruption (0%)
October 2020: no disruption (0%)
November 2020: no disruption (0%)
December 2020: no disruption (0%)

If the authors / country teams had data from the full year from HMIS systems – which is my understanding, based on the notes that some of the data were extracted as late as March 2021 – then it seems like the best way to estimate coverage disruptions due to COVID for the full year of 2020 would be to average all of the months together, including the disruptions in the “no-disruptions” month, which would give about a 10.8% disruption for the full year. If I understand the methods presented here, however, the country teams in this analysis would only have calculated the average disruption using data from March-July, which would give about a 21.7% disruption. The latter approach seems likely to substantially overestimate the true “full-year” disruptions to services, if there are any months in which no disruptions were observed. It seems inaccurate, then, to call this an estimate of coverage disruptions “for the year of 2020” as in line 239 / Table 2 caption, as these are likely to be an overestimate of the true full-year disruptions suggested by the HMIS data.

**Response**

**PLEASE SEE OUR ABOVE FIRST RESPONSE WHERE WE MADE CHANGES IN THE MANUSCRIPT TO ADDRESS THIS**

**NOTE THAT THE GOAL WAS TO SHOW THE IMPACT OF COVID IF NO MITIGATION MEASURES WERE TAKEN COMPARED TO WITH MITIGATION MEASURES. INCLUDING MONTHS IN WHICH COVID DIDN’T EVEN EXIST (JAN – MARCH) WOULD HAVE MADE NOT MUCH SENSE. WE EXCLUDED THE RECOVERED COVERAGE RATES THAT MOST COUNTRIES SAW IN THE FALL OF 2020 AS THOSE WERE VERY LIKELY DUE TO MITIGATION MEASURES TAKEN BY THE COUNTRIES BECAUSE WHAT WE WERE INTERESTED IN WERE COVERAGE RATES BEFORE COUNTRIES ACTUALLY TOOK MAJOR MEASURES. THE POINT OF THE EXERCISE WAS TO ESTIMATE HOW MANY LIVES WERE LOST AND SAVED AND THE RISK BENEFIT RATIO THAT RESULTED FROM THE LOCKDOWNS AND DISRUPTED SERVICES. IF WE HAD INCLUDED ALL THE MONTHS WHERE THERE WAS 0 DISRUPTION (OR EVEN IN SOME CASES INCREASED SERVICE PROVISION) OUR RESULTS WOULD BE WATERED DOWN AND NOT DEMONSTRATE THE IMPACT OF DISRUPTED SERVICES. WHILE THE EXCEL SHEET CALCULATED LIVES LOST AND SAVED ETC FOR THE WHOLE YEAR BASED ON THE PROPORTION OF DISRUPTION WITNESSED FOR THE SIX MONTHS USED IN THE CALCULATIONS, THE FULL YEAR OF DISRUPTION DATA WAS THEN DIVIDED BY HALF TO DEMONSTRATE LIVES LOST OR SAVED DURING THE SIX MONTHS THAT SERVICES WERE DISRUPTED.**

**TO CLARIFY FURTHER WE ADDED THE FOLLOWING ON LINE 187**

**“As all countries saw service disruptions for only half of the year, and LiST only calculates lives lost for the whole year, lives lost results generated from LiST for this model were divided in half to calculate lives lost for the six months of service disruption.”**

I am guessing that the authors may have a reason for this approach, however. For instance, was the intent to try to create a counterfactual / **hypothetical estimate of what disruptions might have looked like without any mitigation measures (that is, what 2020 might have looked like had no mitigation measures been implemented during the latter months of the year** I think that this may have been the intent of this approach, based on the authors’ reply to my minor comment #1 below. If so, then it would be useful to explicitly state this in the manuscript, perhaps around lines 226-228 (“To estimate the disruption caused by COVID, we only included in the analysis those months in which coverage levels were below pre-pandemic levels”).

**Response**

**YES, THIS IS EXACTLY THE CASE. WE HOPE THE CHANGES WE MADE IN THE MANUSCRIPT WILL SUFFICE.**

1. *Previous Major comment #5 (regarding policy use / uptake of these results)*: I appreciate the authors’ addition of text describing the use & challenges associated with real-time implementation of this strategy – no further comments on this topic.

**Response**

**Thank You**

**Previous minor comments:**

1. *Previous minor comment #1 (time frame of analysis):* I appreciate the authors’ clarifications – in their reply, the authors write that “The study projected deaths for the year 2021. What would happen if no mitigation was applied (assuming that coverage rates would be depressed just as they were in 2020 when Covid-19 pandemic hit but no mitigation measures were yet implemented) compared to if mitigation measures were implemented.” Both of these points are extremely helpful in understanding this analysis. As described above, I would suggest that the authors make these explicitly clear in the manuscript as well!

**Response**

**In addition to adding 2020 to the methods we also changed the title of Figure 3 to read**

**“Figure 3: Estimated Lives saved as a result of mitigation measures and restored coverage of RMNCH services and Benefit-Risk Ratios, by country for 2020”**

2. *Previous minor comment #2 (clarifying the “most effective mitigation interventions”)*: the new text clarifies this nicely; no further questions on this topic.
3. *Previous minor comment #3 (regarding demand for health services changing during the pandemic*): the new text clarifies this point as well; no further questions on this topic.
4. *Previous minor comment #4 (regarding the magnitude of some of the reported disruptions)*: I appreciate the added text and limitations. I think that some of this may also be related to the intent of this analysis and how the disruptions were calculated (see relevant major comments above) – if the intent is to reflect how big disruptions might have been had no mitigation happened, then the fact that estimated disruptions here don’t fully capture the degree of recovery to services later in the year would be less of a concern. No further questions on this topic.
5. *Previous minor comment #5 (regarding the limitations of the LiST model*): I appreciate the added text; no further questions/comments here.
6. *Previous minor comment #6:* The added note and the rearrangement of the manuscript’s organization in general helps to clarify the interpretation of Supplementary Table 1, thank you. No further comments here.

Second reviewer

Major revisions

**Response**

**Please note we communicated with the editors of your journal about these comments. We are responding only to those comments highlighted by your editor. We want to note emphatically that this was not an academic exercise but rather a practical exercise responding to requests from countries to compare the risk of exposure to benefits of services and not just modelling of indirect of COVID-19 on SRMNCAH as just about all, except some immunization modelling, had already done. Also the country participation and decision on parameters used was key to this exercise. Finally keep in mind when this work was done earlier in the epidemic.**

1. Unclear in terms of the elements of the model used for assessing net benefits. For example it isn't clear how the risks to women and children (and adolescents) were estimated in these countries in relation to the stage of the pandemic, available information on cases, hospitalizations and deaths all disaggregated by gender, geography and stage of the pandemic (read in relation to known variants like Delta). How was this estimated by country? we know that some data was averaged over 6 months for LiST but COVID risks, burden and severity is a much more dynamic process

**Response**

**Please see the response to reviewer one about averaging and using 6 months. In regard to the risks we used all available COVID-19 surveillance information provided by each country at the time of the application. The components assessed and formulas used and calculations are provided in detail in S4 pages 9 to 11. Please keep in mind this was all in the beginning of the pandemic, when there was hardly any data and no Delta Virus. The London School model was the best model out there at the time. We used the exact methods as was done by LSTMH for their vaccination risk modelling that is also provided in detail in S4, but instead applied it to three types of visits**

**1.	Child ambulatory health facility visit (child + caretaker)**

**2.	Adult ambulatory health facility visit**

**3.	Adult hospital stay a clinic visit.**

**Each country had to put in some information on their epidemic at the time of the application**

**Specifically,** w**e adjusted risk of contagion and mortality by current prevalence in the country at that time, R naught, average household size and age-distribution. See table below which can be found in S2 user guide**

**We know this is a rapidly changing epidemic but we used the best information we had at the time of this application and we feel we have provided extreme details explaining all assumptions and formulas. It was impossible and impractical to constantly change assumptions**

2. I did not see any adjustment of exposures based on any country specific stringency data which adjusted risks accordingly or defended not doing so.

**Response**

**We used the information from the experts in the country, as this was a dynamic rapidly changing pandemic, we would have to had change the stringency data frequently and instead we used what was given to us by the country. THIS PROCESS WAS VERY POSITIVE and allowed the country to provide theIR OWN parameters. We felt the actual person on the ground would know best. WE FEEL THIS RESULTED IN INCREASED ENGAGEMENT AND A MORE inclusive process.**

3. input data for effectiveness of various mitigation strategies (p 13 lines 212-213 and S3, page 4). These estimates appear to be entirely based on opinion rather than evidence and do not carry a reference.

**Response**

**WE HAVE EXPLAINED THIS IN DETAIL SEVERAL TIMES THROUGHOUT THE MANUSCRIPT AND SPECIFICALLY DISCUSS THIS LIMITATION IN THE DISCUSSION.**

**LINE 181**

**“Each mitigation strategy was then judged on the impact it might have on restoring coverage of the intervention it was targeting (e.g., antenatal care, immunization) based on consultation with country experts and the available literature at that time.17-21”**

**LINE 252**

**“For identification of mitigation strategies and their impact on health service coverage rates, as previously described, each country team collated information on what mitigation activities were taking place and each mitigation strategy was judged on the impact it might have on restoring coverage based on the limited scientific evidence available on what might work in health settings,17-21 as well as expert opinion. Some mitigation measures were estimated to improve coverage due to the possibility that the mitigation measure would increase patients’ confidence that a visit to a health facility would be safe, such as visible hygiene and social distancing measures at health facilities or education campaigns reassuring the population that it was safe to visit health facilities and stressing the importance of continued care seeking, even during the pandemic. Other mitigation measures were thought to increase health care coverage by increasing availability of health work force (by involving community-level health workers or task shifting) and ensuring a reliable supply chain. Due to the challenges of calculating the impact of mitigation measures, a concerted effort was made to keep the impact estimates for individual mitigation measures conservative and the model included control features to ensure that overall coverage assumptions stayed within a defined range (e.g., coverage after mitigation measures were implemented was not allowed to be more than 2% above pre-pandemic coverage levels). S1 provides an overview over the most common mitigation strategies and estimates of their impact by country.”**

**LINE 344**

**“At the time of the study, no evaluation had been done yet of how mitigation measures affected coverage of essential RMNCH services during the COVID-19 pandemic. Estimates used in this analysis were thus mainly based on expert opinion. Slightly easier, but still not well documented, was the estimation of how mitigation measures might affect the risk of infection of virus transmission at a health facility.” ^-^**

**ALSO PLEASE NOTE REFENCES 17 TO 21 INCLUDE SYSTEMATIC REVIEWS OF WHAT WAS KNOW ABOUT INTERVENTIONS TO PREVENT CORONAVIRUS TRANSMISSION IN HOSPITAL SETTINGS AND WAS USED IN OUR MODELLING.**
